# Doxorubicin-loaded DNA origami nanostructures: stability in vitreous and their uptake and toxicity in ocular cells†

**DOI:** 10.1039/d4nr01995d

**Published:** 2024-08-27

**Authors:** Anna Klose, Zahra Gounani, Heini Ijäs, Tatu Lajunen, Veikko Linko, Timo Laaksonen

**Affiliations:** a Drug Research Program, Division of Pharmaceutical Biosciences, Faculty of Pharmacy, University of Helsinki Viikinkaari 5 00790 Helsinki Finland anna.klose@helsinki.fi timo.laaksonen@helsinki.fi; b Biohybrid Materials, Department of Bioproducts and Biosystems, Aalto University P.O. Box 16100 00076 Aalto Finland; c School of Pharmacy, University of Eastern Finland Yliopistonrinne 3 70210 Kuopio Finland; d Institute of Technology, University of Tartu Nooruse 1 50411 Tartu Estonia veikko.pentti.linko@ut.ee; e Chemistry and Advanced Materials, Faculty of Engineering and Natural Sciences, Tampere University Korkeakoulunkatu 8 33720 Tampere Finland

## Abstract

Biocompatibility and precise control over their size and shape make DNA origami nanostructures (DONs) promising for drug delivery applications. Whilst many investigations have focused on cancer treatment, this might not be the best fit for DONs that get degraded by nucleases in blood. In comparison, an eye is a uniquely isolated target organ, which could benefit from DONs to achieve and maintain therapeutic concentrations in diseases that threaten the eyesight of millions of patients every year. We investigated the loading of doxorubicin (DOX) as a model drug into three distinct DONs and tested their stability upon storage. Further, we chose one structure (24HB) to probe its stability under physiological conditions in cell media and porcine vitreous, before examining the uptake and effect of DOX-loaded 24HB (24HB-DOX) on the cell viability in a retinal cell line (ARPE-19). Similar to previous reports, the tested low μM loading concentrations of DOX resulted in high drug loadings of up to 34% (m/m), and remained mostly intact in water for at least 2 months at 4 °C. In cell media and porcine vitreous at 37 °C, however, 24HB required additional Mg^2+^ supplementation to avoid degradation and the loss of the attached fluorophores. With added Mg^2+^, 24HB remained stable in vitreous for 7 days at 37 °C. The treatment with 24HB-DOX was well tolerated by ARPE-19 cells, compared to the observed higher toxicity of free DOX. Uptake studies revealed, however, that in contrast to free DOX, very little 24HB-DOX was taken up by the cells. Instead, the particles were observed to attach around the cells. Hence, our results suggest that since the uptake seems to be the bottleneck for therapies using DONs, further strategies such as adding ocular targeting moieties are necessary to increase the uptake and efficacy of doxorubicin-loaded DONs.

## Abstract 2

Doxorubicin-loaded DNA origami required additional Mg^2+^ to be stable in cell medium and vitreous at 37 °C. In retinal epithelial cells (ARPE-19), they were well-tolerated, but limited in their uptake.

## Graphical abstract

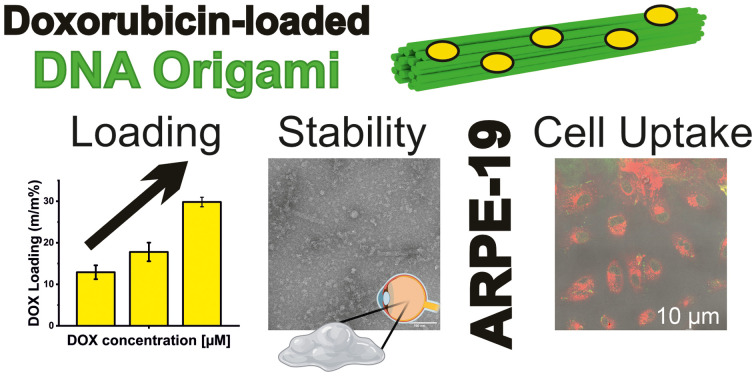

## Introduction

DNA origami nanostructures (DONs) are attractive nanocarriers in drug delivery applications because their dimensions and functionalities can be precisely designed, controlled and modified according to need.^1–5^ A single-stranded DNA scaffold strand of known sequence is folded into a desired DON shape by annealing with dozens of short, specifically designed, base-complementary staple strands under simple and reproducible conditions.^6–8^ Hence, DONs can be easily tailored to therapeutic applications, for instance, by fine-tuning their shape, size, surface charge and functionality to the preferences of the target cells.^3,9^ Furthermore, on their surface, DONs can display in a spatially-controlled manner targeting moieties, such as peptides,^10^ ligands,^11,12^ and aptamers,^13–18^ to enhance selective uptake and boost their drug delivery performance *in vitro*^11,12,14,15,19,20^ and *in vivo*.^17,18^ Yet, most importantly for biomedical applications, DONs are biodegradable and usually well tolerated in *in vitro* and *in vivo* systems.^21–23^

However, the stability of DONs under physiological conditions, namely in low-magnesium environments (<1 mM) and their susceptibility against nucleases in the blood, still remains a concern and needs to be investigated.^1^ Because DONs are most commonly made up of densely packed DNA helices, the negative charge of their phosphate backbone requires sufficient magnesium concentrations to screen the electrostatic repulsion and maintain their structural integrity.^24,25^ DONs’ stability against magnesium depletion and digestion by enzymes is, however, superstructure-dependent, and hence, can necessitate addition of magnesium to the media,^24^ structural redesign^26^ or coating strategies.^27–30^

Furthermore, while DONs can carry various therapeutic molecules, such as antibody fragments^31^ or nucleic acids,^10,32,33^ the anti-cancer drug doxorubicin (DOX) is one of the most widely investigated.^34^ As DOX is a DNA-intercalating drug,^35^ it allows straightforward formation of complexes with DONs (DOX-DONs), achieving relatively high drug loadings. DOX-DONs also have been effective in circumventing drug resistance,^36^ whilst exhibiting little toxicity *in vivo*.^37^ This is especially promising as to avoid the severe, dose-limiting side effects of DOX, which include cardiotoxicity.^38^ Yet, it remains important to always ensure proper characterization DOX-DONs as drug nanocarriers, especially considering that reported loading and purification protocols for DOX-DONs vary largely and often lack comparability, with a tendency to overestimate drug loading if no suitable purification method is employed.^39^

Thus far, investigations of DONs for drug delivery have mainly centered around systemic administration in cancer applications,^40^ where DOX-DONs can accumulate non-specifically through enhanced permeability and retention effects,^37^ but are continuously exposed to nucleases and immune cells in the entire body. We propose that DONs could be more compatible with local administration to an isolated, immune-privileged target organ like the eye.^41,42^ Because overcoming the blood-retinal barrier in systemic applications is challenging, and penetration upon topical instillation is poor,^41,43^ local intravitreal injections into the eye ball remain the most used approach to deliver therapeutics to the back of the eye. Upon such intravitreal injection, not only are DONs shielded from nucleases, but specifically their intrinsic negative charge and controlled size could be advantageous to their mobility in the vitreous body,^44^ a gel-like matrix (>98% water) from collagen and glycosaminoglycans that fills the inner eye.^45^ Hence, DONs could be promising for treating ocular diseases, but studies to assess their compatibility and applicability are necessary.

Currently, 4.3 million people worldwide are affected by blindness, but the total number could increase to 61 million by 2050.^46^ Amongst the leading causes of blindness, age-related macular degeneration (AMD) alone is affecting 67 million patients in Europe, and due to an aging population, is expected to impair 77 million by 2050, displaying similar trends globally.^47–49^ Intravitreal injections of anti-VEGF (vascular endothelial growth factor) treatments are effective in reducing neovascularization in the disease progress of AMD, or diabetic macular edema (DME). As a downside, however, they still need to be administered indefinitely every other month to uphold therapeutic concentrations.^50,51^ Besides the risk for side effects and burden on the healthcare system, these regular injections restrict and impact patients physically and psychologically, albeit necessary to preserve their eyesight.^43,52,53^ Hence, there is a need to find suitable, long-lasting, and effective therapeutic strategies and nanocarriers,^41^ and to this aim, also continue to explore and characterize novel nanocarriers like DONs with unique characteristics that have not been evaluated before for ocular implementations.

Thus, the control over their specific size and shape, and charge makes DNA-based nanocarriers interesting for investigations into ocular drug delivery and its different barriers. For instance, tetrahedral framework nucleic acids of defined size successfully reached the retina after periocular application.^54^ Here, we were interested to see whether larger and more dense DONs could also serve as suitable nanocarriers for ocular drug delivery. Currently, DOX is not very common for treating ocular diseases, but reports have shown its beneficial effects against choroidal neovascularization^55,56^ as a HIF1α-inhibitor in reducing pro-angiogenic factors, which is promising against AMD,^56,57^ or in its application against retinoblastoma.^58–62^ Also in these applications, encapsulating DOX into drug carriers can increase its efficiency and reduce local toxicity.^56–58,63^ Taking advantage of their multiple, densely packed DNA helices, DONs are expected to show high drug loading capacities of DNA-intercalating drugs such as DOX, and hence, here we investigated and characterized the DOX-loading into three differently shaped DONs (24-helix bundle (24HB), 60-helix bundle (60HB) and a rectangular plate structure) and their storage stability. Further, to elucidate their structural stability under physiological conditions of low Mg^2+^ concentrations, fluorophore-labeled 24HB was exposed and tested in cell medium and porcine vitreous. Thirdly, we evaluated the impact of DOX-loaded 24HB and free DOX on cell viability and studied their uptake into an ocular cell line (ARPE-19), to better understand the opportunities and challenges of using drug-loaded DONs for ocular delivery.

## Materials and methods

### DNA origami (DONs) preparation

**Fig. 1:**
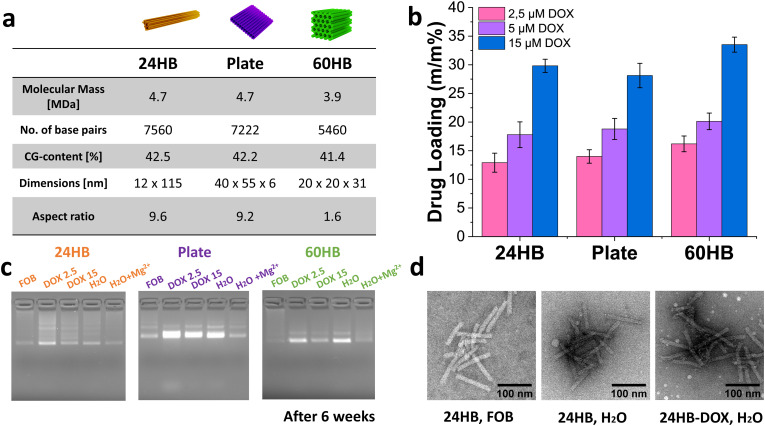
Doxorubicin (DOX) loading into DNA origami nanostructures (DONs). (a) Schematic representations and characteristics of the DONs (24-helix bundle (24HB), plate, 60-helix bundle (60HB)) investigated for DOX-loading. The aspect ratio was calculated by dividing the longest axis length by the shortest axis length. (b) Achieved drug loading (m/m%) of DOX into 24HB, plate, and 60HB DONs after spin-filtration, depending on initial DOX loading concentrations (2.5 μM, 5 μM, 15 μM). Mean ± s.d., *n* = 3. (c) Gel electrophoresis of DONs in folding buffer (FOB), DOX-DONs after loading and purification (DOX 2.5 and 15 μM), and DONs in H_2_O with and without MgCl_2_ addition to the loaded sample (corresponding to the Mg^2+^ concentration in FOB). The samples were stored for 6 weeks at 4 °C. (2% agarose with 0.46 μg mL^−1^ EtBr, cropped gel images). (d) Transmission electron microscopy images of 24HB in folding buffer (FOB), in deionized water (H_2_O), and after loading and purification with 15 μM DOX (24HB-DOX, H_2_O, negatively stained with 2% uranyl formate). 24HB, H_2_O and 24HB-DOX, H_2_O were imaged after 7 weeks of storage at 4 °C. Areas with several well-defined structures and good deposition from the respective grids were selected, cropped and contrast-adjusted.

To study the effect of DON superstructure (shape and size) on DOX-loading, three DONs (24HB, 60HB and the plate) were folded and purified from excess staples *via* polyethylene glycol (PEG) precipitation (ESI section SI1†).^30,39,64,65^ Details of the DONs are listed in Fig. 1a. For confocal imaging and stability testing, Atto488-labeled 24HBs were prepared. First, 24 staple strands were replaced in the 24HB folding reaction with staple strands that were extended at the 3′ end.^66^ Then in a second step, complementary strands carrying Atto488 at the 5′ end were annealed to these extensions by cooling from 40 °C to 20 °C in steps of – 0.1 °C/40 s, followed by the removal of excess Atto488-strands *via* PEG-precipitation (ESI section SI1†).^65,66^ Two versions of Atto488-labeled 24HB were tested, using either 17 nt long (24HB-Atto488 short),^66^ or 23 nt long Atto488-modified-strands (24HB-Atto488) with 16 nt or 22 nt long staple strand extensions, respectively (for details, ESI section SI2 and Table SI2b†). Their melting temperatures were estimated using the IDT OligoAnalyzer™ Tool (ESI Fig. SI2a†). After purification, all DONs were resuspended overnight on a shaker (at 30 °C, 600 rpm) in their respective folding buffer (1× FOB comprising of 1× Tris-acetate-EDTA buffer (1× TAE: 40 mM Tris, 20 mM acetic acid, 1 mM EDTA), and 17.5 mM MgCl_2_ for 24HB, or 20 mM MgCl_2_ and 5 mM NaCl for 60HB and the plate).

### Characterization of DONs

Agarose gel electrophoresis (AGE) and transmission electron microscopy (TEM) verified proper folding and purification, Atto488 attachment and stability of DONs (ESI section SI1 and Fig. SI3, 4†). Mixed with 6× loading dye solution (Sigma Aldrich), samples were loaded and run in 2% agarose gels with 0.46 μg mL^−1^ ethidium bromide (EtBr) or 1× SYBR Safe (Invitrogen) at 90 V for 50 min (running buffer: 1× TAE, 11 mM MgCl_2_). Gels were visualized under UV light (EtBr) or blue light (Atto488 and SYBR Safe) using BioRad ChemiDoc MP imaging System or Universal Hood II.

For TEM imaging, samples, fixated on plasma-cleaned Formvar carbon-coated copper grids (FCF-400-CU, Electron Microscopy Sciences), were negatively stained with 2% (w/v) uranyl formate solution (adjusted with 25 mM NaOH, ESI section SI1†),^8^ and imaged on FEI Tecnai 12 Bio-Twin electron microscope (120 kV acceleration voltage) or on Hitachi HT7800 Transmission Electron Microscope (100 kV acceleration voltage). Fiji-Image J was used to adjust the contrast or crop TEM images.

The hydrodynamic volume (intensity-weighed *Z*-average) and polydispersity index (PDI) of DONs was determined *via* dynamic light scattering (DLS) using an automated plate sampler (Zetasizer APS, Malvern Instruments).

DON concentration c (in mol L^−1^) was determined from its absorbance at 260 nm (A260), according to Lambert–Beer law (*A* = *εcl*, with a pathlength of *l* = 0.05 cm (for Take3™ plate reader) or nominally *l* = 10 mm (for NanoDrop One)). Based on the number of hybridized and non-hybridized nucleotides, the molar extinction coefficient *ε* was estimated for 24HB (1.05 × 10^8^ M cm^−1^), 24HB-Atto488 short (1.08 × 10^8^ M cm^−1^), 24HB-Atto488 (1.10 × 10^8^ M cm^−1^), 60HB (0.91 × 10^8^ M cm^−1^), and the plate (1.04 × 10^8^ M cm^−1^).^67^

### Buffer exchange and DOX loading into DONs

To reduce agglomeration, DONs were kept overnight on a shaker (30 °C, 600 rpm) before exchanging FOB to deionized water. Adapted from Kielar *et al.*,^25^ DONs (175 μL) were spun with water (175 μL) in pre-rinsed Amicon Ultra 0.5 mL centrifugal filters with 100 kDa molecular weight cut-off (MWCO) (Merck Millipore, 6000*g*, 10 min). After a second washing with water (314 μL, 6000*g*, 10 min), DONs were recovered (1000*g*, 2 min), diluted, and their concentration analyzed.

Doxorubicin hydrochloride (DOX, 579.99 g mol^−1^, CRS, European Pharmacopoeia Reference Standard) stocks in water (10 mM) were thawed and diluted to 100 μM working solutions. DOX loading reactions in water, consisting of 2 nM of DONs with 2.5 μM, 5 μM, or 15 μM DOX, were vortexed before their incubation at room temperature for at least an hour. DONs without DOX, and free DOX at corresponding concentrations served as references of similar composition. Before purifying DOX-DONs from excess DOX, their absorbance spectra (240–650 nm) were recorded in a quartz cuvette (*l* = 1 cm, Varian Cary 50 UV-Vis spectrophotometer). DOX-DONs (480 μL) were centrifuged in pre-rinsed Amicon Ultra 0.5 mL centrifugal filter with 100 kDa MWCO (6000*g*, 10 min).^39^ After two washes with water (480 μL, 6000*g*, 10 min), purified DOX-DONs were recovered (1000*g*, 2 min), and rediluted with water (460 μL). After purification, their absorbance spectra were measured again. Taking into account losses in spin-filtration and the absorption of DOX at 543 nm and of DONs at 260 nm, DOX-DONs drug loading (m/m%), drug loading efficiency, and DOX packing density were calculated (for details see ESI Fig. SI5 and Table SI6†).

### DON stability in water at 4 °C

DONs with or without loaded DOX were checked after a minimum of 6 weeks for signs of instability *via* AGE and TEM. DLS was used to monitor major aggregation and instability events over the course of 7 weeks (*n* = 3 with 2–3 measurements per sample).

### Porcine vitreous extraction

Porcine eyes on ice were procured from an abattoir (HKScan Finland Oyj, Finland) and their vitreous was isolated as previously described.^44^ In short, after removal of excess tissue and washing in 70% ethanol, the eyes were rinsed and stored in 1× Dulbecco's phosphate-buffered saline (DPBS, Gibco™) at 4 °C. The following day, the anterior part including the lens was excised with a scalpel to collect the vitreous. The vitreous was then homogenized on ice with a glass tissue homogenizer and centrifuged to sediment and remove melanin (3200*g*, 4 °C, 1 h). To the same aim, the decanted vitreous was also filtered through a 0.45 μM and 0.22 μM sterile filter (Sartorius, 16537 and K16532 K) before freezing aliquots at −80 °C. Before use, the vitreous was thawed overnight in the fridge.

### 24HB stability in cell medium and vitreous

Dulbecco's Modified Eagle Medium: Nutrient Mixture F-12 (DMEM/F12, HEPES, Gibco™, suitable for ARPE-19 cells, in the following abbreviated as cell medium (CM)), was adjusted with MgCl_2_ to CM-*x* with varying final Mg^2+^ concentrations (n. = no additional Mg^2+^, *x* = final Mg^2+^-concentration in mM, composition: 2% (v/v) Mg^2+^/H_2_O, 98% (v/v) CM). Then, buffer-exchanged 24HB in water (10% (v/v)) were incubated in CM-*x* (90% (v/v)) for 4 h or 24 h, at 37 °C or room temperature, respectively. 24HB-DOX, 24HB-Atto488 short and 24HB-Atto488 were also tested. Additionally, shorter incubations such as 2 h, varying dilution ratios (25% or 50%), and corresponding references in 1× FOB or water were prepared and investigated.

Further, homogenized porcine vitreous (V, 70 or 77% (v/v)) was incubated for up to 7 days with 24HB(-Atto488) in water (30 or 23% (v/v)) at 4 °C and 37 °C. The incubated samples were adjusted with MgCl_2_ to different to V-*x* (n. = no supplementation, *x* = final Mg^2+^-concentration in mM, without considering the preexisting Mg^2+^ content in vitreous). To check the integrity of 24HB after incubation, all samples were adjusted to similar final Mg^2+^ concentrations before running AGE. The vitreous samples were diluted (1 : 1) to reduce aggregation in the gel pocket before loading onto the gel. Selected samples were also fixated and visualized under TEM.

### Cell culturing

The ARPE-19 cells (human retinal pigment epithelial cell line) were a kind gift from Prof. Arto Urtti at the University of Eastern Finland, and originally purchased from the American Type Culture Collection (ATCC). The ARPE-19 cells were cultured at 37 °C with 5% or 7% CO_2_ in TC-treated T75 flasks (Sarstedt) with CM (DMEM/F12, HEPES, Gibco™), supplemented with 10% fetal bovine serum (FBS, Gibco™) and 1% penicillin–streptomycin (PS, Gibco™).

### Cell viability assay

10 000 cells per well were seeded overnight into TC-treated 96-well plates (Sarstedt). The next day, the cells were washed with 1× DPBS, exposed to treatments for 4 h, and after washing with 1× DPBS, kept again overnight in CM (10% FBS, 1% PS). After 20 h, the CM was replaced with 1× alamarBlue™ Cell Viability Reagent (Invitrogen™) in CM (1% PS), and incubated for 3 h at 37 °C. The alamarBlue mix was transferred to a black-walled wellplate (Thermo Scientific™, 265301) for fluorescence read-out (Varioskan LUX, Thermo Scientific™, top optics, *λ*_exc_ = 560 nm, *λ*_det_ = 590 nm). The assay was repeated three times on separate days with each three technical replicates, and the cell viability was expressed in % of viability in relation to the CM ref treatment (CM, 1% PS). The controls and treatments contained 24HB, free DOX or 24HB-DOX in CM with 1% PS, adjusted to a Mg^2+^-concentration of 3 mM after final dilution. CM 25% and CM 50% served as CM controls of similar composition but without any DONs or DOX. For 24HB-DOX, 24HB was loaded with 5 μM DOX, purified, upconcentrated and pooled after spin-filtration. For CM 25%, 24HB (5.3 nM), DOX (1–10 μM), and 24HB-DOX (1–10 μM), the treatments consisted of 25% (v/v) 24HB/DOX/H_2_O in 75% (v/v) CM-4 (4 mM MgCl_2_), while for CM 50%, 24HB (10.6 nM), DOX (20 μM), and 24HB-DOX (20 μM), they consisted of 50% (v/v) treatment and 50% (v/v) CM-6 (6 mM MgCl_2_). Triton X-100 1% in 1× DPBS served as a positive control.

### Confocal imaging

10 000 cells per well were seeded overnight to the Cellview cell culture slide (TC, glass bottom, 10 wells, Greiner, ref: 543078). The following day, the cells were washed with 1× DPBS and treated for 4 h or 24 h. The treatments contained 24HB-Atto488 (5.3 nM or 10.6 nM), Atto488-strands (254 nM, corresponding to the concentration for 24HB-Atto488 at 10.6 nM), free DOX (20 μM) or 24HB-Atto488-DOX (20 μM) in CM with 1% PS, adjusted to a Mg^2+^-concentration of 3 mM after final dilution. CM with similar dilution and composition without 24HB-Atto488 or DOX served as a reference. For 24HB-Atto488-DOX, 24HB-Atto488 was loaded with 5 μM DOX, purified, upconcentrated and pooled after spin-filtration. The treatments for 24HB-Atto488 at 5.3 nM concentration consisted of 25% (v/v) 24HB-Atto488/DOX/H_2_O and 75% CM-4 (4 mM MgCl_2_), or for 10.6 nM (and/or 20 μM DOX) of 50% (v/v) treatment and 50% CM-6 (6 mM MgCl_2_). The cells were stained at the end of the incubation time for 30 min with Hoechst 33258 pentahydrate (Invitrogen™), for 10 min with CellMask™ Deep Red plasma membrane stain (Invitrogen™) or for 1 h with LysoTracker™ Deep Red (Invitrogen™). After removal of the treatments, the cells were washed with 1× DPBS and replaced with CM (reference).

Samples were imaged using an inverted confocal laser scanning microscope (Leica TCS SP8 STED 3X CW 3D STED (Stimulated Emission Depletion), Germany). The 405 nm -diode laser was used for exciting Hoechst 33258, the argon 483 nm laser was used for exciting doxorubicin^68^ and Atto488, and the HeNe 633 nm laser was used for exciting LysoTracker™ Deep Red or CellMask™ Deep Red Plasma Membrane Stain. Images were obtained using a 63×/1.20 (water, wd 0.3 mm) objective, and analyzed using LAS X software version 3.7.4.23463 for Leica SP8.

## Results and discussion

### DOX-loading into DNA origami and DON storage stability

To investigate their drug loading capacity, three 3D DNA origami nanostructures (DONs) of different sizes and shapes, 24-helix bundle (24HB), 60-helix bundle (60HB), and plate, were loaded with the DNA-intercalating drug doxorubicin (DOX). After the incubation of 2 nM DONs in water with 2.5 μM, 5 μM or 15 μM DOX, and their subsequent purification from excess DOX *via* spin-filtration, the amount of DOX in DOX-loaded DONs (DOX-DONs) was determined *via* absorption measurements (ESI Fig. SI5†). Considering the recovery of DOX-DONs, drug loading was expressed in mass percent (m/m%) and compared for different DOX loading concentrations in different DONs.

In agreement with previous reports, similar drug loading was achievable for all DONs structures, regardless of their differences in size, shape, aspect ratio, or DNA sequence (Fig. 1a).^39^ This was possibly due to alike GC-contents of tested DONs, offering similar amounts of intercalation sites per structure.^35^ Most importantly, an increase in the initial DOX loading concentration resulted in higher drug loading after purification (Fig. 1b):^15,39^ A six-fold increase in the initial DOX loading concentration from 2.5 μM to 15 μM at least doubled the final drug loading, for instance for 60HB from 16% (m/m) to 34% (m/m), corresponding to a denser packing of DOX molecules per base pairs (ESI Table SI6†). In the case of 60HB with an initial 15 μM DOX loading concentration (before purification corresponding to one DOX molecule for every 0.7 base pairs), the previously reported maximum of one intercalated DOX molecule every two to three base pairs was exceeded even after purification, with one DOX molecule available every 1.5 base pairs.^39^ The intercalation capacity for 60HB was nearly maxed out already with one DOX molecule every 3 base pairs after purifying a ∼5 μM DOX loading reaction (before purification corresponding to one DOX molecule every 2.2 base pairs). Therefore, the observed result suggested another mode of DOX binding and aggregation on top of DONs, contributing in part to its loading capacity.^35^ In line with these findings, loading efficiencies which expressed how much of the initially added DOX was loaded into the DONs, decreased with higher DOX loading concentrations from ∼76–84% for 2.5 μM DOX to ∼35–40% for 15 μM DOX (ESI Table SI6†), congruent with free DOX being removed in the purification step. Our final loaded DOX concentrations were in good agreement with reports that used similar loading conditions.^39,69^

While high loading efficiencies for DOX-DONs are widely acknowledged, the reported efficiencies vary between 7–71%.^13–15,36,37,69–71^ For instance, introducing specific twists into DONs can allow fine-tuning of DOX encapsulation and release.^72^ Yet, the variety in the ways for reporting DOX loadings – such as loading efficiency, DOX concentration, DOX per DON, or DOX per base pair – in combination with different and underreported DOX loading, purification and detection methods, limits and complicates the direct comparability.^39^ In comparison to our spin-filtering method, removal of free DOX *via* centrifugation often risks retaining DOX aggregates alongside of DOX-DONs and overestimating the actual DOX loading.^39^ In such case, DOX loading efficiencies would be skewed, being rather a reflection of the initial DOX loading concentration, and loading and purification conditions, than of the actual DOX-loading capacity of DONs. To ensure that all DOX was bound to DNA in further *in vitro* studies, we accepted lower total DOX loadings by using DOX loading concentrations within low μM, instead of mM range,^13,14,36,37,70^ and by opting to report drug loading (m/m%, ESI Fig. SI6†).

Importantly, most DONs and DOX-DONs maintained their structural integrity and electrophoretic mobility in deionized water at 4 °C for at least 6 weeks, as observed *via* agarose gel electrophoresis (AGE) and under the transmission electron microscope (TEM, Fig. 1c, d and ESI Fig. SI7–9†).^25,64^ The same discrete bands on the agarose gels for DONs and purified DOX-DONs confirmed that most of our DONs remained intact, and no shift for our DOX-DONs band was observable upon DOX loading (Fig. 1c). Only for the DOX-plate, a widened band hinted at some aggregation and some slight staple strand loss during storage.

While some imperfect and unraveled DONs were visible in TEM (Fig. 1d and ESI Fig. SI7, 8†), possibly also due to mechanical stress during spin-filtration, most retained their shape after DOX-loading,^10,70^ even though intercalated DOX can exert strain on DONs.^12,72^ In addition, buffer exchanges to low Mg^2+^ environments, such as water or cell media, can destabilize DONs,^25^ because sufficient Mg^2+^ ions are vital to screen the negative charges of their DNA phosphate backbones, reducing repulsion effects and allowing for compact DONs. In such case, more rigid DON structures like the 24HB, that are structurally supported by many crossovers in their staple and scaffold design, are more dependent on Mg^2+^ ions to ensure their stability.^26,73^ While some other ions and polymers can also stabilize DONs,^74^ low Mg^2+^ concentrations can cause local degradation in DONs, which, however, can self-repair once favorable ionic conditions are attained again.^75^

Additionally, no major aggregation and instability events, were detectable *via* dynamic light scattering (DLS). The polydispersity index (PDI) for all DONs started increasing only after 4 weeks of storage (ESI Fig. SI10†). This increase possibly indicated that during longer storage, DONs started interacting more with each other and forming aggregates,^64^ which depending on the structure could be harder to redisperse and lead also to multiple bands on AGE. Since 24HB appeared the most stable, it was selected for further *in vitro* studies.

### 24HB stability testing in cell media and porcine vitreous

**Fig. 2:**
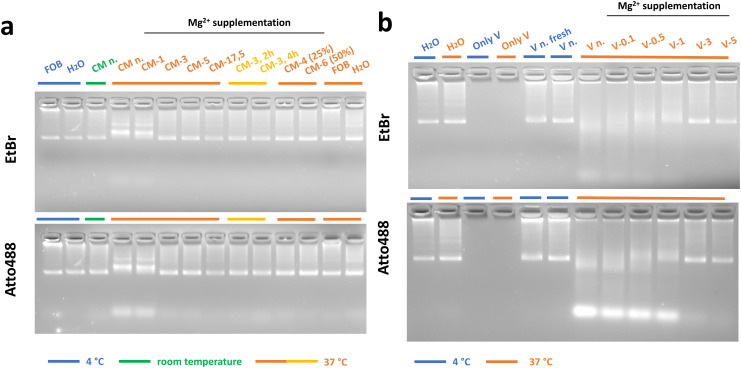
Stability of 24HB-Atto488 in cell medium (CM) and in porcine vitreous (V) at 37 °C for 24 h. Agarose gel electrophoresis of samples incubated at 4 °C (blue), room temperature (green) and 37 °C (yellow/orange) for 24 h and detected under UV light (EtBr channel) or blue light (Atto488 channel). (a) 24HB-Atto488 was incubated in folding buffer (FOB, reference), deionized water (H_2_O, reference) and CM-*x*, supplemented with increasing amounts of MgCl_2_ (n. = no supplementation, *x* = Mg^2+^ concentration in mM). CM-*x* samples comprised of 90% CM-*x* and 10% 24HB-Atto488/H_2_O, except for CM-4 (25%) and CM-6 (50%) diluted 25% and 50% (v/v), respectively. 24HB-Atto488 in CM-3 was also incubated for 2 h and 4 h. All samples were adjusted to the same final Mg^2+^ concentration before running the gel. (b) 24HB-Atto488 was incubated in deionized water (H_2_O, reference) or in porcine vitreous (V-*x*), supplemented with increasing amounts of MgCl_2_ (n. = no supplementation, *x* = Mg^2+^ concentration in mM added to the incubation reaction). V-*x* samples comprised of 70% (v/v) V-*x* and 30% (v/v) 24HB-Atto488/Mg^2+^/H_2_O. “V n. fresh” was prepared before running the gel with the same V and 24HB-Atto488 as the other samples without any incubation. “Only V” contained diluted vitreous without 24HB-Atto488 (2% agarose gel, 0.46 μg mL^−1^ EtBr, cropped gel images).

In preparation for *in vitro* studies, 24HB with Atto488-fluorophores, attached *via* extended staple strands that hybridized with Atto488 strands (24HB-Atto488, ESI section SI2†), was probed for its structural integrity at 37 °C. To mimic the low-nuclease environment of the eye,^76^ 24HB-Atto488 underwent 24 h-incubation at 37 °C in various dilutions of cell media (CM), revealing that additional Mg^2+^ supplementation to a minimum of 3 mM concentration (CM-3) was necessary to effectively stabilize the 24HB structure (Fig. 2a, EtBr-channel).^24^ 23 nt-long staple extensions were required for a stabile attachment of the Atto488 strands under incubation at 37 °C in CM-3 (Fig. 2a, Atto-channel, ESI section SI2†). Shorter extensions (17 nt) led to significant losses of Atto488 strands already after 4 h at 37 °C in CM-3 (SI11†). Apart from 24HB itself requiring Mg^2+^ supplementation for stabilization, Mg^2+^ addition was still relevant to maintain the fluorophore attachment at 37 °C in CM. Increasing the DNA melting temperature in complex, high ionic environments by simply elongating fluorophore attachment strands is therefore a useful but not complete solution, although attachments of similar or even shorter length have been reported.^3,12,18,24,77,78^ Another possible solution to reduce the loss of fluorophores could be incorporating the fluorophore directly into the DONs by attaching it to the staple strands, though no systematic investigations in that regard are available.^79^

Hahn *et al.* described similarly that DONs’ stability in CM (with <1 mM Mg^2+^) is time- and structure-dependent, but structural integrity and fluorophore attachment could be maintained with Mg^2+^ addition at 6 mM concentration, without any impact on *in vitro* studies.^24^ Since no Mg^2+^ addition was necessary if the 24HB-Atto488 in CM was incubated at room temperature, the various ions in cell media together with the higher temperature led to destabilizing interactions, if not sufficient Mg^2+^ was available to counterbalance them, like in FOB for instance. Remarkably though, 24HB-Atto488 was stable in water at 37 °C without any additional Mg^2+^. As previously discussed, low Mg^2+^ buffers can contribute to the destabilization of DONs if certain components in buffers like phosphate ions or EDTA can chelate Mg^2+^ ions.^25^ In the case of CM, however, the phosphate concentration is only ∼0.9 mM. Instead, abundant ion species in CM, such as sodium ions (∼136 mM) can be exchanged with Mg^2+^ ions on the phosphate backbone, thus, destabilizing the DON in a similar manner,^25,26,74^ but not in water. We similarly carried out stability testing for DOX-loaded 24HB-Atto488 structures: 24HB-Atto488-DOX appeared also stable in CM with 3 mM Mg^2+^, but not in water at 37 °C, illustrating that at higher temperatures, there was a more pronounced, destabilizing effect of DOX on the DONs structure (ESI Fig. SI12†). Further stabilization strategies to address this issue could involve increasing the crossover spacing in DONs to enhance their flexibility,^26^ or providing alternative stabilizing ions or polymeric coatings that can interact with the DNA phosphate backbone.^3,27,74,80^ Even though the stability of DONs is, in general, highly structure-dependent,^25^ stability testing is often alas superficially performed and reported, and sometimes does not reflect the subsequent *in vitro* conditions. This is especially relevant for uptake studies with fluorophore-labeled DONs, in which loss and possible uptake of degraded and free fluorophores can lead to misinterpretation.^81^

For more biorelevant stability testing, porcine vitreous at 37 °C was used. Here, the 24HB-Atto488 similarly required a minimum of 3 mM Mg^2+^ addition for stabilization (Fig. 2b and ESI Fig. SI13†). This is without considering any contribution of pre-existing Mg^2+^ in the vitreous (in humans around 0.84–0.96 mM Mg^2+^)^82^ that could amount to an additional ∼0.6–0.7 mM Mg^2+^ per sample. Upon 3 mM Mg^2+^ supplementation *in vitro*, however, 24HB-Atto488 even remained stable in vitreous for up to seven days (ESI Fig. SI14†), which is a reasonable residence time in the eye. While the reported residence times (hours to months) vary for nanoparticles depending on their properties and the animal model, 50 nm liposomes in rabbit eyes exhibited for instance an elimination half-life of 8 days.^83^ However, it seems difficult to suggest Mg^2+^ supplementation as a feasible stabilization strategy for DONs in *in vivo* settings, as small Mg^2+^ ions will be cleared out quicker than DONs. This would mean that for intravitreal application, selecting DONs that can better withstand low Mg^2+^ conditions, or further protective coatings could be needed to ensure structural integrity.^27,74^

**Fig. 3:**
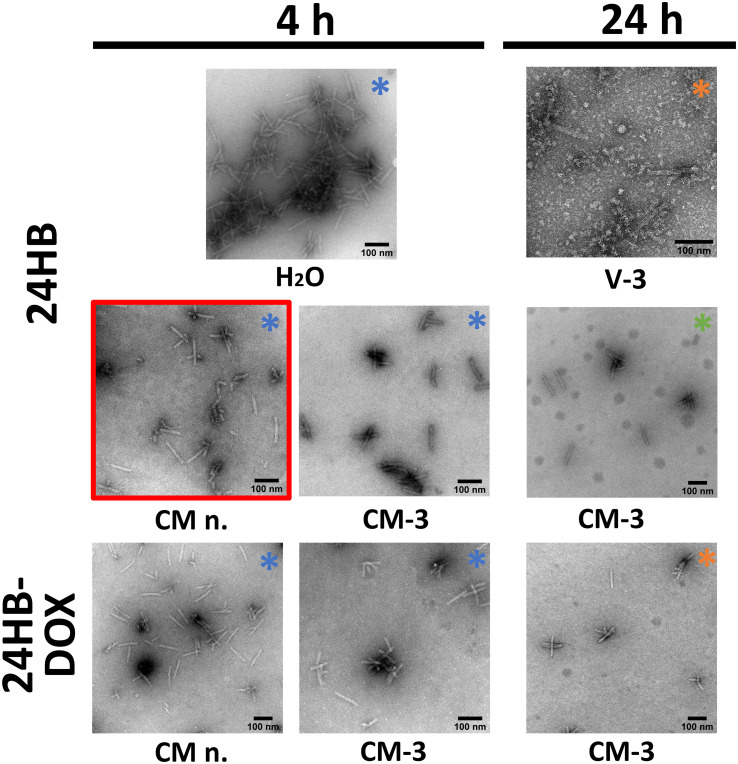
Transmission electron microscopy (TEM) images of 24HB and DOX-loaded 24HB (24HB-DOX), incubated in cell medium (CM-*x*) and in porcine vitreous (V-*x*) with and without MgCl_2_ supplementation at 37 °C (4 h *vs.* 24 h). n. = no Mg^2+^ supplementation, *x* = Mg^2+^ concentration in mM. The color of the asterix (*) indicates if the imaged 24HB had an Atto488 attachment, that was not visible under TEM (orange = plain 24HB (no Atto488), blue = 24HB-Atto488 short, green = 24HB-Atto488). Representative areas with several well-defined structures and good deposition from the respective grids were selected and contrast-adjusted, the scale bar represents 100 nm. The samples were negatively stained with 2% uranyl formate.

TEM further revealed that at 37 °C, 24HB in CM without additional Mg^2+^ (CM n.) degraded into smaller, yet distinct fragments within 4 h (Fig. 3, red frame). Possibly, 24HB has some particularly rigid segments, where, under low Mg^2+^ conditions, the electrostatic repulsion between the DNA helices strained specific staple strands. This could have resulted in their dissociation, and the characteristic fragment pattern.^73^ In comparison, supplementing CM and vitreous with 3 mM Mg^2+^ mostly preserved the 24HB structure for 24 h. Interestingly, regardless of the widened band on the gel (ESI Fig. SI12†), 24HB-DOX in CM n. appeared less fractured under TEM, compared to the 24HB without DOX. This was unexpected since DOX as an intercalator, often does introduce strain into DONs,^72^ and on our agarose gels, appeared to be rather destabilizing like in the case of 24HB-DOX in CM n. and water (ESI Fig. SI12†), indicating that additional Mg^2+^ is necessary for stabilization. After investigating its structural integrity under *in vitro* conditions, 24HB-DOX was next screened in ARPE-19 cells for any toxic effect and their uptake behavior.

### Treatment of an ocular cell line with DOX-loaded 24HB

**Fig. 4:**
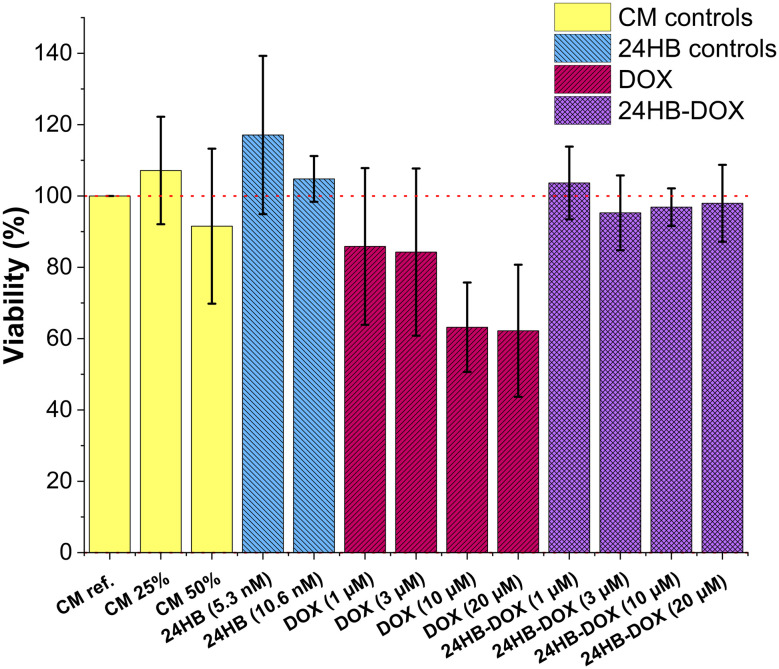
Cell viability of ARPE-19 cells treated with 24HB, free DOX and DOX-loaded 24HB. As controls served cell media with 1% PS (CM ref., set to 100% viability, red dotted line), CM 25% (CM ref. with 3 mM final MgCl_2_ concentration after 25% dilution, equivalent for: 24HB (5.3 nM), DOX (1–10 μM) and 24HB-DOX (1–10 μM)), and CM 50% (CM ref. with 3 mM final MgCl_2_ concentration after 50% dilution, equivalent for: 24HB (10.6 nM), DOX (20 μM), 24HB-DOX (20 μM)). ARPE-19 were treated with plain 24HB (blue, control), free DOX (red) and equivalent 24HB-DOX (purple) for 4 h, before determining the cell viability the following day *via* alamarBlue assay. Mean ± s.d., *n* = 3 (with each three technical replicates).

To assess the effect on their cell viability, ARPE-19 cells, a human retinal pigment epithelial cell line, were treated for 4 h with pooled 24HB-DOX, achieving final DONs concentration of up to 10.6 nM and DOX concentrations between 1–20 μM. The metabolic activity of the cells was then analyzed the following day *via* alamarBlue assay (Fig. 4). Further, ARPE-19 treated with undiluted CM with 1% PS (CM ref., yellow) was set to 100% viability, while CM treatments accounting for dilution and Mg^2+^ supplementation (CM 25%, 50%, also in yellow), and plain 24HB in two concentrations (24HB (5.3 nM, 10.6 nM), blue) served as controls, revealing that the chosen conditions did not impact the viability of the cells. This concurred with previous reports that plain DONs are well tolerable for cells.^12,14^ In contrast, ARPE-19 cells treated with free DOX (red) displayed at concentrations above 10 μM reduced cell viability by approximately 40%. At corresponding DOX concentrations, however, none of the 24HB-DOX treatments (purple) led to a decrease in cell viability, and instead, reduced the toxicity of DOX. Despite DOX not being a very commonly used drug in ocular diseases, when it is applied, a major concern is reducing its toxicity by avoiding high local concentrations and precipitation on the retinal surface.^56^ This is attainable by encapsulating DOX into particles, which also could positively improve the residence time in the eye.^56,57,63,84^

Generally, reported toxicities for DOX-DONs vary because of the differences in experimental conditions, concerning the type of assay, treatment duration (hours to days), or drug-susceptibility of the cell line. Here, the short-time exposure of the ocular cell line ARPE-19 to DOX in FBS-free media did not significantly affect its metabolic activity. Focusing first on the toxic effect of free DOX in ARPE-19, some reports showed viabilities in a similar range.^57,85,86^ On the other hand, some report IC50 values between 0.13–1 μM DOX after 48–72 h DOX exposure, which is considerably more toxic, but understandable, as longer exposure can lead to higher uptake and therefore also higher toxicity.^87–89^ However, there are differences between cell lines, and in some cases DOX-DONs have performed similarly^14,37,70^ or worse^15^ than free DOX, while in others, DOX-DONs even increased toxicity by promoting intracellular DOX accumulation.^36,72,79^

**Fig. 5:**
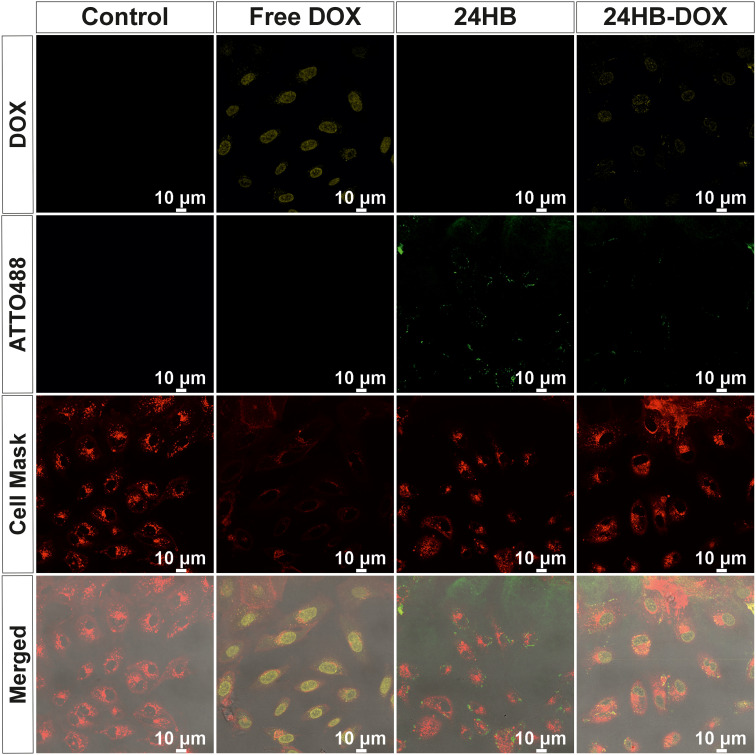
Live confocal imaging for uptake of 24HB-Atto488, free DOX and 24HB-Atto488-DOX after 24 h. ARPE-19 were treated with cell medium (CM, reference), 24HB-Atto488 (10.6 nM), free DOX (20 μM) or 24HB-Atto488-DOX (equivalent to 20 μM DOX), consisting of CM with 1% PS supplemented to a final 3 mM Mg^2+^ concentration after a 1 : 1 dilution with the treatments. Red = cell membrane (Cell Mask), green = 24HB-Atto488, yellow = DOX. The control and 24HB treated cells were not imaged in the DOX-channel because they did not contain any DOX. The DOX contrast was equally adjusted in all panels for better visualization. The merged images (bottom row of the panel) include the bright field channel in addition to the DOX, ATTO488 and Cell Mask channel for better visualization of the cells.

To elucidate its drug-delivering capacities, we investigated the uptake 24HB-Atto488 into the ARPE-19 cells *via* live confocal imaging. After 4 h, no colocalization between 24HB-Atto488 and the lysosomes was observable, but instead, 24HB-Atto488 was clearly visible outside of the cells (ESI Fig. SI15†). Similarly, even after 24 h, the uptake of 24HB-Atto488-DOX (20 μM) was limited (Fig. 5 and ESI Fig. SI16†). Only few 24HB-Atto488 (green) could breach the cell membrane (red) within 24 h, while it posed no barrier to free DOX (20 μM, yellow) that was localized in the nucleus as expected. In the case of 24HB-Atto488-DOX (20 μM), some DOX was also evident in the nucleus (faint yellow signal), but visibly less than in the case of free DOX treatment. While this could stem from some premature DOX release, we have observed previously using steady-state and time-resolved fluorescence anisotropy measurements that our DOX-loading and purification protocols ensure that all DOX present in the sample remained bound to DONs.^90^ 24HB-Atto488 were mostly seen at the bottom surface of the wells around the cells (ESI Fig. SI17–19†). In contrast to our observations, many publications have reported that DONs end up in the cytosol after escaping the lysosomes, while DOX then becomes apparent in the nucleus.^14,36,70,72,77,91^ Since close to none of the Atto488-strands by itself were taken up by cells (ESI Fig. SI20†), it confirmed that the green signal inside of the cells came from uptaken 24HB-Atto488, and their adhesion to the cell membrane was not mediated by the fluorophore. This, in combination with the low toxicity observed for 24HB-DOX, underlined that the uptake of 24HB-Atto488 was simply not very efficient, and hence contributed to the reduced DOX toxicity.

This limited uptake is plausible, as the inherent negative charge of DONs can hinder their uptake.^11,12,79^ While the uptake behavior of certain cells such as macrophages is unaffected by charge,^3^ often polymeric or protein coatings on DONs are necessary to facilitate cell entry.^3,27,28,92^ Generally, uptake behaviors and kinetics can depend on the cell line, with for instance specialized immune cells outperforming epithelial or endothelial cells by far.^3,9^ This complicates inferring from DONs uptake studies performed in other cell lines than ARPE-19, but it would offer an explanation for the low efficiency in cell entry that needs to be improved.

The dimensions of DONs is another factor influencing their cellular uptake: systematic investigations showed that cells seem to prefer compact and big DONs for endocytosis, with a low aspect ratio and directly structure-incorporated fluorophores.^9^ While 24HB-Atto488 is compact, and DON of similar dimensions have been internalized by cells,^78^ it is also quite stretched with Atto488 annealed *via* staple strand extensions, and hence, not exactly matching these criteria. While uptake kinetics are cell line-dependent and uptake increases for some after longer incubation,^9,12,78^ this was not observable for ARPE-19 within 24 h. While ARPE-19 cells tolerated the prolonged Mg^2+^-supplemented DONs treatment, the addition of FBS would be advisable to maintain cell health for monitoring the uptake over even longer periods. But, since ARPE-19 can effectively take up folate-targeted nanoparticles of up to 250 nm in size within 4 h,^93^ this rather suggests a low internalization efficiency of negatively charged 24HB itself, and the missing colocalization with lysosomes possibly hinted at the lack of receptor-mediated uptake.

Unfortunately, our knowledge on how DONs enter cells is still lacking. Some of the uncertainty is due to the variability of experimental conditions *in vitro* in published works, varying choice of DONs and cell lines, and originate from underreporting. However, in the case of effectively uptaken DONs, receptor-mediated uptake pathways,^94^*via* scavenger receptors,^79,95^ or caveolin-dependent,^78,79^ or lipid-raft mediated^14^ endocytosis have been suggested as uptake route. Hence, incorporating targeting moieties, such as aptamers,^14–19,69^ folate,^12,20^ and cell penetrating peptides,^10^ can improve uptake, which could be also a promising approach for our DONs to ARPE-19 cells and possibly other ocular cell lines.^55,93^ Especially since through receptor binding, spatial distribution of aptamers on DONs can regulate cell uptake and increase target specificity, which can offer unique optimization opportunities.^13^

In summary, we showed that DONs could effectively be loaded with DOX, independent of their superstructure, and remained stable under physiological conditions in cell media and in vitreous upon Mg^2+^ supplementation. Further, in ARPE-19 cells, DONs and DOX-DONs were well tolerated compared to free DOX. However, the uptake of DOX-DONs into cells was very low, warranting additional strategies such as coatings and targeting moieties to increase the efficiency of cell uptake for such DONs.

## Conclusions

DONs are promising nanocarriers in drug delivery applications, for the versatility and predictability of their structure allows precise tailoring and optimization to improve their drug delivering capacities. This makes DONs also interesting for intraocular drug delivery, where therapeutic drug concentrations are hard to achieve and maintain, but where the eye presents a uniquely isolated target organ. DONs intercalated with DOX allowed for desirably high drug loadings, whilst also demonstrating structural integrity over an extended storage period. Stability testing revealed emerging structural defects under physiological conditions at 37 °C in cell media and porcine vitreous of the eye, that were, however, addressable with additional Mg^2+^ supplementation. DOX-DONs showed no toxicity in a retinal cell line (ARPE-19), compared to free DOX, which could be favorable in reducing irritation and side-effects during application. Their uptake into ARPE-19 cells was, however, quite limited, warranting additional strategies to improve their uptake. We envision for instance, that incorporating specific targeting moieties onto DONs could help overcome this limited uptake and even contribute to selective uptake by specific target cells, making them more efficient for ocular drug delivery.

## Data availability

The data supporting this article has been included as part of the ESI.†

## Conflicts of interest

There are no conflicts to declare.

## Supplementary Material

NR-016-D4NR01995D-s001

NR-016-D4NR01995D-s002

NR-016-D4NR01995D-s003

## References

[cit1] Keller A., Linko V. (2020). Angew. Chem., Int. Ed..

[cit2] Douglas S. M., Marblestone A. H., Teerapittayanon S., Vazquez A., Church G. M., Shih W. M. (2009). Nucleic Acids Res..

[cit3] Koga M. M., Comberlato A., Rodríguez-Franco H. J., Bastings M. M. C. (2022). Biomacromolecules.

[cit4] Dey S., Fan C., Gothelf K. V., Li J., Lin C., Liu L., Liu N., Nijenhuis M. A. D., Saccà B., Simmel F. C., Yan H., Zhan P. (2021). Nat. Rev. Methods Primers.

[cit5] Tørring T., Voigt N. V., Nangreave J., Yan H., Gothelf K. V. (2011). Chem. Soc. Rev..

[cit6] Rothemund P. W. K. (2006). Nature.

[cit7] Douglas S. M., Dietz H., Liedl T., Högberg B., Graf F., Shih W. M. (2009). Nature.

[cit8] Castro C. E., Kilchherr F., Kim D.-N., Shiao E. L., Wauer T., Wortmann P., Bathe M., Dietz H. (2011). Nat. Methods.

[cit9] Bastings M. M. C., Anastassacos F. M., Ponnuswamy N., Leifer F. G., Cuneo G., Lin C., Ingber D. E., Ryu J. H., Shih W. M. (2018). Nano Lett..

[cit10] Wang Z., Song L., Liu Q., Tian R., Shang Y., Liu F., Liu S., Zhao S., Han Z., Sun J., Jiang Q., Ding B. (2021). Angew. Chem., Int. Ed..

[cit11] Ge Z., Guo L., Wu G., Li J., Sun Y., Hou Y., Shi J., Song S., Wang L., Fan C., Lu H., Li Q. (2020). Small.

[cit12] Pal S., Rakshit T. (2021). Front. Chem..

[cit13] Liu K., Xu C., Liu J. (2020). J. Mater. Chem. B.

[cit14] Pan Q., Nie C., Hu Y., Yi J., Liu C., Zhang J., He M., He M., Chen T., Chu X. (2020). ACS Appl. Mater. Interfaces.

[cit15] Chaithongyot S., Duangrat R., Wootthichairangsan C., Hanchaina R., Udomprasert A., Kangsamaksin T. (2020). Mater. Lett..

[cit16] Sun P., Zhang N., Tang Y., Yang Y., Zhou J., Zhao Y. (2018). RSC Adv..

[cit17] Liu J., Song L., Liu S., Zhao S., Jiang Q., Ding B. (2018). Angew. Chem., Int. Ed..

[cit18] Liu J., Song L., Liu S., Jiang Q., Liu Q., Li N., Wang Z.-G., Ding B. (2018). Nano Lett..

[cit19] Chen R., Sun P., Chu X., Pu X., Yang Y., Zhang N., Zhao Y. (2020). Int. J. Nanomed..

[cit20] Navarro N., Aviñó A., Domènech Ò., Borrell J. H., Eritja R., Fàbrega C. (2024). Nanomedicine.

[cit21] Du R. R., Cedrone E., Romanov A., Falkovich R., Dobrovolskaia M. A., Bathe M. (2022). ACS Nano.

[cit22] Lucas C. R., Halley P. D., Chowdury A. A., Harrington B. K., Beaver L., Lapalombella R., Johnson A. J., Hertlein E. K., Phelps M. A., Byrd J. C., Castro C. E. (2022). Small.

[cit23] Schüller V. J., Heidegger S., Sandholzer N., Nickels P. C., Suhartha N. A., Endres S., Bourquin C., Liedl T. (2011). ACS Nano.

[cit24] Hahn J., Wickham S. F. J., Shih W. M., Perrault S. D. (2014). ACS Nano.

[cit25] Kielar C., Xin Y., Shen B., Kostiainen M. A., Grundmeier G., Linko V., Keller A. (2018). Angew. Chem., Int. Ed..

[cit26] Xin Y., Piskunen P., Suma A., Li C., Ijäs H., Ojasalo S., Seitz I., Kostiainen M. A., Grundmeier G., Linko V., Keller A. (2022). Small.

[cit27] Ponnuswamy N., Bastings M. M. C., Nathwani B., Ryu J. H., Chou L. Y. T., Vinther M., Li W. A., Anastassacos F. M., Mooney D. J., Shih W. M. (2017). Nat. Commun..

[cit28] Auvinen H., Zhang H., Nonappa, Kopilow A., Niemelä E. H., Nummelin S., Correia A., Santos H. A., Linko V., Kostiainen M. A. (2017). Adv. Healthcare Mater..

[cit29] Perrault S. D., Shih W. M. (2014). ACS Nano.

[cit30] Julin S., Nonappa, Shen B., Linko V., Kostiainen M. A. (2021). Angew. Chem., Int. Ed..

[cit31] Douglas S. M., Bachelet I., Church G. M. (2012). Science.

[cit32] Liu S., Jiang Q., Zhao X., Zhao R., Wang Y., Wang Y., Liu J., Shang Y., Zhao S., Wu T., Zhang Y., Nie G., Ding B. (2021). Nat. Mater..

[cit33] Wu X., Liu Q., Liu F., Wu T., Shang Y., Liu J., Ding B. (2021). Nanoscale.

[cit34] Zhang Y., Tian X., Wang Z., Wang H., Liu F., Long Q., Jiang S. (2023). Front. Mol. Biosci..

[cit35] Pérez-Arnaiz C., Busto N., Leal J. M., García B. (2014). J. Phys. Chem. B.

[cit36] Jiang Q., Song C., Nangreave J., Liu X., Lin L., Qiu D., Wang Z.-G., Zou G., Liang X., Yan H., Ding B. (2012). J. Am. Chem. Soc..

[cit37] Zhang Q., Jiang Q., Li N., Dai L., Liu Q., Song L., Wang J., Li Y., Tian J., Ding B., Du Y. (2014). ACS Nano.

[cit38] Pai V. B., Nahata M. C. (2000). Drug Saf..

[cit39] Ijäs H., Shen B., Heuer-Jungemann A., Keller A., Kostiainen M. A., Liedl T., Ihalainen J. A., Linko V. (2021). Nucleic Acids Res..

[cit40] Zhang S., Lou X.-Y., Liu L., Yang Y.-W. (2023). Adv. Healthcare Mater..

[cit41] Del Amo E. M., Rimpelä A.-K., Heikkinen E., Kari O. K., Ramsay E., Lajunen T., Schmitt M., Pelkonen L., Bhattacharya M., Richardson D., Subrizi A., Turunen T., Reinisalo M., Itkonen J., Toropainen E., Casteleijn M., Kidron H., Antopolsky M., Vellonen K.-S., Ruponen M., Urtti A. (2017). Prog. Retinal Eye Res..

[cit42] Forrester J. V., Xu H. (2012). Front. Immunol..

[cit43] Martin D. F. (2018). Am. J. Ophthalmol..

[cit44] Tavakoli S., Kari O. K., Turunen T., Lajunen T., Schmitt M., Lehtinen J., Tasaka F., Parkkila P., Ndika J., Viitala T., Alenius H., Urtti A., Subrizi A. (2021). Mol. Pharm..

[cit45] Käsdorf B. T., Arends F., Lieleg O. (2015). Biophys. J..

[cit46] Bourne R., Steinmetz J. D., Flaxman S., Briant P. S., Taylor H. R., Resnikoff S., Casson R. J., Abdoli A., Abu-Gharbieh E., Afshin A., Ahmadieh H., Akalu Y., Alamneh A. A., Alemayehu W., Alfaar A. S., Alipour V., Anbesu E. W., Androudi S., Arabloo J., Arditi A., Asaad M., Bagli E., Baig A. A., Bärnighausen T. W., Battaglia Parodi M., Bhagavathula A. S., Bhardwaj N., Bhardwaj P., Bhattacharyya K., Bijani A., Bikbov M., Bottone M., Braithwaite T., Bron A. M., Butt Z. A., Cheng C.-Y., Chu D.-T., Cicinelli M. V., Coelho J. M., Dagnew B., Dai X., Dana R., Dandona L., Dandona R., Del Monte M. A., Deva J. P., Diaz D., Djalalinia S., Dreer L. E., Ehrlich J. R., Ellwein L. B., Emamian M. H., Fernandes A. G., Fischer F., Friedman D. S., Furtado J. M., Gaidhane A. M., Gaidhane S., Gazzard G., Gebremichael B., George R., Ghashghaee A., Golechha M., Hamidi S., Hammond B. R., Hartnett M. E. R., Hartono R. K., Hay S. I., Heidari G., Ho H. C., Hoang C. L., Househ M., Ibitoye S. E., Ilic I. M., Ilic M. D., Ingram A. D., Irvani S. S. N., Jha R. P., Kahloun R., Kandel H., Kasa A. S., Kempen J. H., Keramati M., Khairallah M., Khan E. A., Khanna R. C., Khatib M. N., Kim J. E., Kim Y. J., Kisa S., Kisa A., Koyanagi A., Kurmi O. P., Lansingh V. C., Leasher J. L., Leveziel N., Limburg H., Majdan M., Manafi N., Mansouri K., McAlinden C., Mohammadi S. F., Mohammadian-Hafshejani A., Mohammadpourhodki R., Mokdad A. H., Moosavi D., Morse A. R., Naderi M., Naidoo K. S., Nangia V., Nguyen C. T., Nguyen H. L. T., Ogundimu K., Olagunju A. T., Ostroff S. M., Panda-Jonas S., Pesudovs K., Peto T., Quazi Syed Z., Rahman M. H. U., Ramulu P. Y., Rawaf S., Rawaf D. L., Reinig N., Robin A. L., Rossetti L., Safi S., Sahebkar A., Samy A. M., Saxena D., Serle J. B., Shaikh M. A., Shen T. T., Shibuya K., Shin J. I., Silva J. C., Silvester A., Singh J. A., Singhal D., Sitorus R. S., Skiadaresi E., Skirbekk V., Soheili A., Sousa R. A. R. C., Spurlock E. E., Stambolian D., Taddele B. W., Tadesse E. G., Tahhan N., Tareque M. I., Topouzis F., Tran B. X., Travillian R. S., Tsilimbaris M. K., Varma R., Virgili G., Wang Y. X., Wang N., West S. K., Wong T. Y., Zaidi Z., Zewdie K. A., Jonas J. B., Vos T. (2021). Lancet Global Health.

[cit47] Wong W. L., Su X., Li X., Cheung C. M. G., Klein R., Cheng C.-Y., Wong T. Y. (2014). Lancet Global Health.

[cit48] Steinmetz J. D., Bourne R. R. A., Briant P. S., Flaxman S. R., Taylor H. R. B., Jonas J. B., Abdoli A. A., Abrha W. A., Abualhasan A., Abu-Gharbieh E. G., Adal T. G., Afshin A., Ahmadieh H., Alemayehu W., Alemzadeh S. A. S., Alfaar A. S., Alipour V., Androudi S., Arabloo J., Arditi A. B., Aregawi B. B., Arrigo A., Ashbaugh C., Ashrafi E. D., Atnafu D. D., Bagli E. A., Baig A. A. W., Bärnighausen T. W., Battaglia Parodi M., Beheshti M. S., Bhagavathula A. S., Bhardwaj N., Bhardwaj P., Bhattacharyya K., Bijani A., Bikbov M., Bottone M., Braithwaite T. M., Bron A. M., Burugina Nagaraja S. A., Butt Z. A., Caetano Dos Santos F. L. L., Carneiro V. L. J., Casson R. J., Cheng C.-Y. J., Choi J.-Y. J., Chu D.-T., Cicinelli M. V. M., Coelho J. M. G., Congdon N. G. A., Couto R. A. A., Cromwell E. A. M., Dahlawi S. M., Dai X., Dana R., Dandona L., Dandona R. A., Del Monte M. A., Derbew Molla M., Dervenis N. A., Desta A. A. P., Deva J. P., Diaz D., Djalalinia S. E., Ehrlich J. R., Elayedath R. R., Elhabashy H. R. B., Ellwein L. B., Emamian M. H., Eskandarieh S., Farzadfar F. G., Fernandes A. G., Fischer F. S., Friedman D. S. M., Furtado J. M., Gaidhane S., Gazzard G., Gebremichael B., George R., Ghashghaee A., Gilani S. A., Golechha M., Hamidi S. R., Hammond B. R. R., Hartnett M. E. R. K., Hartono R. K., Hashi A. I., Hay S. I., Hayat K., Heidari G., Ho H. C., Holla R., Househ M. J., Huang J. J. E., Ibitoye S. E. M., Ilic I. M. D., Ilic M. D. D., Ingram A. D. N., Irvani S. S. N., Islam S. M. S., Itumalla R., Jayaram S. P., Jha R. P., Kahloun R., Kalhor R., Kandel H., Kasa A. S., Kavetskyy T. A., Kayode G. A. H., Kempen J. H., Khairallah M., Khalilov R. A., Khan E. A. C., Khanna R. C., Khatib M. N. A., Khoja T. A. E., Kim J. E., Kim Y. J., Kim G. R., Kisa S., Kisa A., Kosen S., Koyanagi A., Kucuk Bicer B., Kulkarni V. P., Kurmi O. P., Landires I. C., Lansingh V. C. L., Leasher J. L. E., LeGrand K. E., Leveziel N., Limburg H., Liu X., Madhava Kunjathur S., Maleki S., Manafi N., Mansouri K., McAlinden C. G., Meles G. G. M., Mersha A. M., Michalek I. M. R., Miller T. R., Misra S., Mohammad Y., Mohammadi S. F. A., Mohammed J. A. H., Mokdad A. H., Moni M. A. A., Montasir A. A. R., Morse A. R. F., Mulaw G. F. C., Naderi M., Naderifar H. S., Naidoo K. S., Naimzada M. D., Nangia V., Narasimha Swamy S. M., Naveed D. M., Negash H. L., Nguyen H. L., Nunez-Samudio V. A., Ogbo F. A., Ogundimu K. T., Olagunju A. T. E., Onwujekwe O. E., Otstavnov N. O., Owolabi M. O., Pakshir K., Panda-Jonas S., Parekh U., Park E.-C., Pasovic M., Pawar S., Pesudovs K., Peto T. Q., Pham H. Q., Pinheiro M., Podder V., Rahimi-Movaghar V., Rahman M. H. U. Y., Ramulu P. Y., Rathi P., Rawaf S. L., Rawaf D. L., Rawal L., Reinig N. M., Renzaho A. M., Rezapour A. L., Robin A. L., Rossetti L., Sabour S., Safi S., Sahebkar A., Sahraian M. A. M., Samy A. M., Sathian B., Saya G. K., Saylan M. A., Shaheen A. A. A., Shaikh M. A. T., Shen T. T., Shibuya K. S., Shiferaw W. S., Shigematsu M., Shin J. I., Silva J. C., Silvester A. A., Singh J. A., Singhal D. S., Sitorus R. S., Skiadaresi E. Y., Skryabin V. Y. A., Skryabina A. A., Soheili A. B., Sorrie M. B. A. R. C., Sousa R. A. R. C., Sreeramareddy C. T., Stambolian D. G., Tadesse E. G., Tahhan N. I., Tareque M. I., Topouzis F. X., Tran B. X., Tsegaye G. K., Tsilimbaris M. K., Varma R., Virgili G., Vongpradith A. T., Vu G. T., Wang Y. X., Wang N. H., Weldemariam A. H. K., West S. K. G., Wondmeneh T. G. Y., Wong T. Y., Yaseri M., Yonemoto N., Yu C. S., Zastrozhin M. S., Zhang Z.-J. R., Zimsen S. R., Resnikoff S., Vos T. (2021). Lancet Global Health.

[cit49] Li J. Q., Welchowski T., Schmid M., Mauschitz M. M., Holz F. G., Finger R. P. (2020). Br. J. Ophthalmol..

[cit50] Schmidt-Erfurth U., Chong V., Loewenstein A., Larsen M., Souied E., Schlingemann R., Eldem B., Monés J., Richard G., Bandello F. (2014). Br. J. Ophthalmol..

[cit51] Schmidt-Erfurth U., Garcia-Arumi J., Bandello F., Berg K., Chakravarthy U., Gerendas B. S., Jonas J., Larsen M., Tadayoni R., Loewenstein A. (2017). Ophthalmologica.

[cit52] Wang R., McClard C. K., Laswell S., Mahmoudzadeh R., Salabati M., Ammar M., Vannavong J., Aziz A. A., Ewald A., Calvanese A. V., Lehman E. B., Fried S., Windham V., Strutt A., Saroj N., Khanani A. M., Eichenbaum D. A., Regillo C., Wykoff C. C. (2022). BMJ Open Ophthalmol..

[cit53] McClard C. K., Wang R., Windham V., Munoz J., Gomez S., Fried S., Saroj N., Regillo C., Wykoff C. C., Strutt A. M. (2021). BMJ Open Ophthalmol..

[cit54] Wang R., Liu Y., Xiao W., Yi Q., Jiang M., Guo R., Song L., Li M., Li F., Shi D., Zhao L., Huang W., Zuo X., Mao X. (2023). ACS Appl. Mater. Interfaces.

[cit55] Wang J., Liu Y., Li Y., Dai W., Guo Z., Wang Z., Zhang Q. (2012). Invest. Ophthalmol. Visual Sci..

[cit56] Iwase T., Fu J., Yoshida T., Muramatsu D., Miki A., Hashida N., Lu L., Oveson B., Lima e Silva R., Seidel C., Yang M., Connelly S., Shen J., Han B., Wu M., Semenza G. L., Hanes J., Campochiaro P. A. (2013). J. Controlled Release.

[cit57] Kelly S. J., Halasz K., Smalling R., Sutariya V. (2019). Drug Dev. Ind. Pharm..

[cit58] Farhat W., Yeung V., Kahale F., Parekh M., Cortinas J., Chen L., Ross A. E., Ciolino J. B. (2022). Bioengineering.

[cit59] Boddu S. H. S., Jwala J., Chowdhury M. R., Mitra A. K. (2010). J. Ocul. Pharmacol. Ther..

[cit60] Long K., Yang Y., Lv W., Jiang K., Li Y., Lo A. C. Y., Lam W. C., Zhan C., Wang W. (2021). Adv. Sci..

[cit61] Gao R., Mitra R. N., Zheng M., Wang K., Dahringer J. C., Han Z. (2018). Adv. Funct. Mater..

[cit62] Li M., Bian X., Chen X., Fan N., Zou H., Bao Y., Zhou Y. (2022). Drug Deliv..

[cit63] Hu T., Le Q., Wu Z., Wu W. (2007). J. Pharm. Biomed. Anal..

[cit64] Linko V., Shen B., Tapio K., Toppari J. J., Kostiainen M. A., Tuukkanen S. (2015). Sci. Rep..

[cit65] Stahl E., Martin T. G., Praetorius F., Dietz H. (2014). Angew. Chem., Int. Ed..

[cit66] Seitz I., Ijäs H., Linko V., Kostiainen M. A. (2022). ACS Appl. Mater. Interfaces.

[cit67] Hung A. M., Micheel C. M., Bozano L. D., Osterbur L. W., Wallraff G. M., Cha J. N. (2010). Nat. Nanotechnol..

[cit68] Liang J., Zhang Z., Zhao H., Wan S., Zhai X., Zhou J., Liang R., Deng Q., Wu Y., Lin G. (2018). RSC Adv..

[cit69] Udomprasert A., Wootthichairangsan C., Duangrat R., Chaithongyot S., Zhang Y., Nixon R., Liu W., Wang R., Ponglikitmongkol M., Kangsamaksin T. (2022). ACS Appl. Bio Mater..

[cit70] Zeng Y., Liu J., Yang S., Liu W., Xu L., Wang R. (2018). J. Mater. Chem. B.

[cit71] Li M., Yang G., Zheng Y., Lv J., Zhou W., Zhang H., You F., Wu C., Yang H., Liu Y. (2023). J. Nanobiotechnol..

[cit72] Zhao Y.-X., Shaw A., Zeng X., Benson E., Nyström A. M., Högberg B. (2012). ACS Nano.

[cit73] Hanke M., Tomm E., Grundmeier G., Keller A. (2023). ChemBioChem.

[cit74] Roodhuizen J. A. L., Hendrikx P. J. T. M., Hilbers P. A. J., de Greef T. F. A., Markvoort A. J. (2019). ACS Nano.

[cit75] Bednarz A., Sønderskov S. M., Dong M., Birkedal V. (2023). Nanoscale.

[cit76] Peeters L., Sanders N. N., Braeckmans K., Boussery K., Van de Voorde J., De Smedt S. C., Demeester J. (2005). Invest. Ophthalmol. Visual Sci..

[cit77] Xu T., Yu S., Sun Y., Wu S., Gao D., Wang M., Wang Z., Tian Y., Min Q., Zhu J.-J. (2021). Small.

[cit78] Wang P., Rahman M. A., Zhao Z., Weiss K., Zhang C., Chen Z., Hurwitz S. J., Chen Z. G., Shin D. M., Ke Y. (2018). J. Am. Chem. Soc..

[cit79] Wang Y., Benson E., Fördős F., Lolaico M., Baars I., Fang T., Teixeira A. I., Högberg B. (2021). Adv. Mater..

[cit80] Linko V., Keller A. (2023). Small.

[cit81] Lacroix A., Vengut-Climent E., De Rochambeau D., Sleiman H. F. (2019). ACS Cent. Sci..

[cit82] Kokavec J., Min S. H., Tan M. H., Gilhotra J. S., Newland H. S., Durkin S. R., Grigg J., Casson R. J. (2016). Clin. Exp. Ophthalmol..

[cit83] Sadeghi A., Ruponen M., Puranen J., Cao S., Ridolfo R., Tavakoli S., Toropainen E., Lajunen T., Ranta V.-P., van Hest J., Urtti A. (2022). Int. J. Pharm..

[cit84] Kuo H.-K., Chen Y.-H., Wu P.-C., Wu Y.-C., Huang F., Kuo C.-W., Lo L.-H., Shiea J. (2012). Invest. Ophthalmol. Visual Sci..

[cit85] Pan Z., Zhu H., Zhang Y., Liao Q., Sun Y., Wu E., Wang Y., Shi K., Zhang Y., Chen L., Ye M., Wu W. (2023). Anal. Chem..

[cit86] Mohammadi S. O., LaRocca M. C., Yang C. D., Jessen J., Kenney M. C., Lin K. Y. (2023). J. Clin. Transl. Ophthalmol..

[cit87] Zelada-Guillén G. A., Ríos-Arce J. A., Leyva-Peralta M. A., Flores-Álamo M., Gálvez-Ruíz J. C., Calderón K., Escárcega-Bobadilla M. V. (2023). Chem. Biodivers..

[cit88] Shen Z., Shao J., Dai J., Lin Y., Yang X., Ma J., He Q., Yang B., Yao K., Luo P. (2016). Toxicol. Rep..

[cit89] García-Olaiz G. D., Alcántar-Zavala E., Ochoa-Terán A., Cabrera A., Muñiz-Salazar R., Montes-Ávila J., Salazar-Medina A. J., Alday E., Velazquez C., Medina-Franco J. L., Laniado-Laborín R. (2020). Bioorg. Chem..

[cit90] Lisitsyna E. S. , Klose A., Vuorimaa-Laukkanen E., Ijäs H., Lajunen T., Suhling K., Linko V. and Laaksonen T., *bioRxiv*, 2024, preprint, 10.1101/2024.06.20.599777 PMC12235592 40636881

[cit91] Guo Z., Song H., Tian Y., Xu J., Zhang G., Guo Y., Shen R., Wang D. (2024). Int. J. Nanomed..

[cit92] Mikkilä J., Eskelinen A.-P., Niemelä E. H., Linko V., Frilander M. J., Törmä P., Kostiainen M. A. (2014). Nano Lett..

[cit93] Suen W.-L. L., Chau Y. (2014). J. Pharm. Pharmacol..

[cit94] Green C. M., Mathur D., Medintz I. L. (2020). J. Mater. Chem. B.

[cit95] Wang P., Rahman M. A., Zhao Z., Weiss K., Zhang C., Chen Z., Hurwitz S. J., Chen Z. G., Shin D. M., Ke Y. (2018). J. Am. Chem. Soc..

