# Supplementary material for: Doxorubicin-loaded DNA origami nanostructures: stability in vitreous and their uptake and toxicity in ocular cells
Source: Nanoscale. 2024 Aug 27;16(37):17585–98. doi: 10.1039/d4nr01995d (PMC11372452; doi:10.1039/d4nr01995d)
Supplement: NR-016-D4NR01995D-s001 [file NR-016-D4NR01995D-s001.pdf]

Supplementary Information (SI) for Nanoscale.
This journal is © The Royal Society of Chemistry 2024

Electronic Supplementary Information (ESI)

Doxorubicin-loaded DNA origami nanostructures: Stability in vitreous and their uptake and toxicity in ocular cells

Anna Klose, Zahra Gounani, Heini Ijäs, Tatu Lajunen, Veikko Linko,* and Timo Laaksonen**

A. Klose, Z. Gounani, T. Lajunen, T. Laaksonen
Drug Research Program, Division of Pharmaceutical Biosciences, Faculty of Pharmacy, University of Helsinki, Viikinkaari 5, 00790 Helsinki, Finland

H. Ijäs, V. Linko
Biohybrid Materials, Department of Bioproducts and Biosystems, Aalto University, P.O. Box 16100, 00076 Aalto, Finland

T. Lajunen
School of Pharmacy, University of Eastern Finland, Yliopistonrinne 3, 70210 Kuopio, Finland

V. Linko
Institute of Technology, University of Tartu, Nooruse 1, 50411, Tartu, Estonia

T. Laaksonen
Chemistry and Advanced Materials, Faculty of Engineering and Natural Sciences, Tampere University, Korkeakoulunkatu 8, 33720 Tampere, Finland

E-mail addresses of corresponding authors:

*E-Mail: anna.klose@helsinki.fi

*E-Mail: veikko.pentti.linko@ut.ee

*E-Mail: timo.laaksonen@helsinki.fi

Table of Content

SI1: DNA origami folding, PEG precipitation and TEM sample preparation 3

SI2: 24HB with Atto488-attachments 4

SI3: AGE of folded and purified 60HB and plate 6

SI4: AGE of folded and purified 24HB with and without Atto488 attachment 7

SI5: Processing and analysis of DOX loading into DNA origami 8

SI6: Absolute DOX concentration in DONs-DOX after loading and purification, drug loading, loading efficiency, and DOX every N bp 10

SI7: TEM of 60HB in FOB, MQ and after DOX-loading 11

SI8: TEM of Plate in FOB, MQ and after DOX-loading 12

SI9: Stability of DOX-DONs on AGE (3 vs. 7 weeks) 13

SI10: Stability of DONs and DOX-DONs over 7 weeks at 4 °C (DLS) 14

SI11: Stability of 24HB and 24HB-Atto488 short in cell media at 37 °C for 4 h 15

SI12: Stability of 24HB-Atto488-DOX in cell medium at 37 °C for 24 h 16

SI13: Stability of 24HB-Atto488 in vitreous at 37 °C for 4 h 17

SI14: Stability of 24HB in vitreous at 37 °C (72 h, 7 days) 18

SI15: Uptake of 24HB-Atto488 with LysoTracker™ Deep Red in ARPE-19 (4 h) 19

SI16: Confocal Images: 24HB-Atto488-DOX with LysoTracker™ Deep Red in ARPE-19 (up to 24 h) 20

SI17: Confocal Images: 24HB-Atto488 with CellMask™ Deep Red in ARPE-19 (24 h) 21

SI18: Confocal Images: 24HB-Atto488 with Cell Mask™ in ARPE-19 (24 h, Z-stack/3D) 22

SI19: Confocal Images: 24HB-Atto488-DOX with Cell Mask Deep Red™ in ARPE-19 (24 h, Z-stack/3D) 23

SI20: Confocal Images: Atto488-strands with Cell Mask™ Deep Red in ARPE-19 (24 h, Z-stack/3D) 24

References 25

SI1: DNA origami folding, PEG precipitation and TEM sample preparation

DNA origami folding. 60HB was folded as previously reported by Linko et al.[1] In short, the scaffold strand p7249 (Tilibit) at a final concentration of 20 nM was added to 10× excess of staple strands (Integrated DNA technologies) in a 1× folding buffer (FOB), comprising of 1× TAE buffer (40 mM Tris, 20 mM acetic acid, 1 mM EDTA), 20 mM $MgCl_2$, 5 mM NaCl). The folding reaction was annealed as follows: from 65 °C to 59 °C at -1 °C/15 min, from 59 °C to 40 °C at -0.25 °C/45 min, and final storage at 12 °C. **24HB** was folded using a p7560 (Tilibit) scaffold (staples from Integrated DNA technologies) in a 1× FOB comprising of 1× TAE buffer and 17.5 mM $MgCl_2$.[2] The annealing program was the same as for the 60HB. The **Plate** was annealed from the p7249 scaffold with 7.5× excess of staple strands (Integrated DNA technologies) in the same 1× FOB as 60HB.[3] The folding reaction was completed overnight (by cooling from 75 °C to 70 °C in steps of 0.2 °C/8 s, from 70 °C to 60 °C in steps of 0.1 °C/8 s, from 60 °C to 27 °C in steps of 0.1 °C/2 min and final storage at 12 °C).

Polyethylene glycol (PEG) precipitation. To remove excess staple strands, the DON solution was diluted to approximately 5 nM concentration and mixed 1:1 (v/v) with PEG precipitation buffer, consisting of 15% (w/v) PEG 8000, 1× TAE, and 505 mM NaCl.[4] After centrifuging (14 000 g, 30 min), the supernatant was removed and the DON pellet redissolved in 1× FOB on a shaker overnight (30 °C, 600 rpm). The DON concentration was determined via absorption measurement, and further characterizations were performed (as described in Methods and Materials in the main text).

TEM sample preparation. DONs were diluted to 2-5 nM and deposited on plasma-cleaned Formvar carbon-coated copper grids (FCF-400-CU, Electron Microscopy Sciences), similarly as reported by Castro et al.[5] After 3-6 min of incubation, the liquid was blotted away and the sample was negatively stained with a 2% (w/v) uranyl formate solution (pH adjusted with 25 mM NaOH). For that, the grid was immersed in a 5 µl droplet and immediately blotted. After dipping the grids in a 20 µl droplet, the incubation continued for 45 s before final blotting. The grids were then left for drying for at least 30 min before imaging. The samples were imaged using FEI Tecnai 12 Bio-Twin electron microscope (120 kV acceleration voltage) at the OtaNano Nanomicroscopy Center at Aalto University or with a Hitachi HT7800 Transmission Electron Microscope (100 kV acceleration voltage) at the Electron Microscopy Unit at University of Helsinki.

SI2: 24HB with Atto488-attachments

In initial testing, short Atto488-strands (17 nucleotides, Integrated DNA technologies) were incorporated into the 24HB structure (24HB-Atto488 short, sequences described by Seitz et al.),[6] but a lot of fluorophores dissociated from 24HB upon incubation in cell media at 37 °C despite additional Mg^{2+} supplementation (ESI Fig. SI12). To investigate if using longer strands and a different sequence could increase melting temperatures of Atto488-strands and thus reduce the Atto488 loss in cell media, the staple extensions for Atto488-strands as well as the Atto488-strands were elongated by additional six bases (23 nucleotides, **bold**) and by replacing one adenine for guanine (underlined, resulting in an additional hydrogen bond with cytosine compared to the adenine-thymine pairing). Since the thymine adjacent to the fluorophore on the Atto488-strands was left unpaired, it corresponded to 16 bp-, or 22 bp-bindings with the extended staple strands, respectively.

Atto488 short (17 nt): /5Atto488N/TGG GAA AGG AGA AAA AA (described by Seitz et al.)[6]

Atto488 (23 nt): /5ATTO488N/TGG GAG AGG **AGG AGG** AGA AAA AA

The Atto488-strands were annealed to the folded 24HB with overhanging extensions at 10× molar excess per attachment site (by cooling 40 °C to 20 °C in steps of -0.1 °C/40 s, holding at 20 °C). Excess Atto488-strands were removed by PEG-precipitation and the final concentration was estimated via absorbance at 260 nm (as described in the Methods in the main text). Ɛ was estimated to be 1.076 × 10^8 M/cm for 24HB-Atto488 short, and 1.104 × 10^8 M/cm for 24HB-Atto488 based on the number of nonhybridized and hybridized nucleotides in the structure.[7]

The theoretical melting temperatures for the Atto488-strands were estimated using the IDT OligoAnalyzer™ Tool for different ionic conditions (ESI Tab. SI2a), assuming that at 2 nM DON concentration each structure carries all 24 Atto488 tags.

Tab. SI2a Estimated melting temperatures of Atto488 strands for different ionic parameters. Condition 1 mimicked cell media without, and condition 2 with additional Mg^{2+} supplementation to 3 mM concentration. Condition 3 considered the Mg^{2+} concentration in folding buffer.

Conditions		$T_{melting}$ [°C] for **Atto488 short**	$T_{melting}$ [°C] for **Atto488**
1	48 nM oligonucleotide concentration, 135 mM NaCl, 0.7 mM Mg^{2+}	49.8	62.6
2	48 nM oligonucleotide concentration, 135 mM NaCl, 3 mM Mg^{2+}	53.1	64.7
3	48 nM oligonucleotide concentration, 17.5 mM Mg^{2+}	54.9	65.5

The design and sequences for 24HB have been previously reported by Ijäs et al.[2] For 24HB-Atto488 short, 24 staple strands (r1-r23, l0-l22) were replaced with staples with overhangs to attach the Atto488-strands, reported by Seitz et al.[6] For 24HB-Atto488, those same 24 staple strands were replaced by staples with elongated extensions as shown in the Table SI2b (ESI). The poly-T extensions at the 5'-end were shortened by one thymine for l2, by two for r5, r11, r17 and r19, and for l10 completely removed.

Tab. SI2b Elongated staple strand sequences for attachment of Atto488-strands to 24HB (for 24HB-Atto488). The sequence complementary to the scaffold is shown in capital letters, while overhanging sequences are indicated by lowercase letters. The overhang to attach the Atto488-strand is italicized.

#	Sequence
r1	tttttttGCAAGGATAAAACAATTCTGC*ttttttctcctcctcctctccc*
r3	tttttttAAGCTAAATCGGAATAACCTG*ttttttctcctcctcctctccc*
r5	tttttAGCATTAACATCCAATTTCTACTAATAGTAGT*ttttttctcctcctcctctccc*
r7	tttttttTCATTGCCTCCTCAGAGCATA*ttttttctcctcctcctctccc*
r9	tttttttATAAATTAACTTTATTTCAAC*ttttttctcctcctcctctccc*
r11	tttttAAGGGTGAGAAAGGCCGTAGGTAAAGATTCAA*ttttttctcctcctcctctccc*
r13	tttttttAGGTCACGTTGGTTCTAGCTG*ttttttctcctcctcctctccc*
r15	ttttttttTTAAATGTGAGCGCTATCAGG*ttttttctcctcctcctctccc*
r17	tttttTCATTTTTTAACCAATTTTTTGTTAAATCAGC*ttttttctcctcctcctctccc*
r19	tttttAAATTGTAAACGTTAAGTATAAGCAAATATTT*ttttttctcctcctcctctccc*
r21	GAAGATTTATTTTGCATTAAAAGGAACGTAGCCAGCTTTCATCAACA*ttttttctcctcctcctctccc*
r23	TACAAAGGAGTAACGGATTGACCGTAATGGGAT*ttttttctcctcctcctctccc*
l0	CCAACGCTCCCTTAAAGAGTCCACTATTAAAGA*ttttttctcctcctcctctccc*
l2	ttttttCCGCCTGGCCCTCTGTTTGATGGTGGTTCCG*ttttttctcctcctcctctccc*
l4	tttttttGCGAACTGATAGGATTGCCCTTCA*ttttttctcctcctcctctccc*
l6	tttttttAAATCGGCAAAAGCGGGGAGA*ttttttctcctcctcctctccc*
l8	tttttttACGTGGACTCCATTAATTGCG*ttttttctcctcctcctctccc*
l10	AGCCAGGGTGGATGTTAAGCTTTACCGAGCTCACAATTCCAC*ttttttctcctcctcctctccc*
l12	tttttttTTGCGCTCAGATAAAGACGGA*ttttttctcctcctcctctccc*
l14	tttttttGGCGGTTTGGCATTTCACATA*ttttttctcctcctcctctccc*
l16	CAGTGCCCTTCTAATCCTTAGCCAAAATGGAGTGACTCTATGATACC*ttttttctcctcctcctctccc*
l18	CTGCCATGGCTATTAGTCTTTAATGC*ttttttctcctcctcctctccc*
l20	tttttttAATCATTTCTCCTTGTCAACC*ttttttctcctcctcctctccc*
l22	tttttttGGATCCCCGGGTCTCAGGAGA*ttttttctcctcctcctctccc*

SI3: AGE of folded and purified 60HB and plate

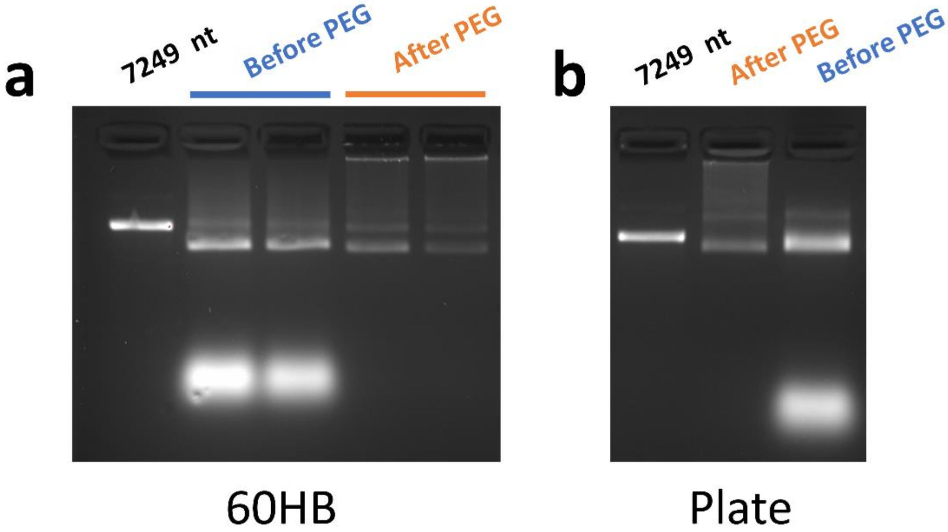

Fig. SI3 Folded 60HB (a) and Plate (b) before and after PEG purification. The folded structures have higher electrophoretic mobility than the scaffold strand (7249 nt) used for their folding. Excess staples are visible as a bright leading band and have been successfully removed after PEG precipitation. (2% agarose gel with 0.46 µg/mL ethidium bromide, 90 V, 50 min, cropped gel images).

SI4: AGE of folded and purified 24HB with and without Atto488 attachment

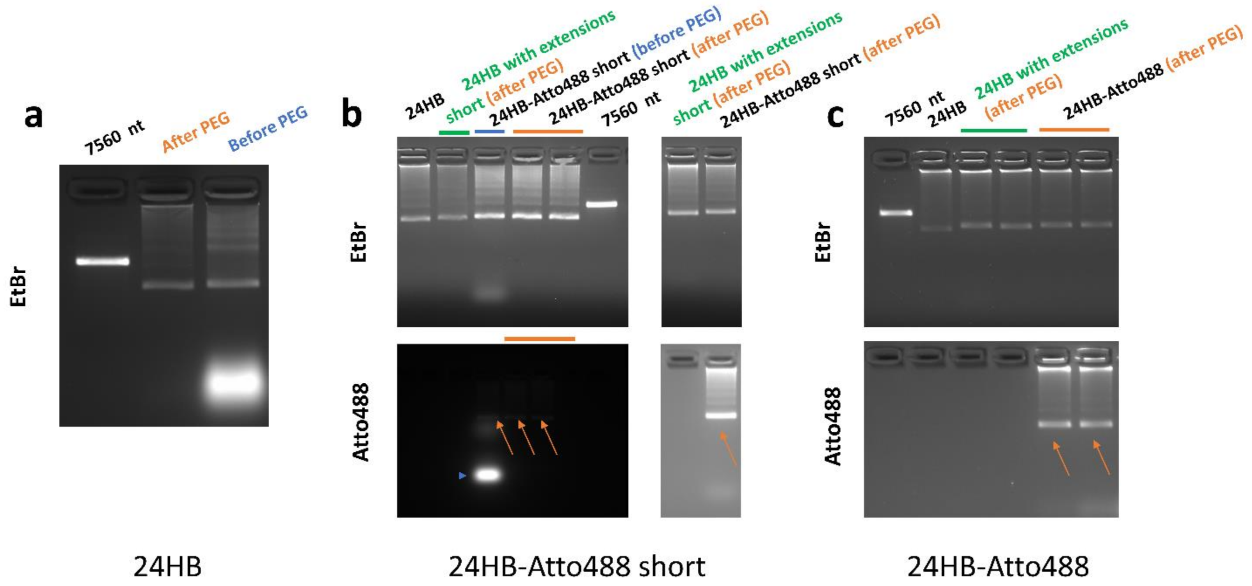

Fig. SI4 Folded 24HB, annealed 24HB-Atto488 short and 24HB-Atto488. a. Folded 24HB has a higher electrophoretic mobility than its scaffold strand (7560 nt). Excess staples are visible as a bright leading band and have been successfully removed after PEG purification. **b.** Compared to 24HB, the band of 24HB with short extensions (after PEG) is slightly shifted upwards, indicative of slower electrophoretic mobility due to its weight increase. A similar band is visible for annealed 24HB-Atto488 short before and after purification. In the Atto488 channel, the excess Atto488 strands (blue arrow) before PEG-purification appear very bright compared to the bands for 24HB-Atto488 short (orange arrow). By removing, 24HB-Atto488 (short) samples before purification from the gel, the brightness in the Atto488 channel is adjusted to the band intensity of 24HB-Atto488 short and becomes clearly visible, showing successful fluorophore attachment and PEG-purification. The scaffold strand (7560 nt) is shown as a reference. **c.** 24HB-Atto488 (attached via 22 nt staple strand extensions) can be annealed and purified successfully with the same protocol as for 24HB-Atto488 short. (2% agarose gel with 0.46 µg/mL ethidium bromide, 90 V, 50 min, cropped gel images).

SI5: Processing and analysis of DOX loading into DNA origami

DOX loading solutions contained 2 nM of DONs and 2.5 µM, 5 µM, or 15 µM as the initial DOX concentration. As references, solutions of similar composition of plain DONs, and free DOX at corresponding concentrations were prepared. After one hour of incubation at room temperature, their absorbance spectra (240-650 nm) were recorded in a quartz cuvette (*l* = 1 cm, Varian Cary 50 UV-Vis Spectrophotometer), before purifying the DOX-loaded DONs via spin-filtration (as described in Methods of the main text).

After purification, the absorbance spectra of the solutions were measured again (Examples in ESI Fig. SI5a-c). Using the three free DOX reference samples (2.5 µM, 5 µM, 15 µM DOX), a linear calibration curve was fitted for their absorbance at 543 nm (A543, isosbestic point), and A260 nm (A260) according to the DOX-concentration [µM]. The absorption at 543 nm is solely due to DOX, but at 260 nm, both DOX and DONs contribute to the overall absorbance. Using the calibration curve-A543, the DOX concentration [µM] in DOX-DONs after purification was determined. To determine later the concentration of DONs after purification, the calibration curve-A260 was used to estimate the absorbance of free DOX at 260 nm ($A260_{DOX}$). For each DOX concentration, the $A260_{DOX}$ was subtracted from the corresponding $A260_{total}$ of the DONs-DOX sample (before purification) that was loaded with the same amount of DOX. The remaining $A260_{DNA}$ was attributed to the DONs in the sample and was used to determine its concentration c in the sample (according to Lambert-Beer law $A_{260} = \mathcal{E}\, c\, l$, with a pathlength *l* = 1 cm, and a molar extinction coefficient $\mathcal{E}$ depending on the DONs as mentioned in Methods in the main text).

The DON concentration before purification is known and was chosen to be 2 nM in all experiments (as measured and correctly determined for the plain DONs reference). Depending on the DOX-concentration in the sample, however, the $A260_{DNA}$ would correspond to DONs concentrations below 2 nM even before purification, hence, was increasingly underestimated the more DOX present in the sample (an example is shown in ESI Fig. SI5d). Since DOX contributed to the $A260_{total}$ and a loss of DONs-DOX in the purification process was expected, the remaining DONs concentration after purification needed to be approximated. To take this into account, we fitted an exponential trendline through the percentage of DON concentration vs. DOX concentration in the DONs-DOX samples before purification (corresponding plain DONs acted as a reference, intercept was set at 100%, ESI Fig. SI5d). This trendline was used to determine and correct for the underestimation of DONs concentration after purification (taking into account the DOX left in the sample) and to determine final drug loading (ESI Fig. **SI6**).

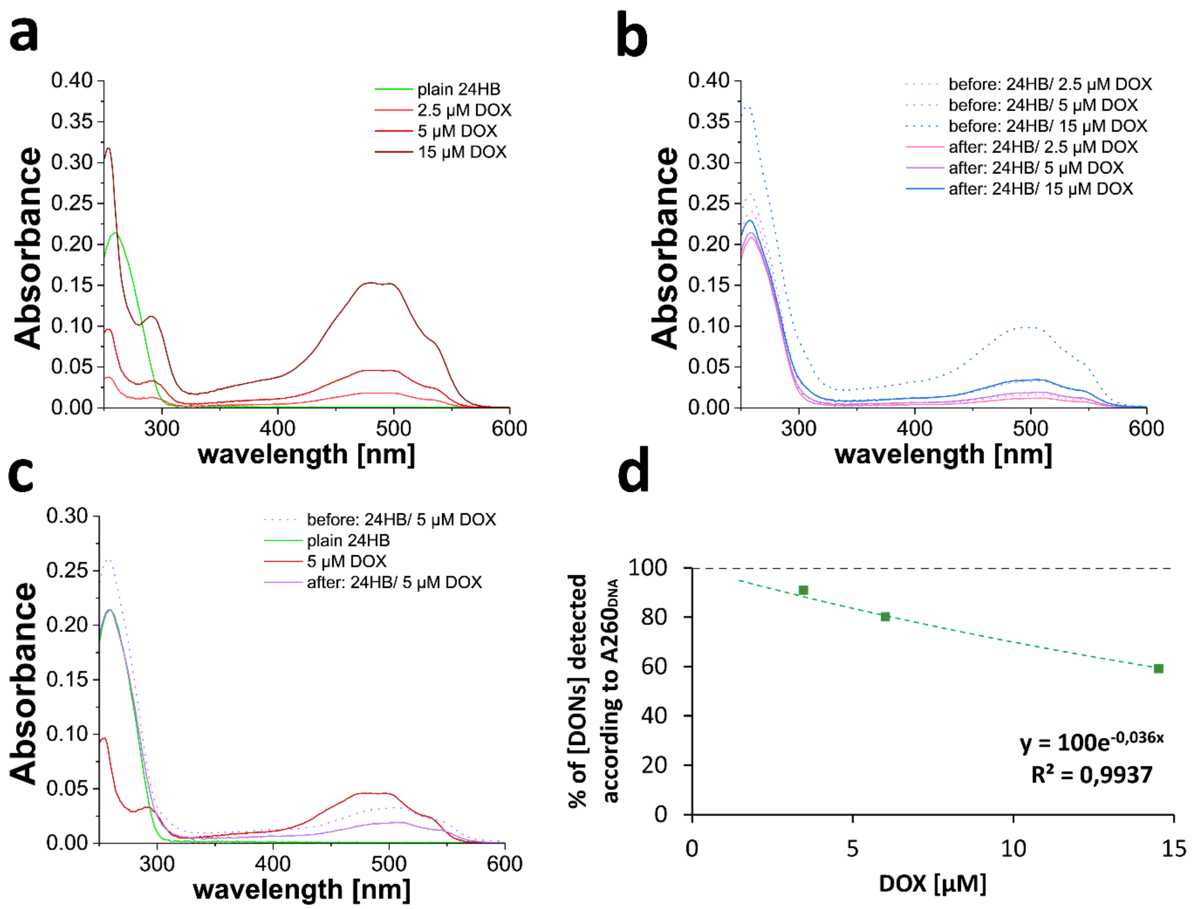

Fig. SI5 Absorbance spectra of DOX, 24HB and 24HB-DOX, and its underestimation of the DNA origami concentration. a. Plain 24HB (green) showed a prominent absorption peak at 260 nm, while free DOX (2.5-15 µM, red) absorbed strongly between 450-550 nm and little at 260 nm. **b.** Spin-filtration of DOX-loaded 24HB before purification (dotted line) reduced the absorption of DOX-loaded 24HB after purification (solid line) between 450-550 nm, meaning DOX was removed in the purification process. The effect was more pronounced for higher DOX loading concentration like 15 µM (blue) and 5 µM (purple), compared to 2.5 µM (pink). **c.** Comparing the A260 of plain 24HB (green) with 24HB/5 µM DOX before purification (dotted line, purple) and after purification (solid line, purple), revealed that DOX (red) contributes to A260, explaining the higher A260 for 24HB/5 µM DOX before purification compared to plain 24HB. To estimate the DONs concentration in 24HB/5 µM DOX after purification, the reduction of DOX in the sample and its remaining contribution to A260 needed to be considered. **d.** DONs concentration in 24HB-DOX before purification, as percentage detected according to $A260_{DNA}$ (compared to plain 24HB) was plotted and fitted in dependence of DOX [µM] in the sample. The expected DONs concentration of 100% (2 nM) was shown with the black dashed line. Using the trendline and equation, the DONs concentrations after purification (based on the remaining DOX concentration in the sample) could be corrected.

SI6: Absolute DOX concentration in DONs-DOX after loading and purification, drug loading, loading efficiency, and DOX every N bp

The drug loadings and loading efficiencies were calculated according to the ESI Equations SI Eq 1 and 2, based on the amounts of total DOX and DONs used and recovered for these experiments, determined via UV/Vis absorbance measurements.

$$\boldsymbol{Drug\ loading} = \frac{\boldsymbol{Mass\ of\ loaded\ DOX}}{\boldsymbol{Mass\ of\ DNA\ origami{+}loaded\ DOX}}\ \mathbf{x\ 100\%} \qquad \textbf{(SI Eq 1)}$$

$$\boldsymbol{Loading\ efficiency} = \frac{\boldsymbol{Amount\ of\ DOX\ loaded}}{\boldsymbol{Amount\ of\ total\ DOX\ used}} \times \mathbf{100\%} \qquad \textbf{(SI Eq 2)}$$

Tab. SI6 DOX loaded into DONs. Mean ± s.d., n = 3.

	DOX [µM] used for loading	DOX [µM] after loading and purification	Drug loading (m/m%) ± StDev	Loading efficiency (%) ± StDev	DOX every N basepairs ± StDev
**24HB**	2.5	2.3 ± 0.4	12.9 ± 1.7	83.6 ± 5.2	6.0 ± 0.8
	5	3.2 ± 0.6	17.8 ±2.2	63.8 ± 2.4	4.1 ± 0.6
	15	5.5 ± 0.5	29.8 ± 1.2	40.6 ± 2.4	2.1 ± 0.1
**Plate**	2.5	2.4 ± 0.2	14.0 ± 1.2	76.3 ± 3.8	5.2 ± 0.5
	5	3.1 ± 0.4	18.8 ± 1.8	61.4 ± 4.4	3.6 ± 0.4
	15	4.8 ± 0.7	28.1 ± 2.1	35.8 ± 4.7	2.2 ± 0.2
**60HB**	2.5	2.5 ± 0.4	16.2 ± 1.4	84.4 ± 17.2	3.9 ± 0.4
	5	2.7 ±0.2	20.1 ± 1.4	56.1 ± 1.9	3.0 ± 0.3
	15	4.8 ± 0.5	33.5 ± 1.3	34.8 ± 2.2	1.5 ± 0.1

For all tested DONs, increasing the DOX loading concentration led to higher drug loadings and higher packing density of DOX into the DONs, but reduced the loading efficiency. The drug loading differed little between the tested DONs, with 60HB having a slightly higher drug carrying capacity. Above the 5 µM DOX loading concentration, the purified DONs loaded so many DOX molecules, equaling to one DOX molecule every 2 or less base pairs. As described by Ijäs *et al.*, that is beyond the realistic intercalation capacity of the respective DONs and indicates another binding mechanism/aggregation besides intercalation.[2,8]

SI7: TEM of 60HB in FOB, MQ and after DOX-loading

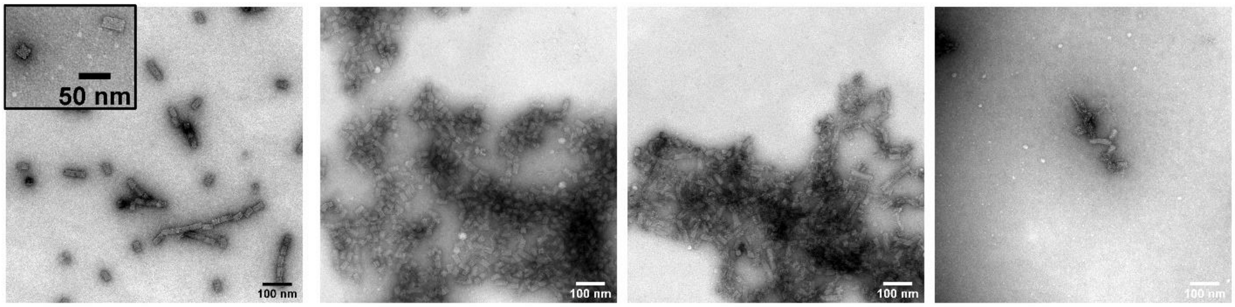

FOB H_2O (old) 60HB/15 µM DOX (new) 60HB/15 µM DOX (old)

Fig. SI7 Transmission Electron Microscopy images of 60HB and 60HB-DOX. 60HB was folded and stable in 1× FOB (Dimensions: 20 x 20 x 31 nm). After spin-filtration, 60HB also retained its shape and integrity in deionized water (H_2O) for 6 weeks (old) at 4 °C. Similarly, purified 60HB loaded with 15 µM DOX appeared in majority stable. Few unraveled structures were visible after 1 day (new) and 6 weeks (old) after storage at 4 °C. Areas with several well-defined structures and good deposition from the respective grids were selected here. (Negative staining with 2% uranyl formate, scalebar: 100 nm (inset: 50 nm, cropped with Fiji-ImageJ))

SI8: TEM of Plate in FOB, MQ and after DOX-loading

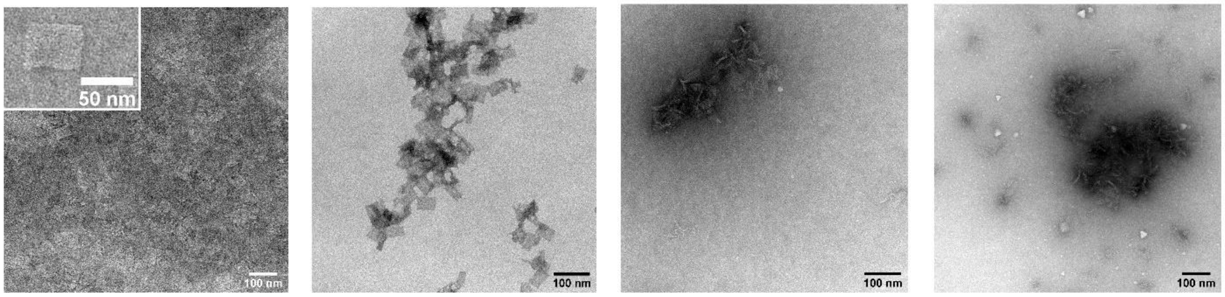

FOB | H_2O (old) | H_2O (old) | Plate/15 µM DOX (old)

Fig. SI8 Transmission Electron Microscopy images of Plate and Plate-DOX. The plate was folded and stable in 1× FOB (Dimensions: 40 x 55 x 6 nm) but due to its thinness, was hard to image with sufficient contrast. After 8 weeks (old) at 4 °C, many plates in deionized water (H_2O) appeared intact, however, some did not deposit themselves flat on the grid, but seemed bent or even standing up on their narrow side, as previously described by Julin et al.[3] Purified Plate-DOX (that was loaded with 15 µM DOX) was also standing up on its side. The shape could hence, not be definitively confirmed from this angle, however, no unravelling was visible, indicating that the DONs-DOX structures mostly remained integer units, as also supported by gel electrophoresis (Fig. 1 in main text). Areas with several well-defined structures and good deposition from the respective grids were selected here. (Negative staining with 2% uranyl formate, scalebar: 100 nm (inset: 50 nm, cropped with Fiji-ImageJ))

SI9: Stability of DOX-DONs on AGE (3 vs. 7 weeks)

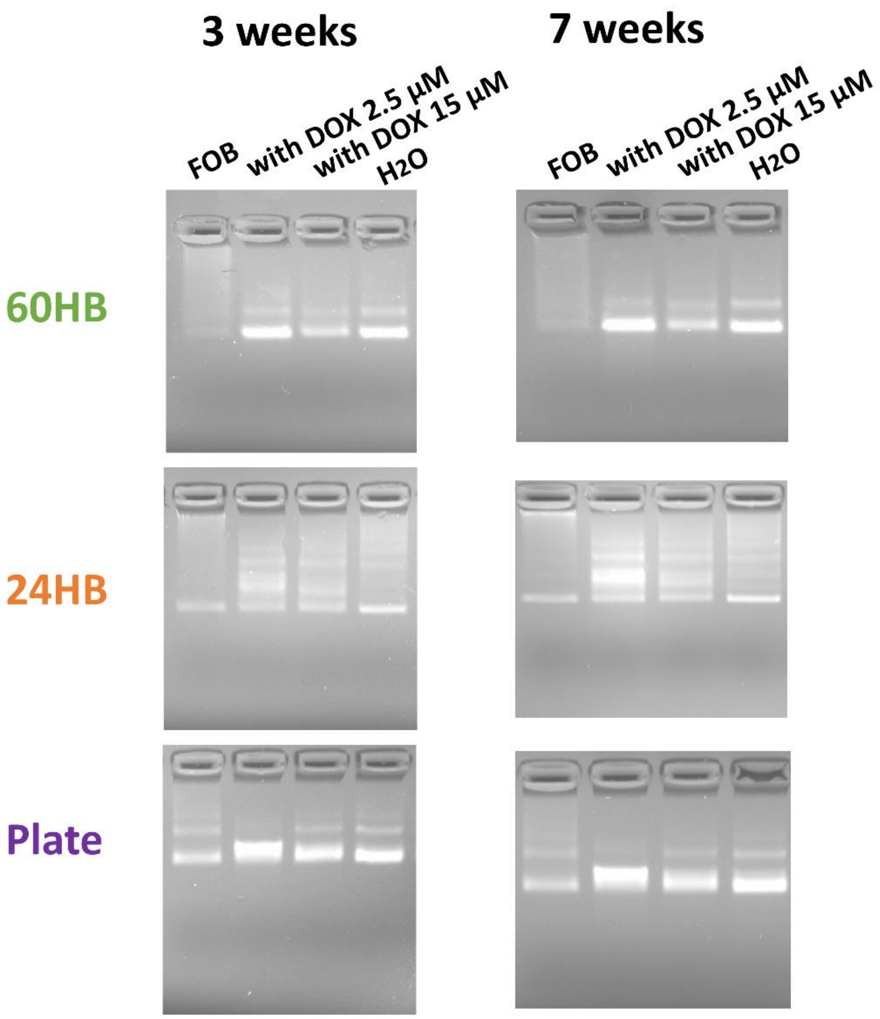

Fig. SI9 Comparison of DONs with and without DOX after 3 and 7 weeks of storage at 4 °C via gel electrophoresis. For all DONs, the DOX-loaded structures (with initial DOX loading concentrations of 2.5 µM and 15 µM) showed similar electrophoretic mobility as the DONs references in folding buffer (FOB) or deionized water (H_2O). While some band intensities might have slightly shifted over time, all the leading bands remained visible, and similar over 7 weeks, indicating that most of the DONs remained stable. The plate in water showed already in week 3 a wider leading band, perhaps hinting at some minor aggregation (2% agarose gel with 0.46 µg/mL ethidium bromide, 90 V, 50 min, cropped gel images).

SI10: Stability of DONs and DOX-DONs over 7 weeks at 4 °C (DLS)

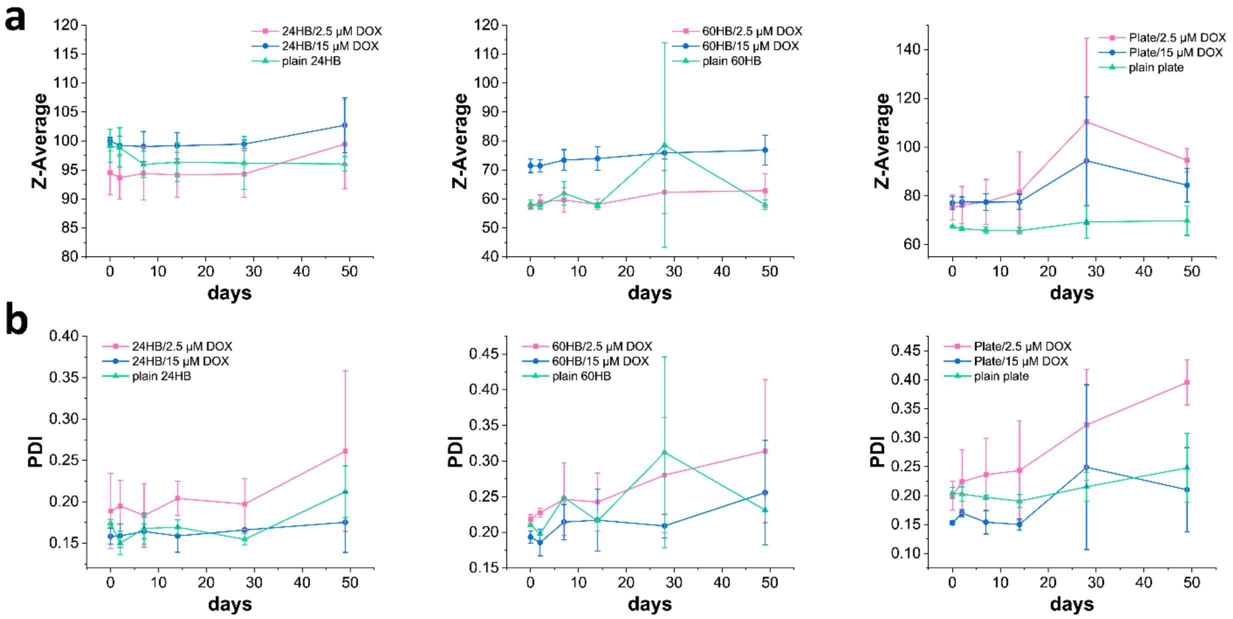

Fig. SI10 Stability of DONs and DOX-DONs over 7 weeks at 4 °C via Dynamic Light Scattering (DLS) measurements. DOX-DONs were loaded with 2.5 µM or 15 µM DOX and purified before storage at 4 °C. DLS size estimations are only reliable for spherical particles, which is not applicable for these DONs used in our study. However, DLS was used to monitor for major aggregation and instability events in the samples during the storage period. **a.** The intensity-weighed Z-Average is very sensitive to an increase if bigger particles and aggregates were present. No larger increase in Z-Average was detectable for any of the investigated samples, indicating that the DONs were mostly stable. Only some stronger fluctuations for plain 60HB and DOX-loaded plate were observable after 4 weeks, that seemed to recover again. **b.** This would also be supported by the corresponding rise in polydispersity index (PDI) of the sample after 4 weeks, indicating that the samples' size distribution became more heterogenous. The PDI for 60HB was on average a bit higher than for the other structures, that have poly-T extensions to reduce aggregation and blunt-end stacking of the DONs. That could mean that 60HB aggregates more easily. Since all DON showed stronger polydispersity after 7 weeks, there is also the possibility that redispersing aggregates became more difficult over time, while the DONs itself remained intact.

SI11: Stability of 24HB and 24HB-Atto488 short in cell media at 37 °C for 4 h

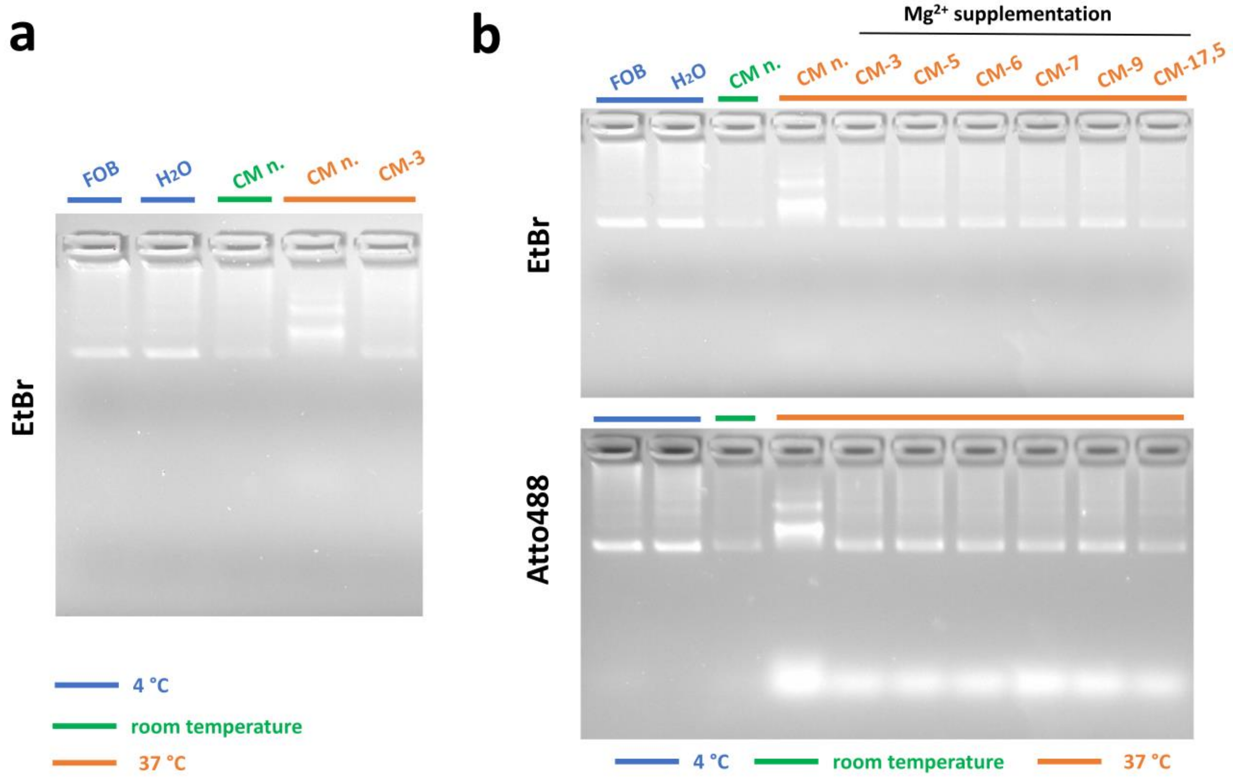

Fig. SI11 Stability of 24HB and 24HB-Atto488 short in cell media (CM) at 37 °C after 4 h via gel electrophoresis. 24HB and 24HB-Atto488 short respectively, served in folding buffer (FOB) and deionized water (H_2O) at 4 °C (blue) as reference and were compared to 24HB(-Atto488 short) incubated in CM-x, supplemented with different amounts of Mg^{2+} (n. =no supplementation, x = Mg^{2+} concentration in mM), at room temperature (green) or at 37 °C (orange). Samples comprised of 90% (v/v) CM-x and 10% (v/v) 24HB/H_2O. All samples were adjusted to the same Mg^{2+} concentration for running the gel. **a.** The shift in the leading band revealed the instability of 24HB in CM n. at 37 °C, that could be counteracted by Mg^{2+} addition (CM-3). At room temperature, 24HB in CM n. remained stable. **b.** The same was observed for 24HB-Atto488 short incubated in CM-x. In the Atto488-channel, the loss of Atto488 in CM n. at 37 °C became apparent, and was slightly reduced by supplementation of Mg^{2+}. However, the signal of free Atto488 was still quite prominent and persistent even with higher concentrations of Mg^{2+}. To reduce the Atto488 loss, the staple strands with extensions for Atto488 attachment and the corresponding Atto488-strands were elongated to increase the melting temperature for further investigations (as described in SI2 and in the main text). (2% agarose gel with 0.46 µg/mL ethidium bromide, 90 V, 50 min, cropped gel images).

SI12: Stability of 24HB-Atto488-DOX in cell medium at 37 °C for 24 h

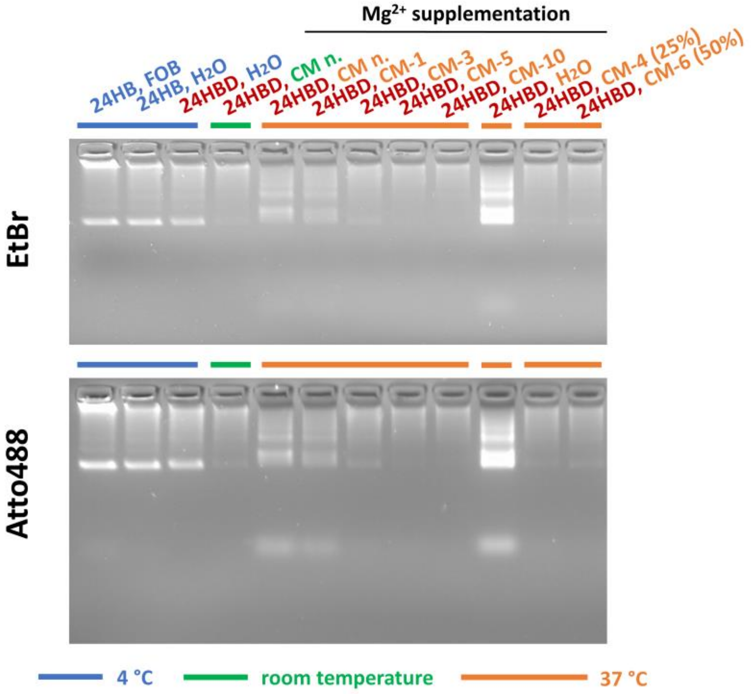

Fig. SI12 Stability of 24HB-Atto488-DOX in cell media (CM) at 37 °C after 24 h via gel electrophoresis. 24HB-Atto488 (here denoted as 24HB) and 24HB-Atto488-DOX (24HBD) in folding buffer (FOB) and in deionized water (H_2O) at 4 °C (blue) served as reference. 24HBD was incubated in water and CM-x at room temperature (green) and at 37 °C (orange), supplemented with different amounts of Mg^{2+} (n. = no supplementation, x = Mg^{2+} concentration in mM). Samples comprised of 80% (v/v) CM-x and 20% (v/v) 24HBD/H_2O, except for 24HBD, CM-4 (25%) and CM-6 (50%), where 24HBD/H_2O made up 25% or 50% (v/v), respectively. All samples were adjusted to the same Mg^{2+} concentration before running the gel. At 37 °C, the shift and widening of the leading band for 24HBD in CM n. and CM-1 indicated that 24HBD was not completely stable, releasing free Atto488-strands from its structure (Atto488-channel). 24HBD appeared stable upon more Mg^{2+}-supplementation (starting from CM-3) with reduced Atto488 loss. 24HBD appeared also stable (no increase in Atto488 loss) in CM-5, CM-10, CM-4 (25%) and CM-6 (50%), though the bands were very faint, indicating lower DOX-DONs concentration perhaps due to some aggregation either in the gel well or in the incubated sample itself. At 37 °C, 24HBD in water showed in comparison to 24HB-Atto488 without DOX (Fig. 1 in main text) some instability resulting in Atto488 loss, hinting at a destabilizing effect of DOX. Overall, DOX-loaded 24HB-Atto488 were stable in CM upon sufficient Mg^{2+}-supplementation and were used for further confocal imaging studies. (2% agarose gel with 0.46 µg/mL ethidium bromide, 90 V, 50 min, cropped gel images).

SI13: Stability of 24HB-Atto488 in vitreous at 37 °C for 4 h

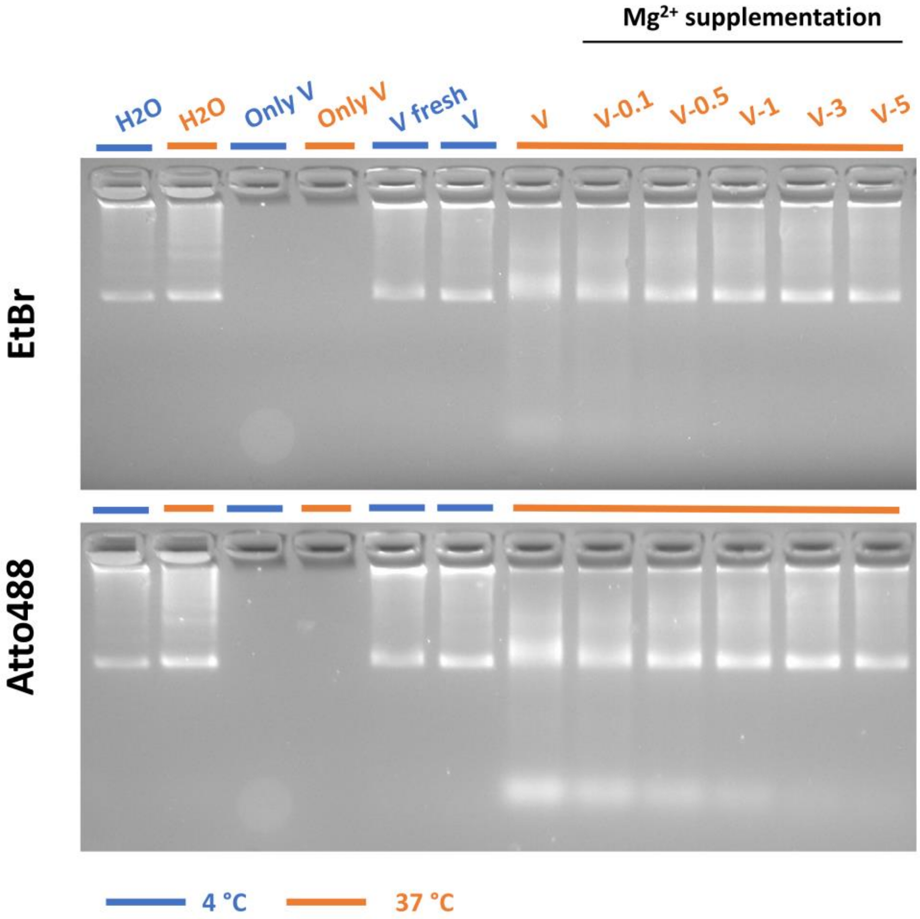

Fig. SI13 Stability of 24HB-Atto488 in porcine vitreous (V) after 4 h at 37 °C via gel electrophoresis. 24HB-Atto488 in deionized water (H_2O), and plain vitreous (Only V) at 4 °C (blue) and 37 °C (orange) served as references. 24HB-Atto488 was incubated with V-x, supplemented with different amounts of Mg^{2+} (V= no supplementation, x= Mg^{2+} concentration in mM added to the incubation reaction). Samples comprised of 70% (v/v) V-x and 30% (v/v) 24HB-Atto488/H_2O and were diluted before loading onto the gel. V fresh was loaded onto the gel without any incubation with 24HB-Atto488. All samples were adjusted to the same Mg^{2+} concentration before running the gel. Only V did not give any background signal in the EtBr or Atto488 channel. While 24HB-Atto488 appeared stable in V without additional Mg^{2+} at 4 °C, at 37 °C, some smearing of the leading band and Atto488 loss was observable. Upon Mg^{2+} addition of minimum 3 mM, 24HB-Atto488 was stabilized and the Atto488 loss reduced. (2% agarose gel with 0.46 µg/mL ethidium bromide, 90 V, 50 min, cropped gel images).

SI14: Stability of 24HB in vitreous at 37 °C (72 h, 7 days)

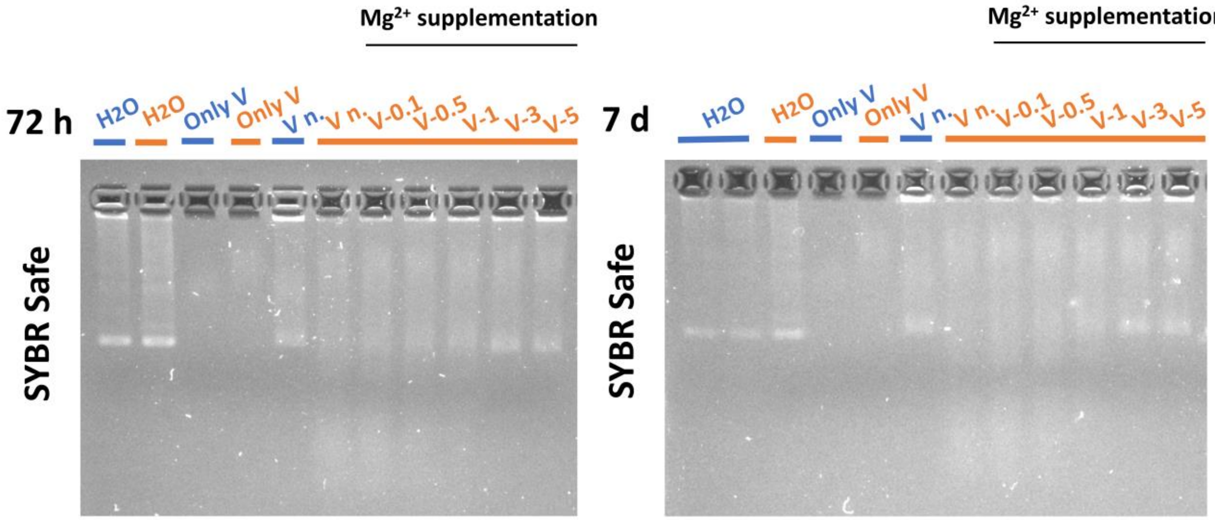

Fig. SI14 Stability of 24HB in porcine vitreous (V) at 37 °C after 72 h and 7 days via gel electrophoresis. 24HB in deionized water (H_2O) and plain vitreous (Only V) at 4 °C (blue) and 37 °C (orange) served as references for 24HB incubated with V-x, supplemented with different amounts of Mg^{2+} (n.=no supplementation, x = Mg^{2+} concentration in mM added to incubation reaction). Samples comprised of 77% (v/v) V-x and 23% (v/v) 24HB-Atto488/H_2O and were diluted before loading onto the gel. All samples were adjusted to the same Mg^{2+} content for running the gel. Only V did not give any background signal. Smearing of the leading band for 24HB was observable after 72 h in V n. without additional Mg^{2+} at 37 °C. Thus, addition of minimum 3 mM Mg^{2+} was necessary to stabilize 24HB, and to maintain its stability for 7 days. (2% agarose gel with 1x SYBR Safe, 90 V, 50 min, cropped gel images).

SI15: Uptake of 24HB-Atto488 with LysoTracker^{TM} Deep Red in ARPE-19 (4 h)

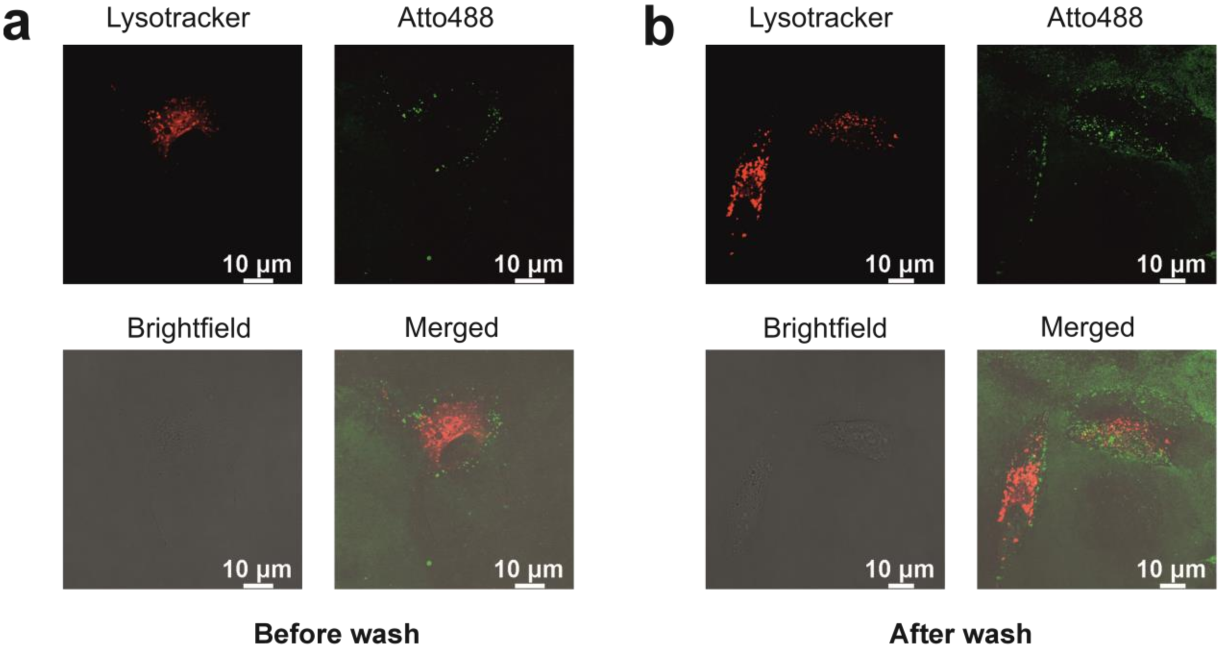

Fig. SI15 Live confocal images of ARPE-19 treated with 24HB-Atto488 (5.3 nM) for 4 h (before and after washing). a. Few 24HB-Atto488 (green) seemed to have entered the cells, many appeared to be located outside of and around the cell. **b.** Washing could not remove 24HB-Atto488 attached outside of the cells. The lysosomes of ARPE-19 cells were stained with LysoTracker^{TM} Deep Red (red), but no co-localization with 24HB-Atto488 was visible.

SI16: Confocal Images: 24HB-Atto488-DOX with LysoTracker^{TM} Deep Red in ARPE-19 (up to 24 h)

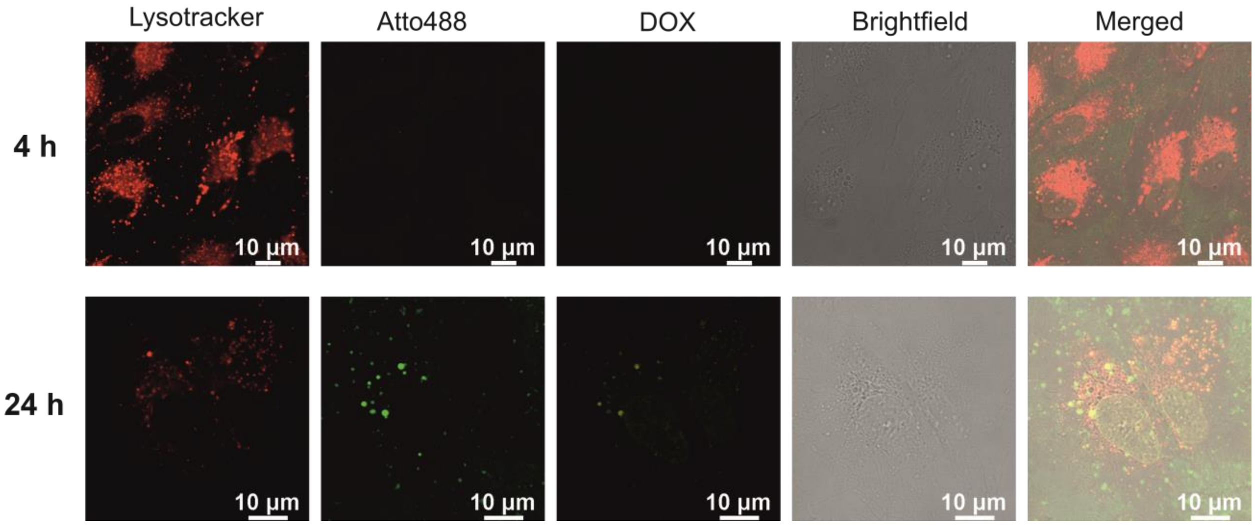

Fig. SI16 Live confocal images of ARPE-19 treated with 24HB-Atto488-DOX for up to 24 h. After 4 h of incubation with the treatments, cells were stained with LysoTracker^{TM} Deep Red (red) and imaged (top row). Majority of 24HB-Atto488-DOX (Atto488 channel, green) appeared outside of the cells, while some DOX (DOX channel, yellow) was detectable in the nucleus. The incubation was continued for up to 24 h (bottom row), however, few more 24HB-Atto488-DOX entered the cells. DOX became more clearly visible in the nucleus after 24 h. No colocalization between 24HB-Atto488-DOX and the lysosomes (Lysotracker channel, red) was detectable even after 24 h. (Note: 20 000 cells/well seeded overnight, 24HB-Atto488-DOX equaled 10.6 nM DONs and 20 µM DOX concentration, no wash before imaging).

SI17: Confocal Images: 24HB-Atto488 with CellMask™ Deep Red in ARPE-19 (24 h)

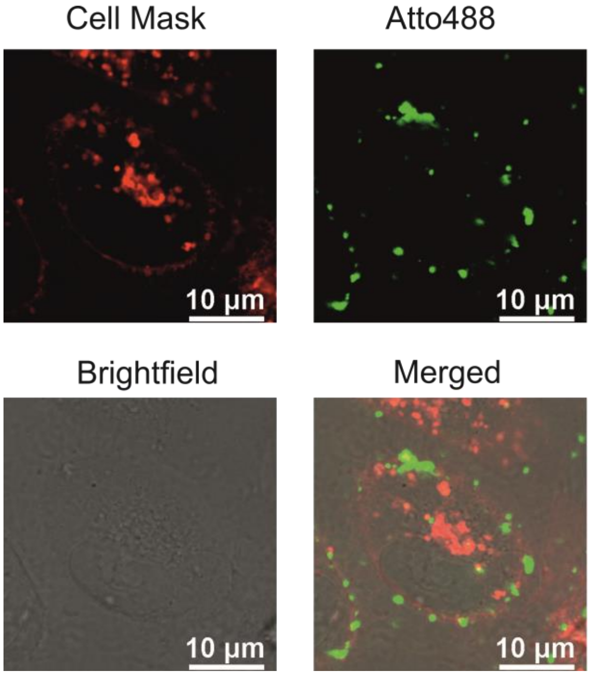

Fig. SI17 Live confocal images of ARPE-19 cells treated with 24HB-Atto488 (10.6 nM) for 24 h. The cell outline of ARPE-19 became more apparent through staining the cell membranes with CellMask™ Deep Red (CellMask channel, red). After 24 h, few 24HB-Atto488 (Atto488 channel, green) had breached the cell membrane, but most seemed to be closely attached or located around the membrane.

SI18: Confocal Images: 24HB-Atto488 with Cell Mask^{TM} in ARPE-19 (24 h, Z-stack/3D)

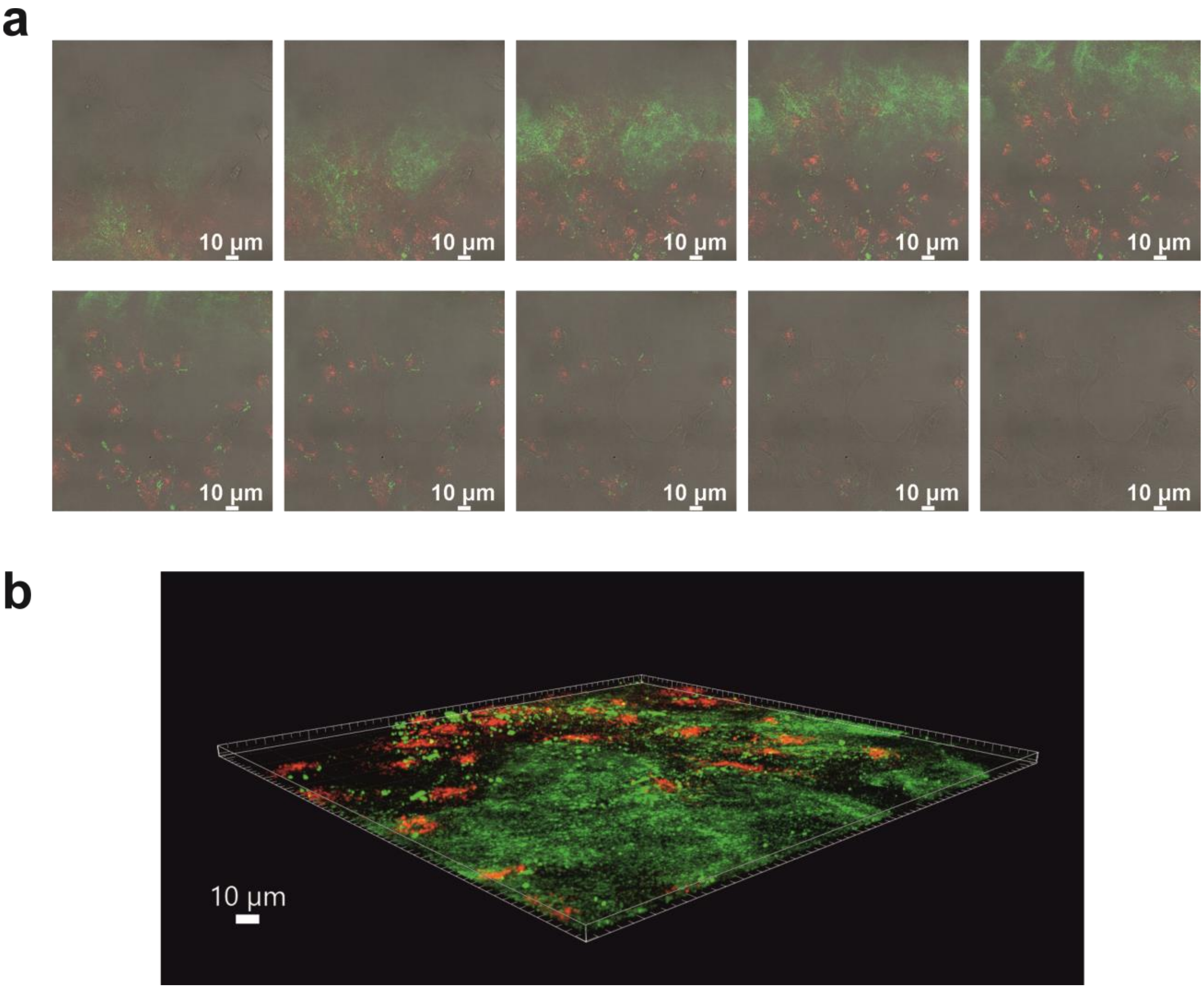

Fig. SI18 Live confocal images of ARPE-19 cells treated with 24HB-Atto488 (10.6 nM) for 24 h (Z-stack/3D). Both in the **a.** Z-stack series and **b.** 3D-image, the cell outline of ARPE-19 was apparent through staining its cell membranes with CellMask^{TM} Deep Red (red). While some 24HB-Atto488 (green) had entered the cell, many appeared to be bound at the bottom of the well where the cells were attached. The images shown were merged from the Cell Mask, Atto488 and brightfield channel. The Z-stack series is also provided as a video.

SI19: Confocal Images: 24HB-Atto488-DOX with Cell Mask Deep Red^{TM} in ARPE-19 (24 h, Z-stack/3D)

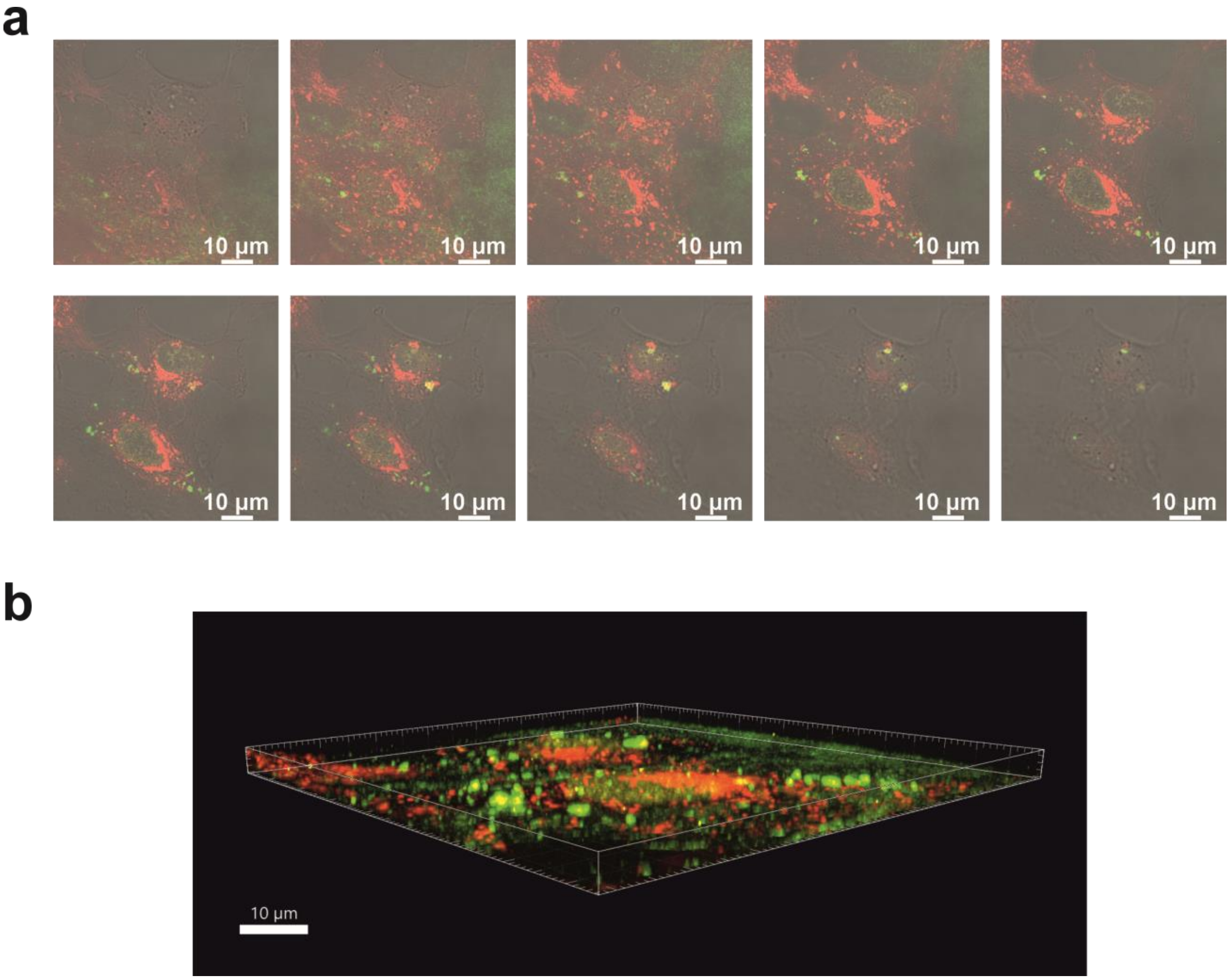

Fig. SI19 Live confocal images of ARPE-19 cells treated with 24HB-Atto488-DOX for 24 h (Z-stack/3D). Both in the **a.** Z-stack series and **b.** 3D-image, the outline of ARPE-19 cells was visualized by staining their cell membranes with CellMask^{TM} Deep Red (red). Some 24HB-Atto488-DOX (green) were in the center of the cell, DOX (yellow) being visible in the nucleus. Mainly, 24HB-Atto488-DOX were stuck around the adherent cells at the bottom, and less were observed on top of the cells. 24 HB-Atto488-DOX equaled 10.6 nM DONs and 20 µM DOX in concentration. The images shown were merged from the Cell Mask, Atto488, DOX and brightfield channel. This and another Z-stack series are also provided as videos.

SI20: Confocal Images: Atto488-strands with Cell Mask^{TM} Deep Red in ARPE-19 (24 h, Z-stack/3D)

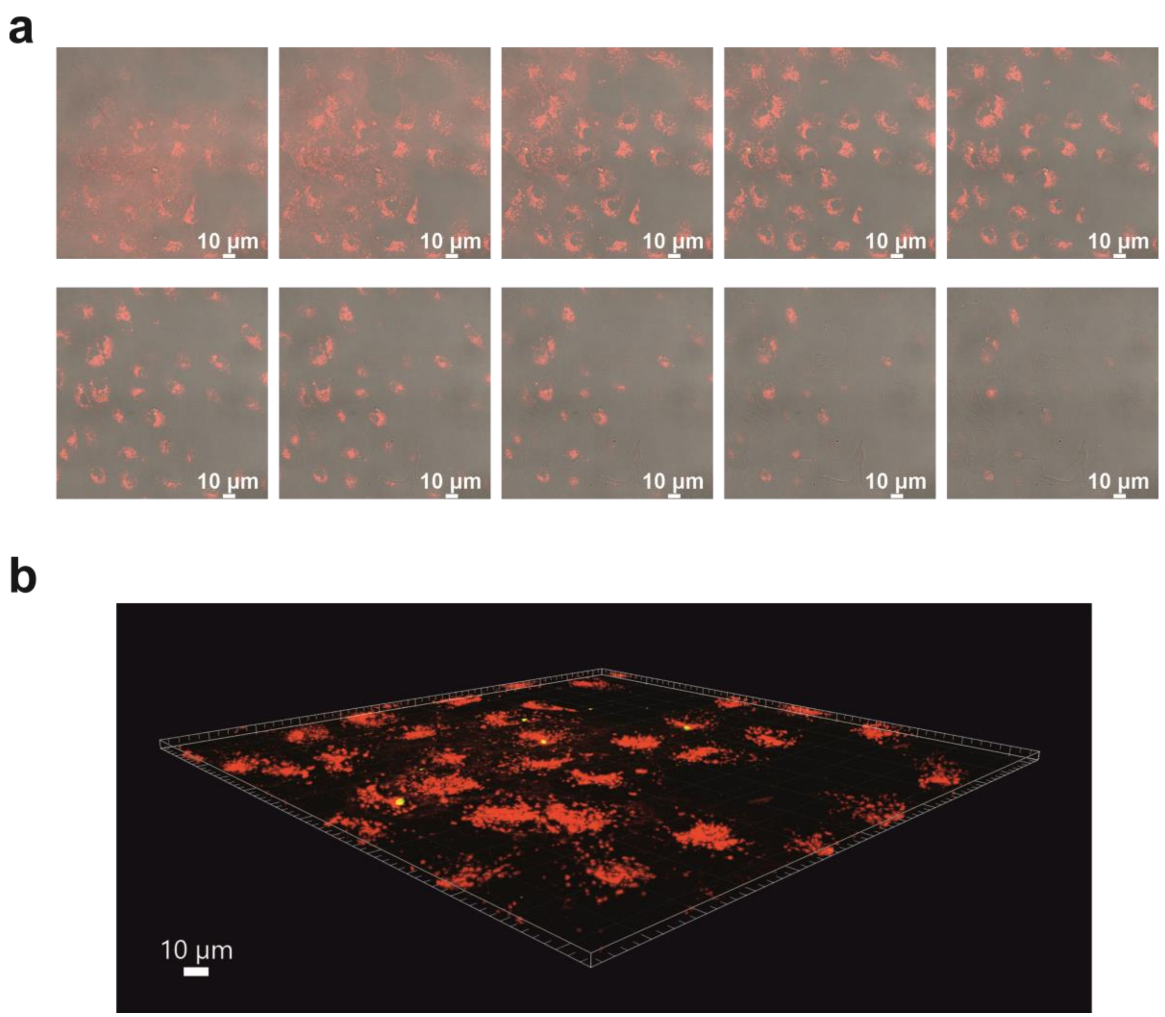

Fig. SI20 Live confocal images of ARPE-19 cells treated with Atto488-strands for 24 h (Z-stack/3D). To verify that the observed signal for 24HB-Atto488 inside and at the base of the cells was not from free dislodged Atto488-fluorophores (green), the Atto488-strands (without annealed 24HB) was incubated with ARPE-19 for 24 h with similar concentrations to be found in 10.6 nM DONs (254 nM Atto488). Both the **a.** Z-stack series and **b.** 3D-image revealed that Atto488-strands had little binding affinity to the cells and very rarely got taken up by cells. It seemed that only when bound to DONs, its uptake as well the strong attachment around the cell was observable. In three single locations, Atto488 signal were observable in the cells, but that seems in comparison to the extent of 24HB-Atto488 (SI17 and SI18) negligible. The cell membranes were stained with CellMask^{TM} Deep Red (red). The Z-stack images shown were merged from the Cell Mask, Atto488 and brightfield channel. The Z-stack series is also provided as a video.

References

1 V. Linko, B. Shen, K. Tapio, J. J. Toppari, M. A. Kostiainen and S. Tuukkanen, *Sci. Rep.*, 2015, **5**, 15634.
2 H. Ijäs, B. Shen, A. Heuer-Jungemann, A. Keller, M. A. Kostiainen, T. Liedl, J. A. Ihalainen and V. Linko, *Nucleic Acids Res.*, 2021, **49**, 3048–3062.
3 S. Julin, Nonappa, B. Shen, V. Linko and M. A. Kostiainen, *Angew. Chem. Int. Ed.*, 2021, **60**, 827–833.
4 E. Stahl, T. G. Martin, F. Praetorius and H. Dietz, *Angew. Chem. Int. Ed.*, 2014, **53**, 12735–12740.
5 C. E. Castro, F. Kilchherr, D.-N. Kim, E. L. Shiao, T. Wauer, P. Wortmann, M. Bathe and H. Dietz, *Nat. Methods*, 2011, **8**, 221–229.
6 I. Seitz, H. Ijäs, V. Linko and M. A. Kostiainen, *ACS Appl. Mater. Interfaces*, 2022, **14**, 38515–38524.
7 A. M. Hung, C. M. Micheel, L. D. Bozano, L. W. Osterbur, G. M. Wallraff and J. N. Cha, *Nat. Nanotechnol.*, 2010, **5**, 121–126.
8 C. Pérez-Arnaiz, N. Busto, J. M. Leal and B. García, *J. Phys. Chem. B*, 2014, **118**, 1288–1295.
