# Supplementary figures and images for: Doxorubicin-loaded DNA origami nanostructures: stability in vitreous and their uptake and toxicity in ocular cells
Source: Nanoscale. 2024 Aug 27;16(37):17585–98. doi: 10.1039/d4nr01995d (PMC11372452; doi:10.1039/d4nr01995d)

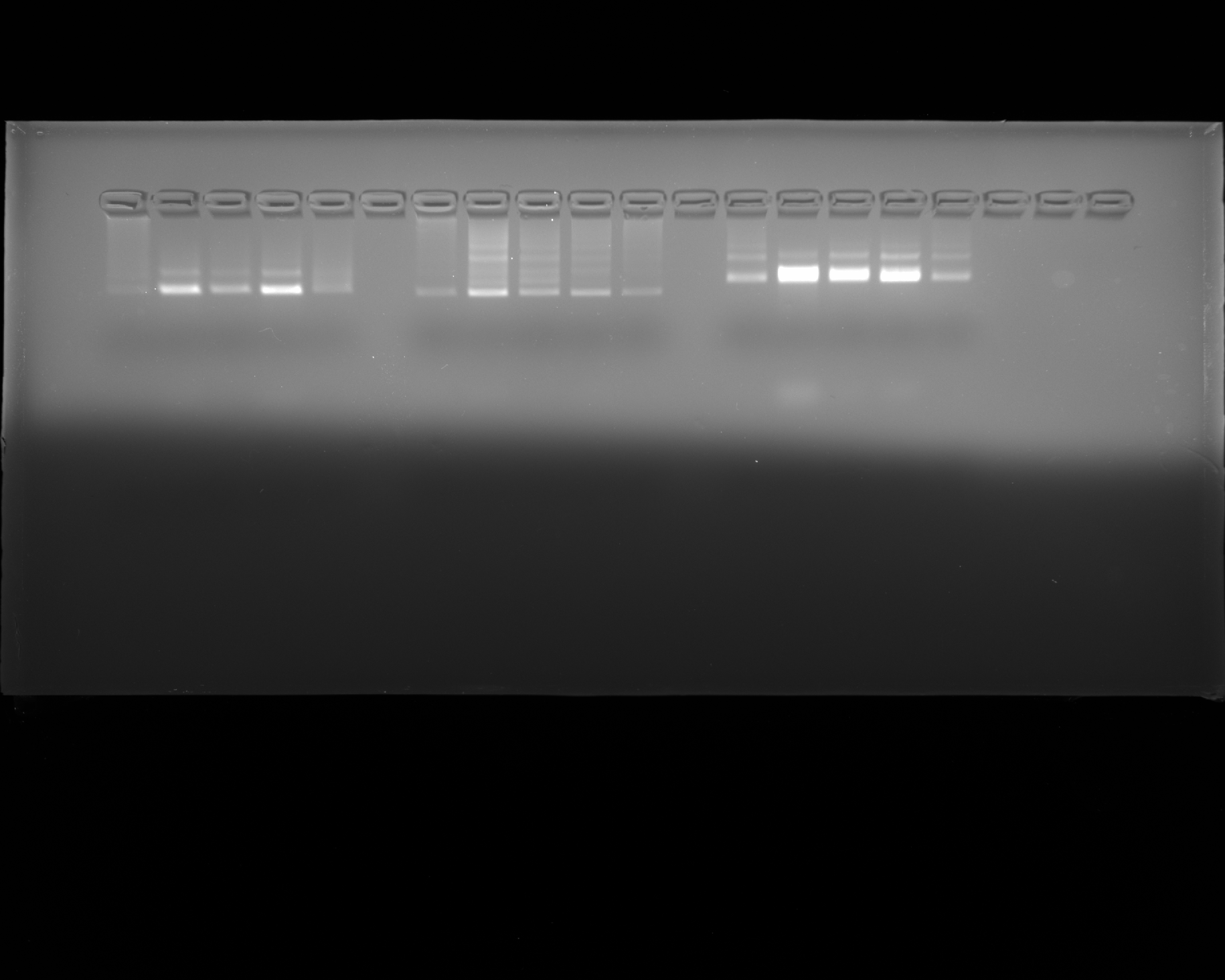

Supplement: NR-016-D4NR01995D-s003 [file NR-016-D4NR01995D-s003.zip › unedited original gel images/Main Gel 1d.tif]

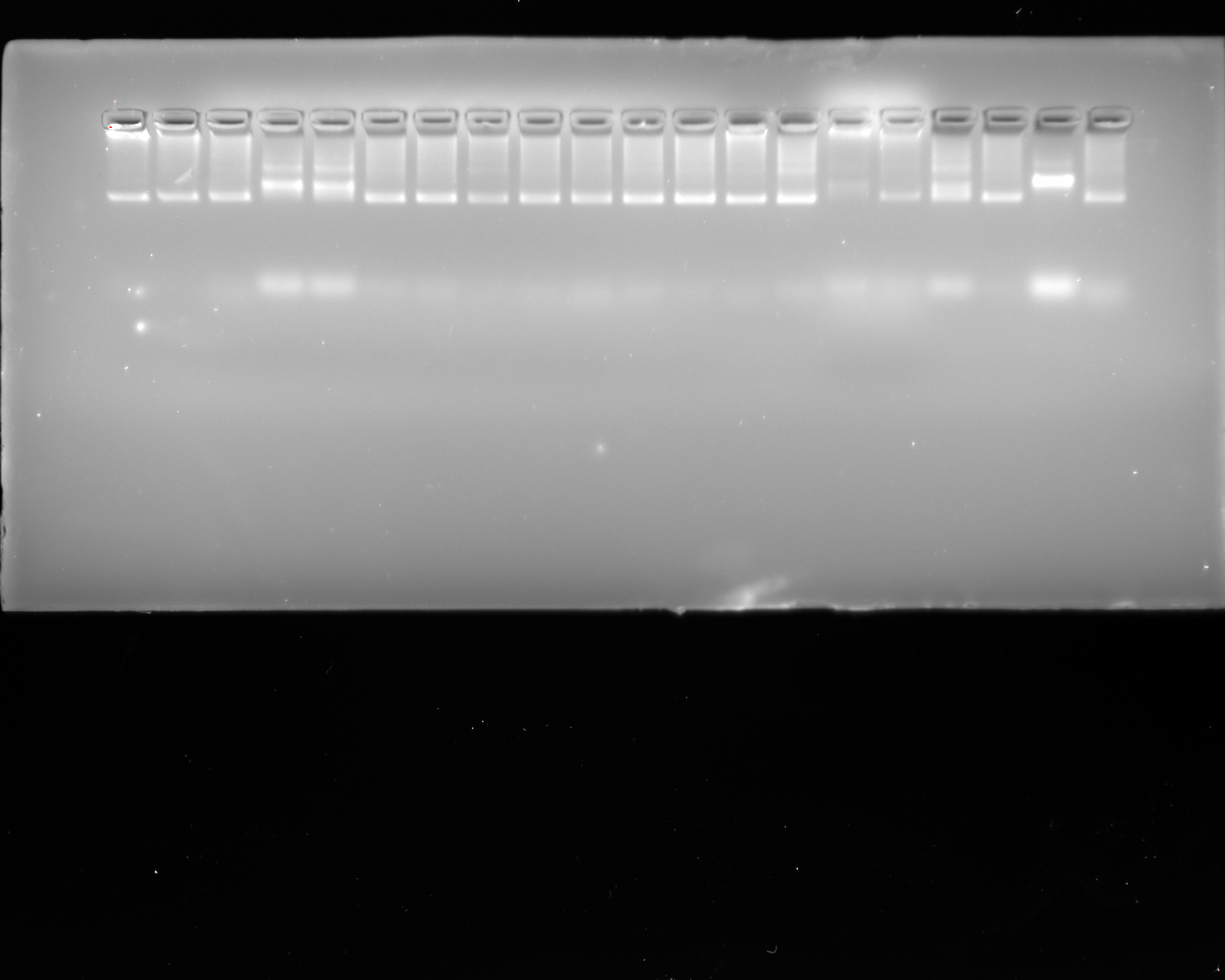

Supplement: NR-016-D4NR01995D-s003 [file NR-016-D4NR01995D-s003.zip › unedited original gel images/Main Gel 2a Atto488.tif]

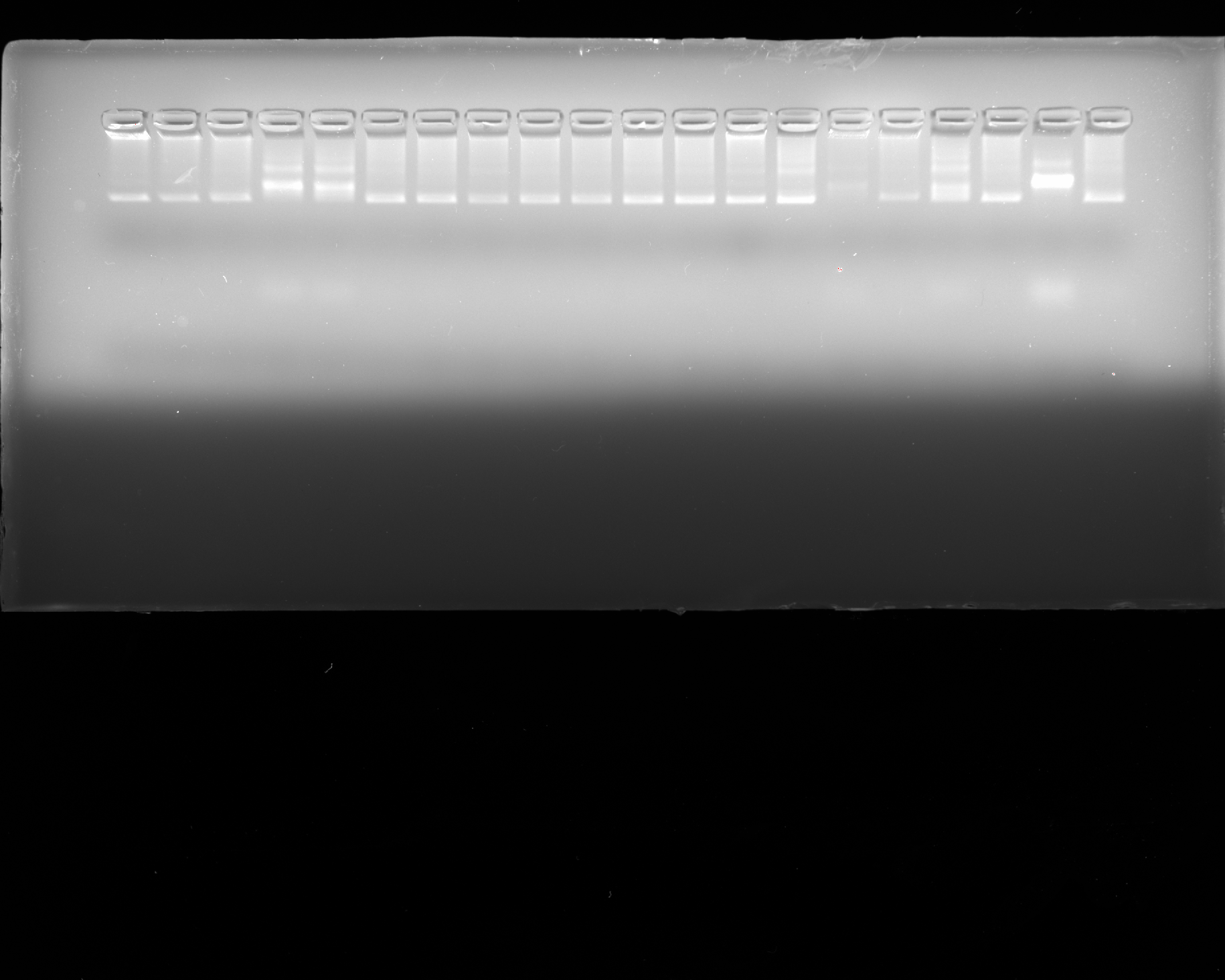

Supplement: NR-016-D4NR01995D-s003 [file NR-016-D4NR01995D-s003.zip › unedited original gel images/Main Gel 2a EtBr.tif]

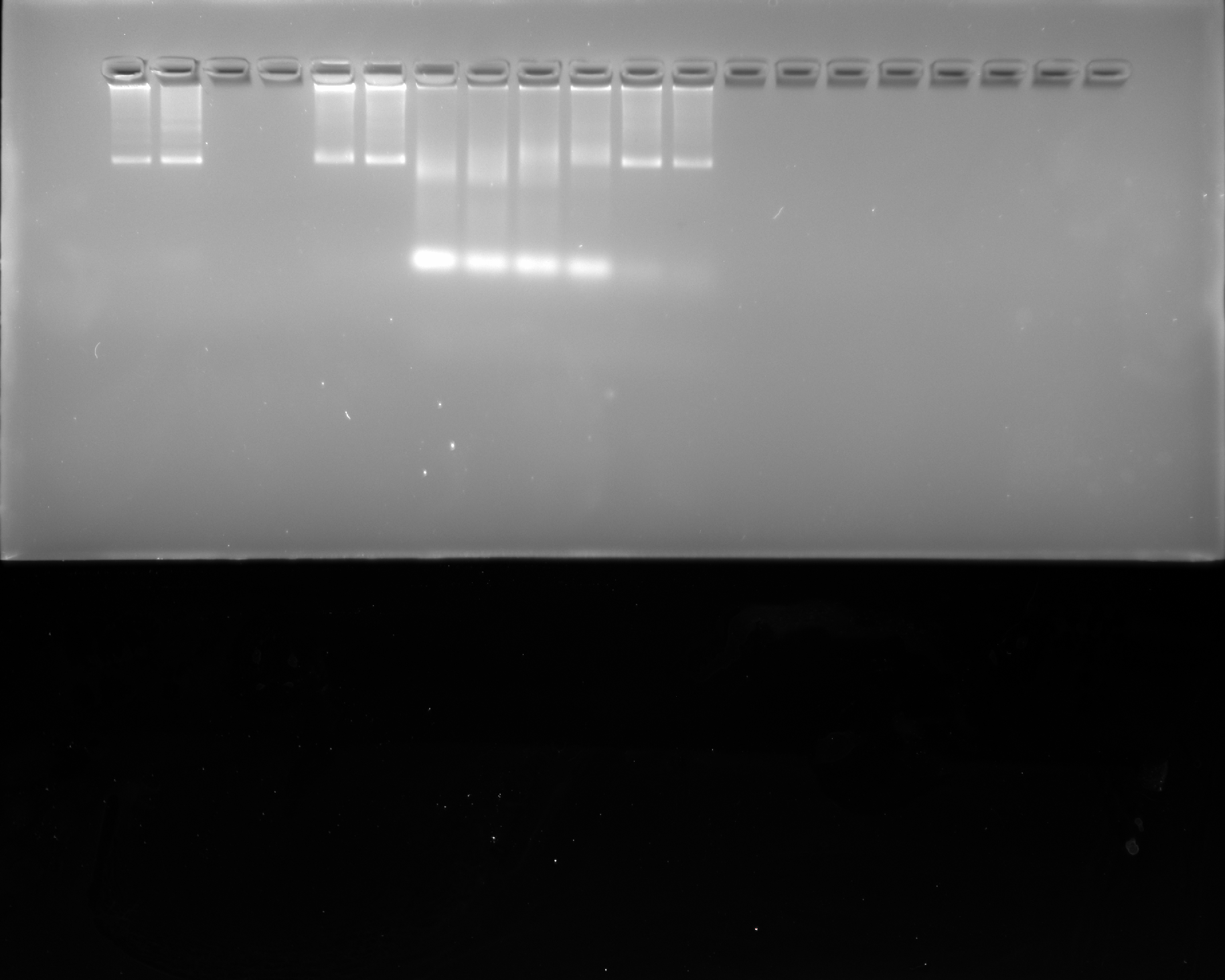

Supplement: NR-016-D4NR01995D-s003 [file NR-016-D4NR01995D-s003.zip › unedited original gel images/Main Gel 2b Atto488.tif]

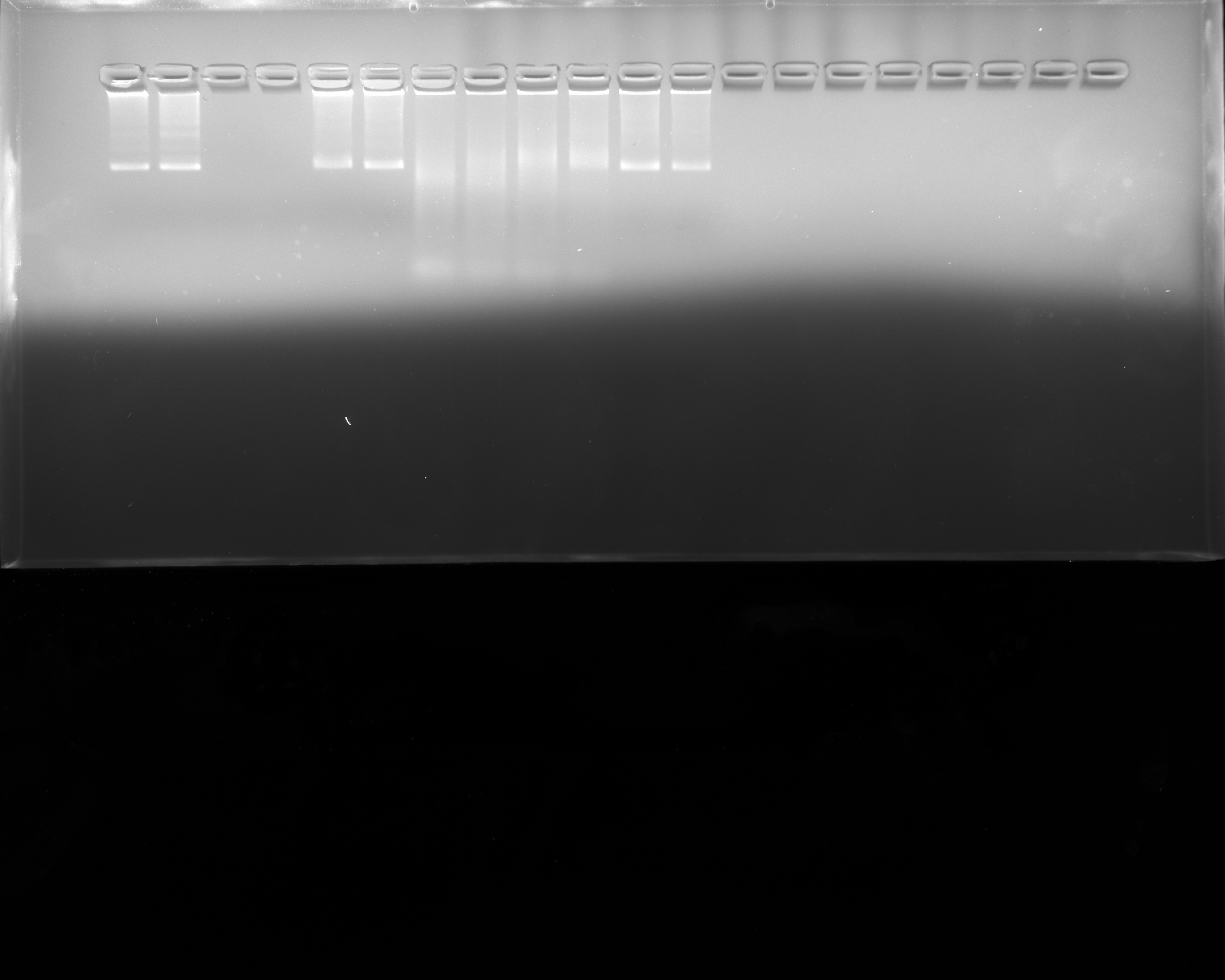

Supplement: NR-016-D4NR01995D-s003 [file NR-016-D4NR01995D-s003.zip › unedited original gel images/Main Gel 2b EtBr.tif]

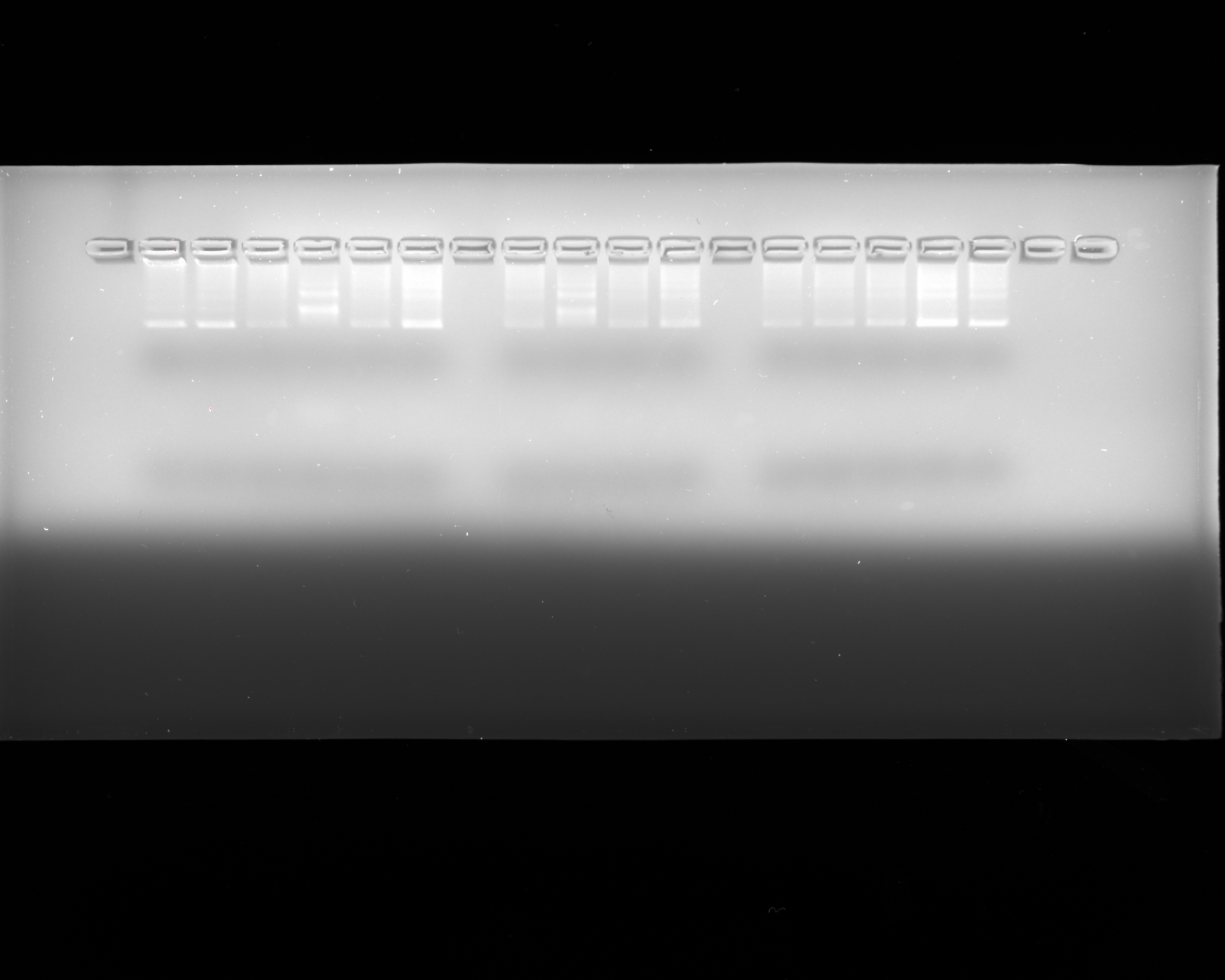

Supplement: NR-016-D4NR01995D-s003 [file NR-016-D4NR01995D-s003.zip › unedited original gel images/SI11a EtBr.tif]

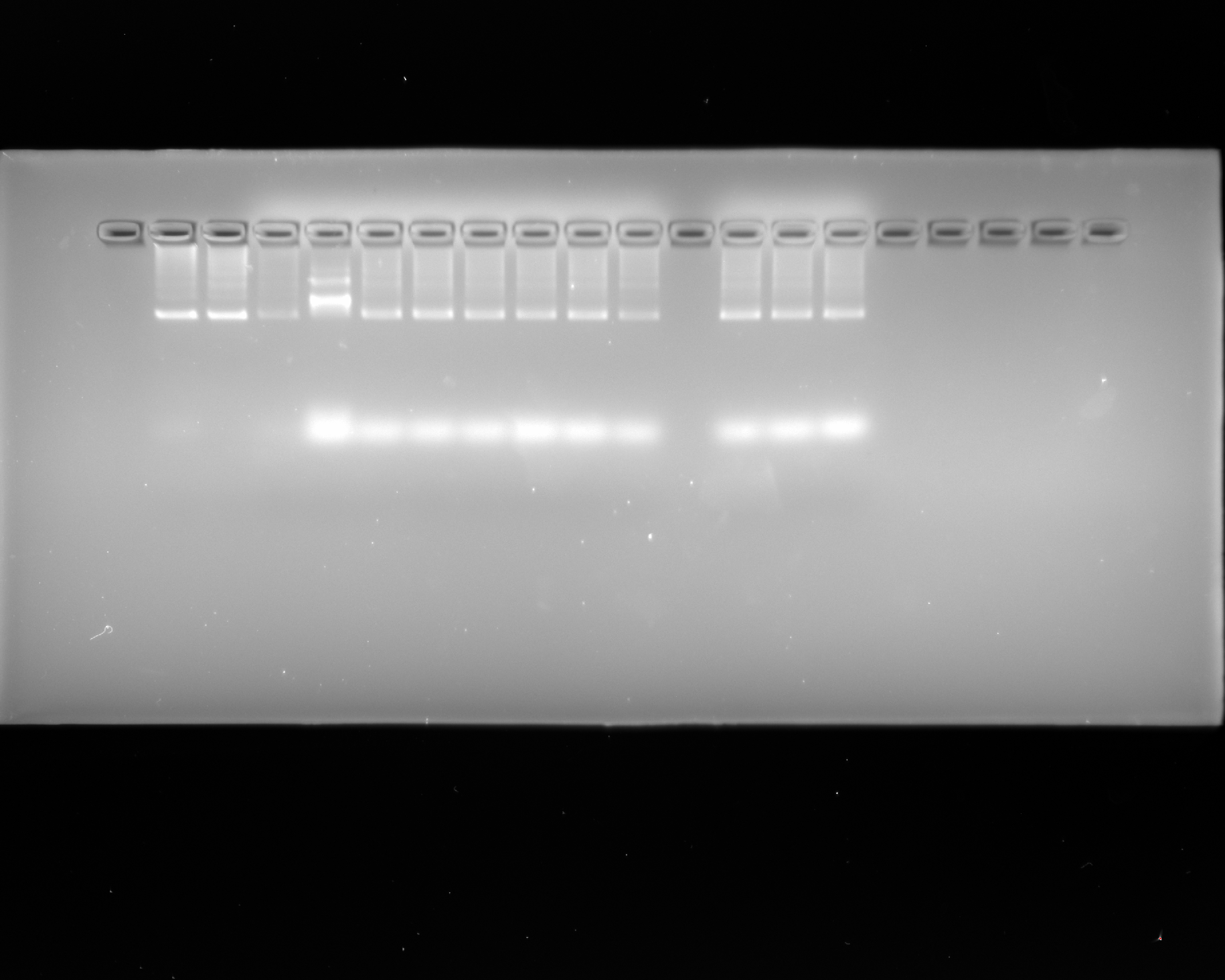

Supplement: NR-016-D4NR01995D-s003 [file NR-016-D4NR01995D-s003.zip › unedited original gel images/SI11b Atto488.tif]

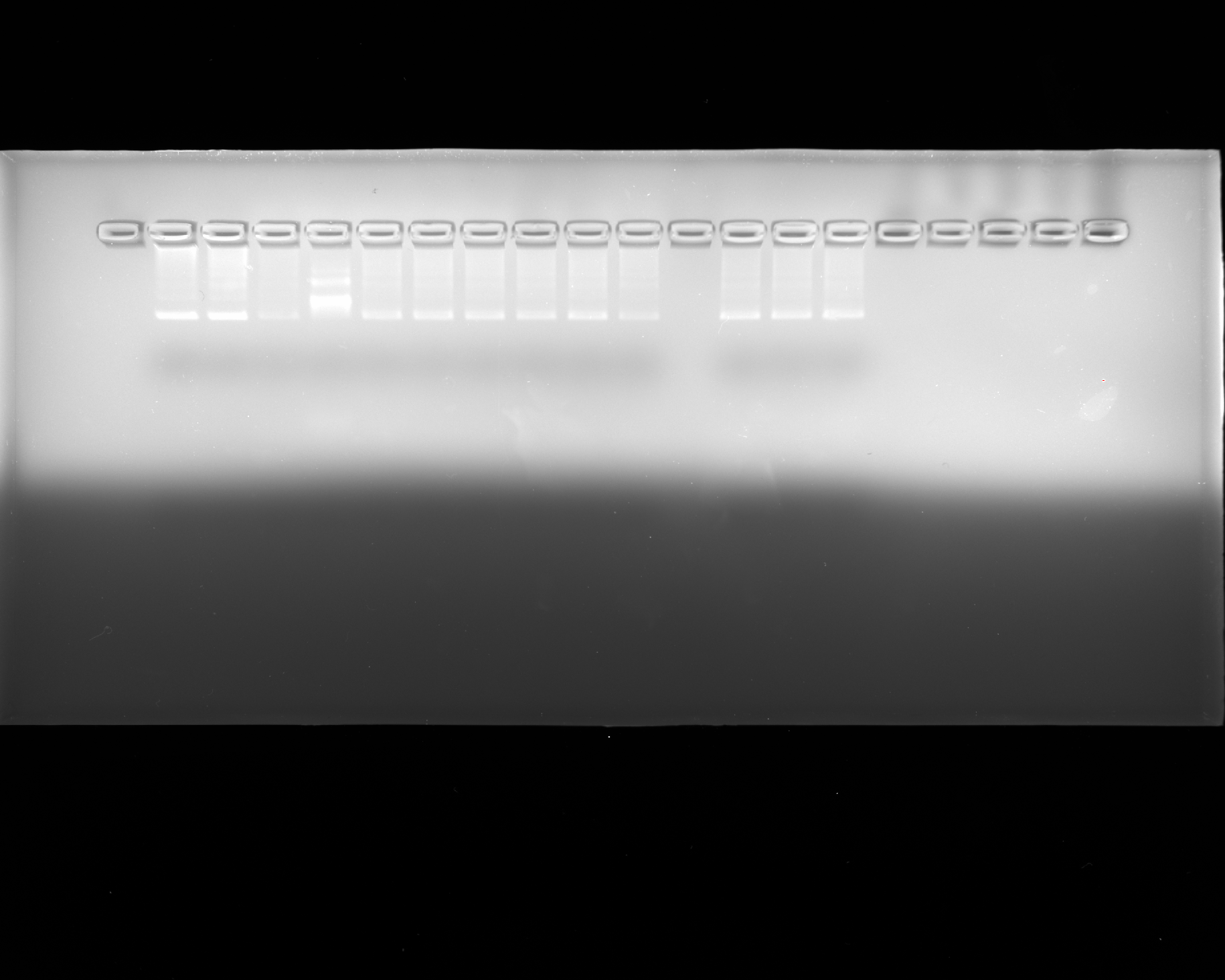

Supplement: NR-016-D4NR01995D-s003 [file NR-016-D4NR01995D-s003.zip › unedited original gel images/SI11b EtBr.tif]

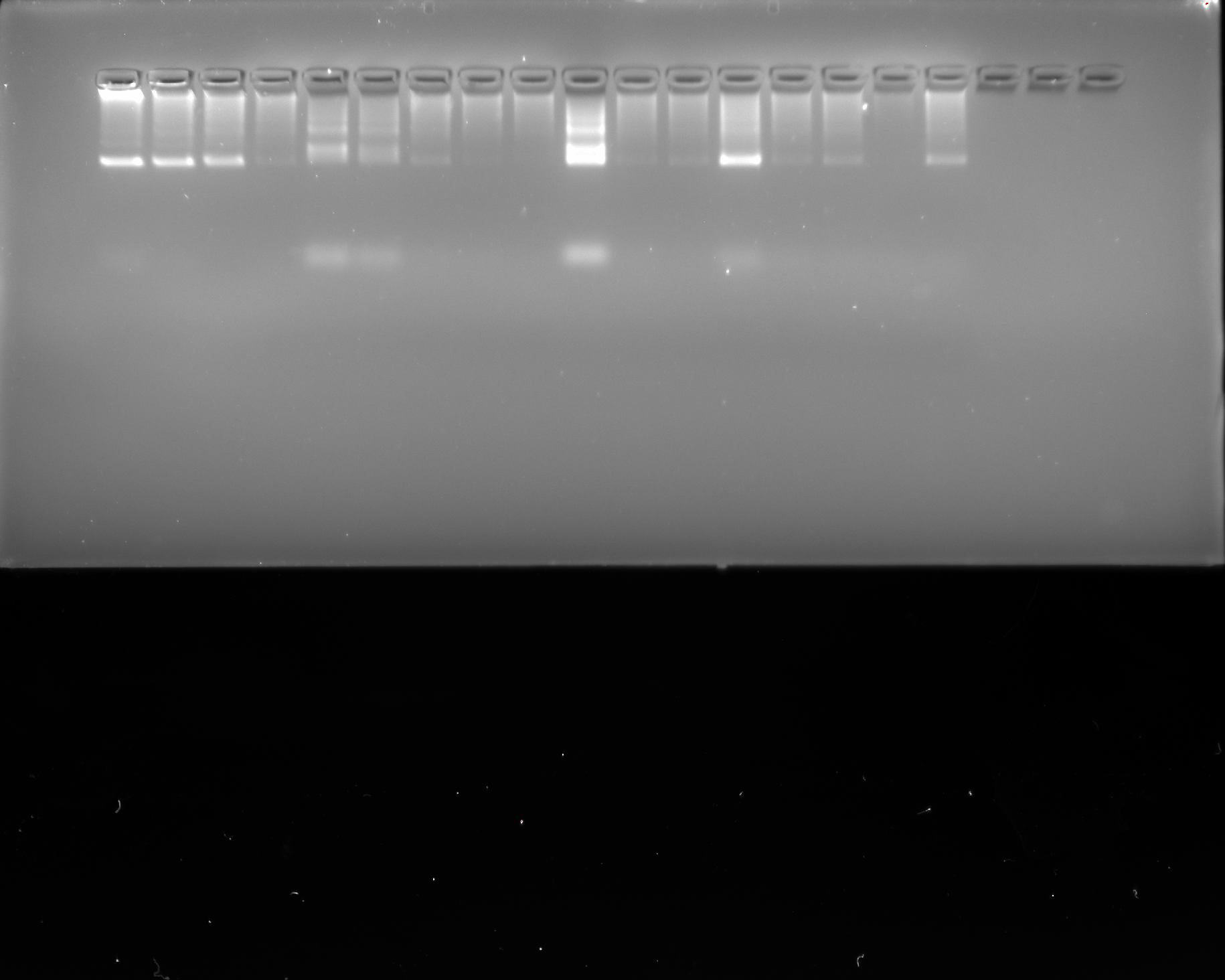

Supplement: NR-016-D4NR01995D-s003 [file NR-016-D4NR01995D-s003.zip › unedited original gel images/Si12 Atto488.tif]

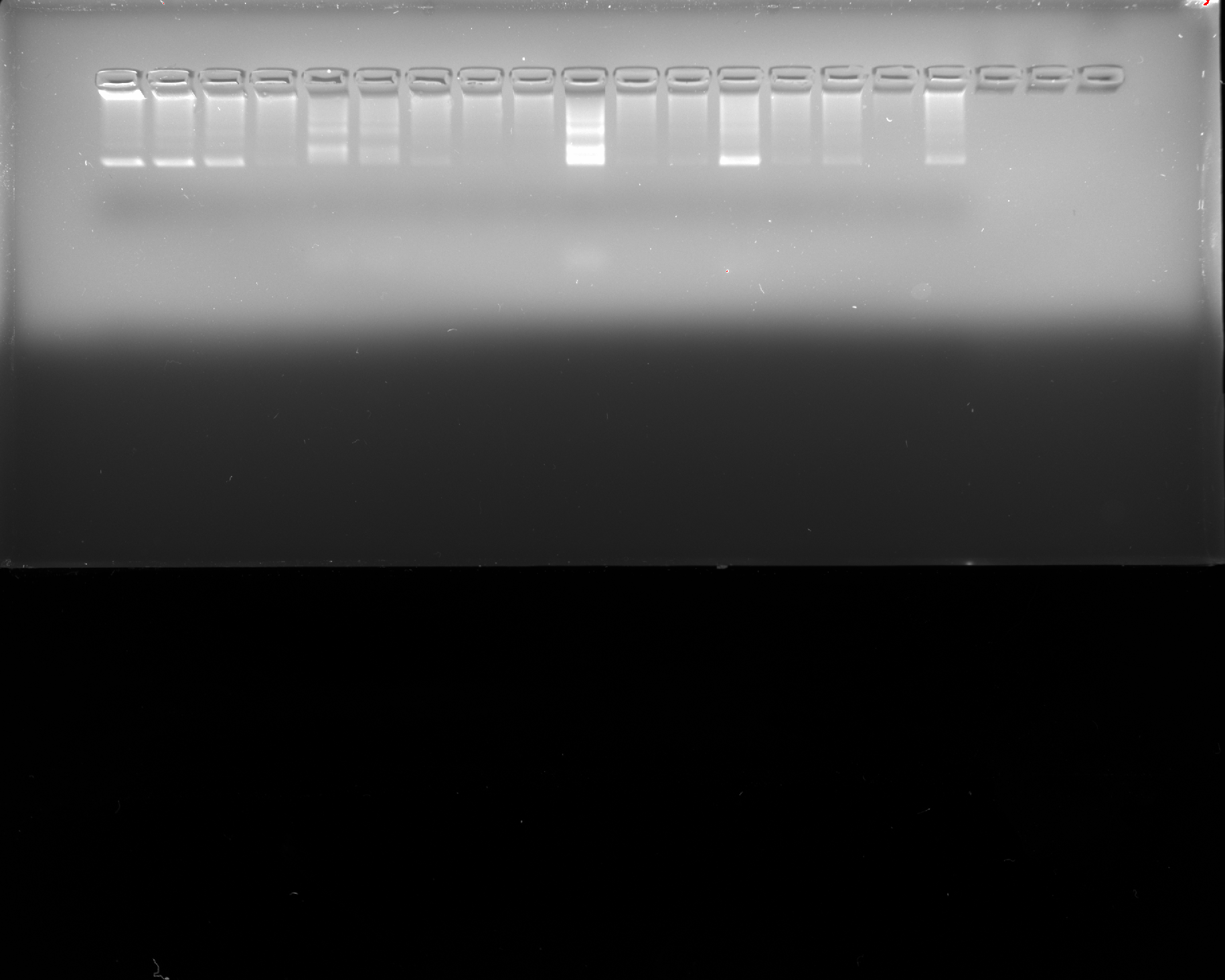

Supplement: NR-016-D4NR01995D-s003 [file NR-016-D4NR01995D-s003.zip › unedited original gel images/Si12 EtBr.tif]

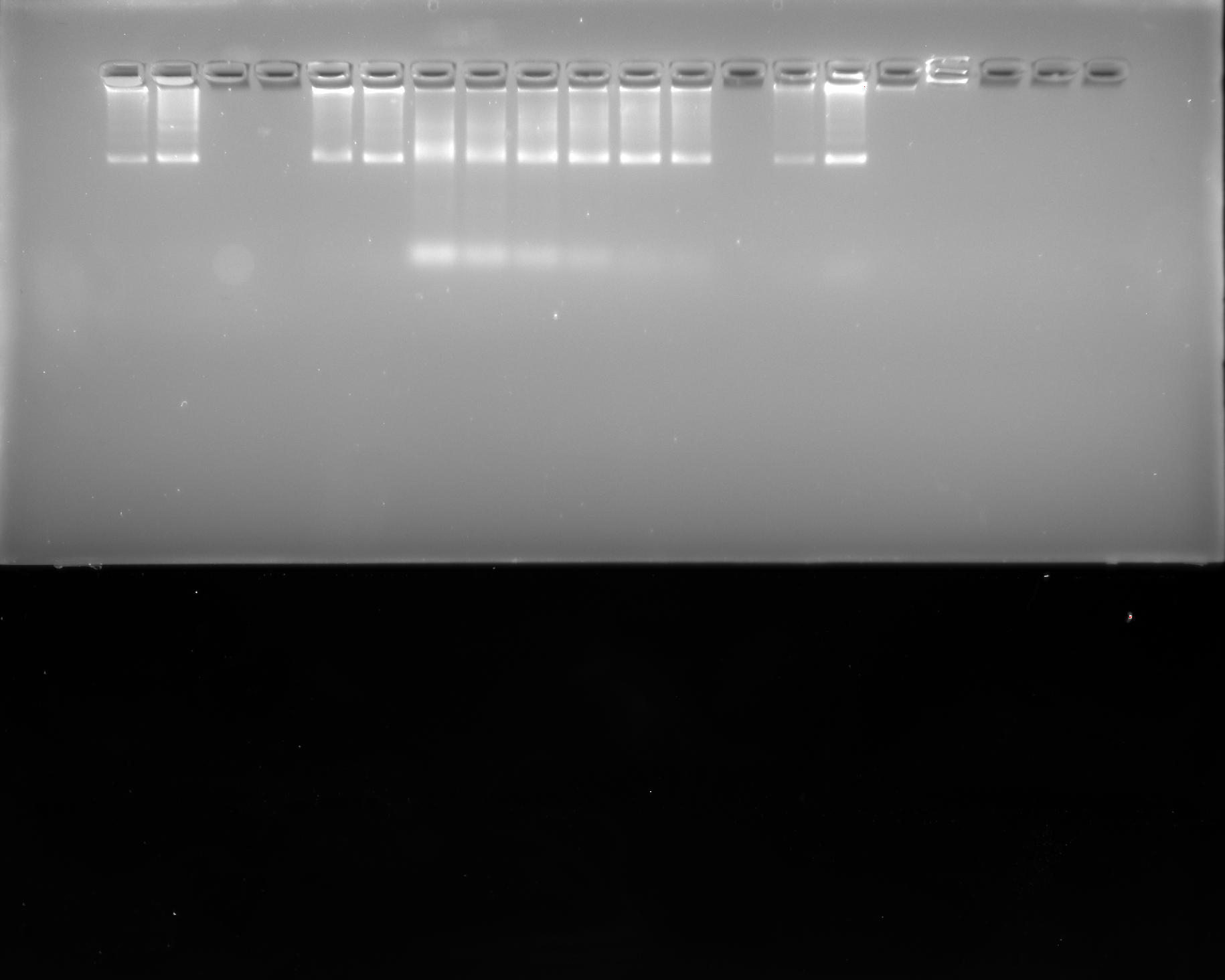

Supplement: NR-016-D4NR01995D-s003 [file NR-016-D4NR01995D-s003.zip › unedited original gel images/Si13 Atto488.tif]

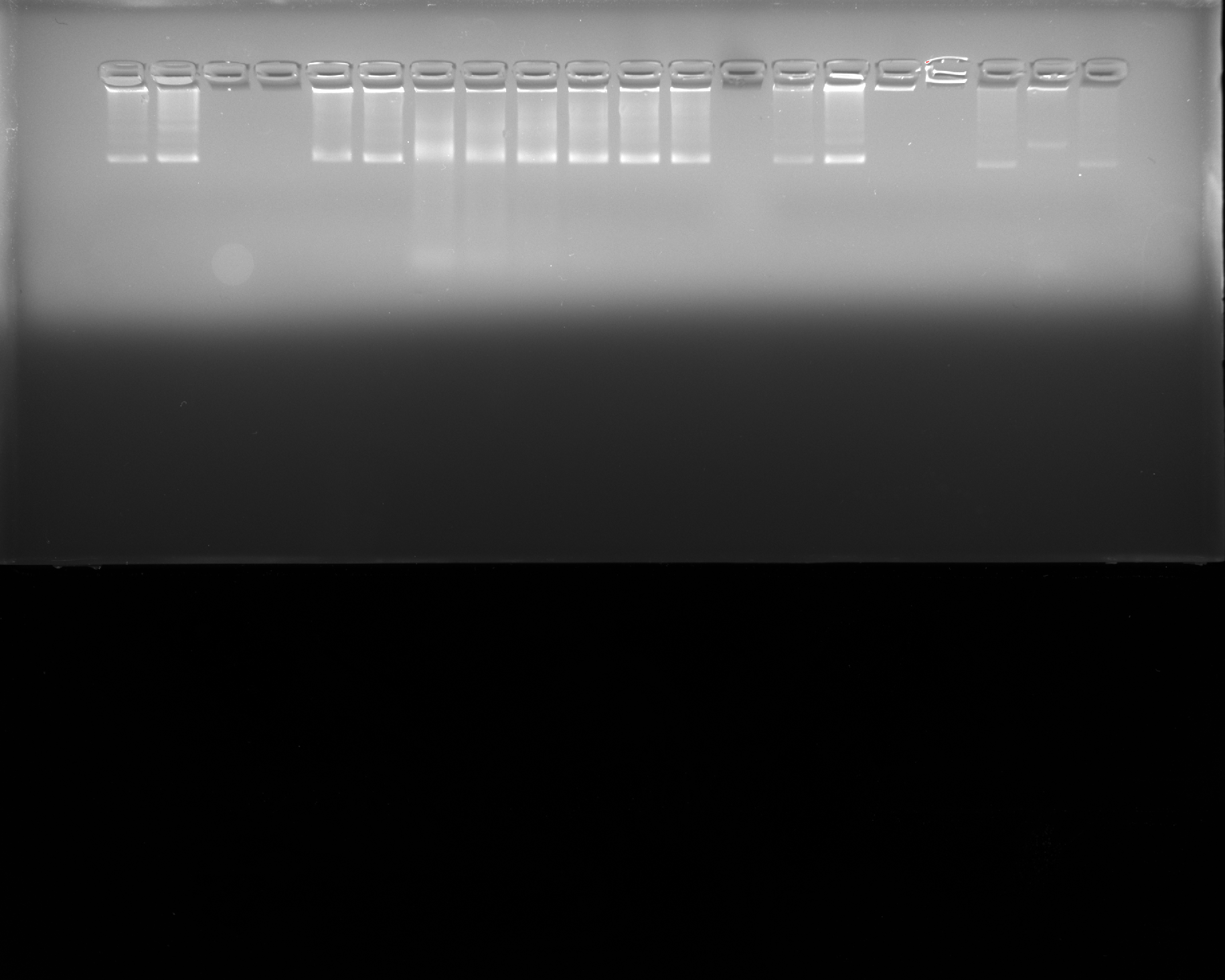

Supplement: NR-016-D4NR01995D-s003 [file NR-016-D4NR01995D-s003.zip › unedited original gel images/Si13 EtBr.tif]

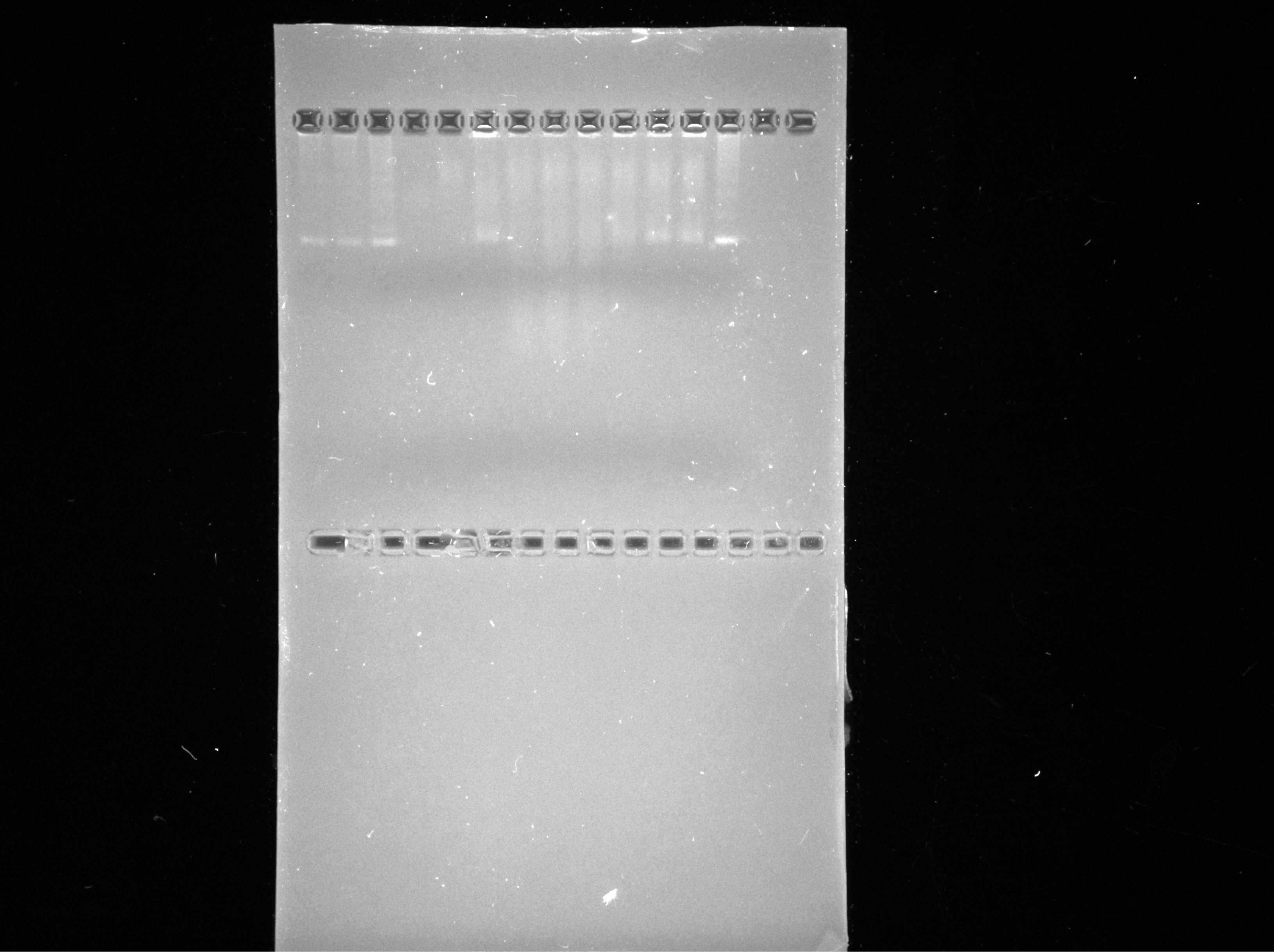

Supplement: NR-016-D4NR01995D-s003 [file NR-016-D4NR01995D-s003.zip › unedited original gel images/SI14 7 days.tif]

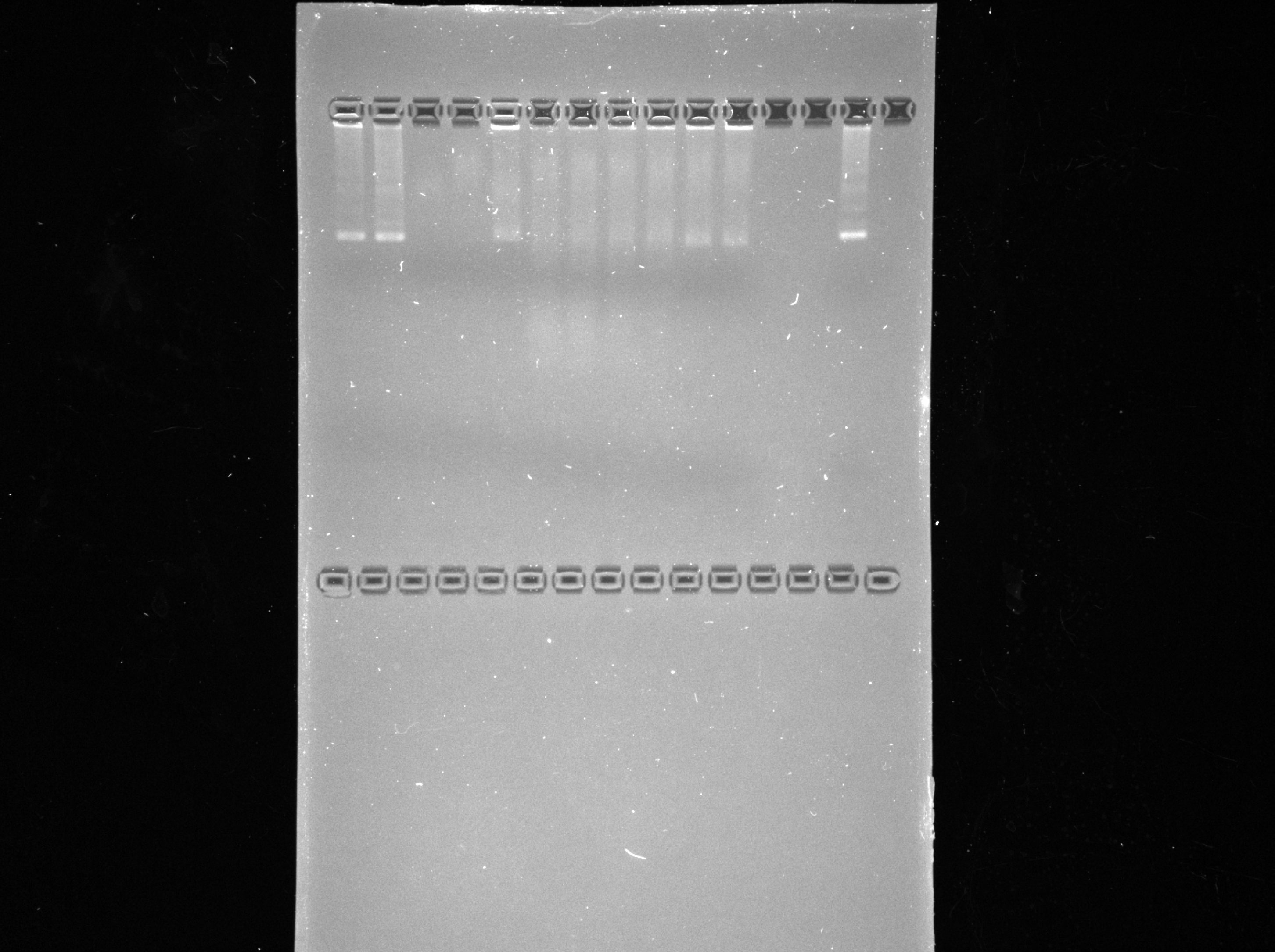

Supplement: NR-016-D4NR01995D-s003 [file NR-016-D4NR01995D-s003.zip › unedited original gel images/SI14 72h.tif]

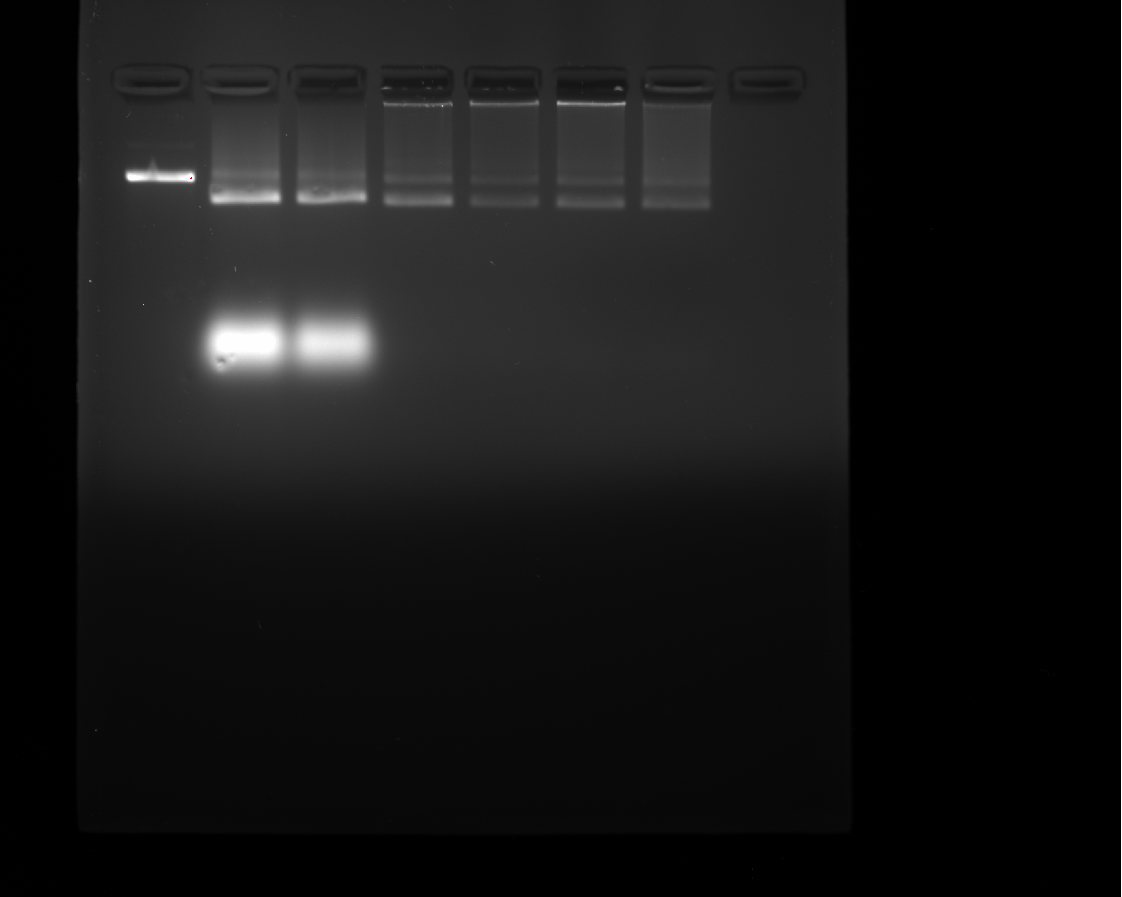

Supplement: NR-016-D4NR01995D-s003 [file NR-016-D4NR01995D-s003.zip › unedited original gel images/SI3a EtBr.tif]

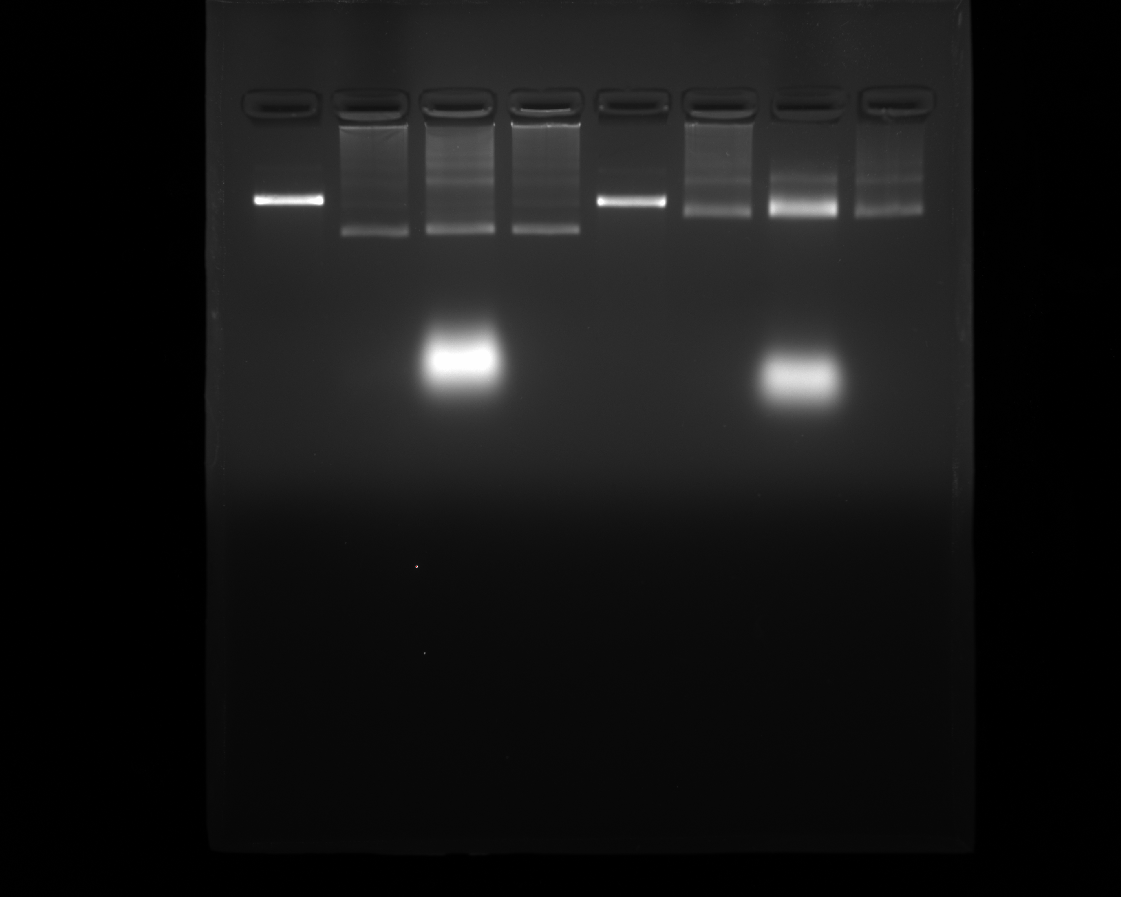

Supplement: NR-016-D4NR01995D-s003 [file NR-016-D4NR01995D-s003.zip › unedited original gel images/SI3b EtBr.tif]

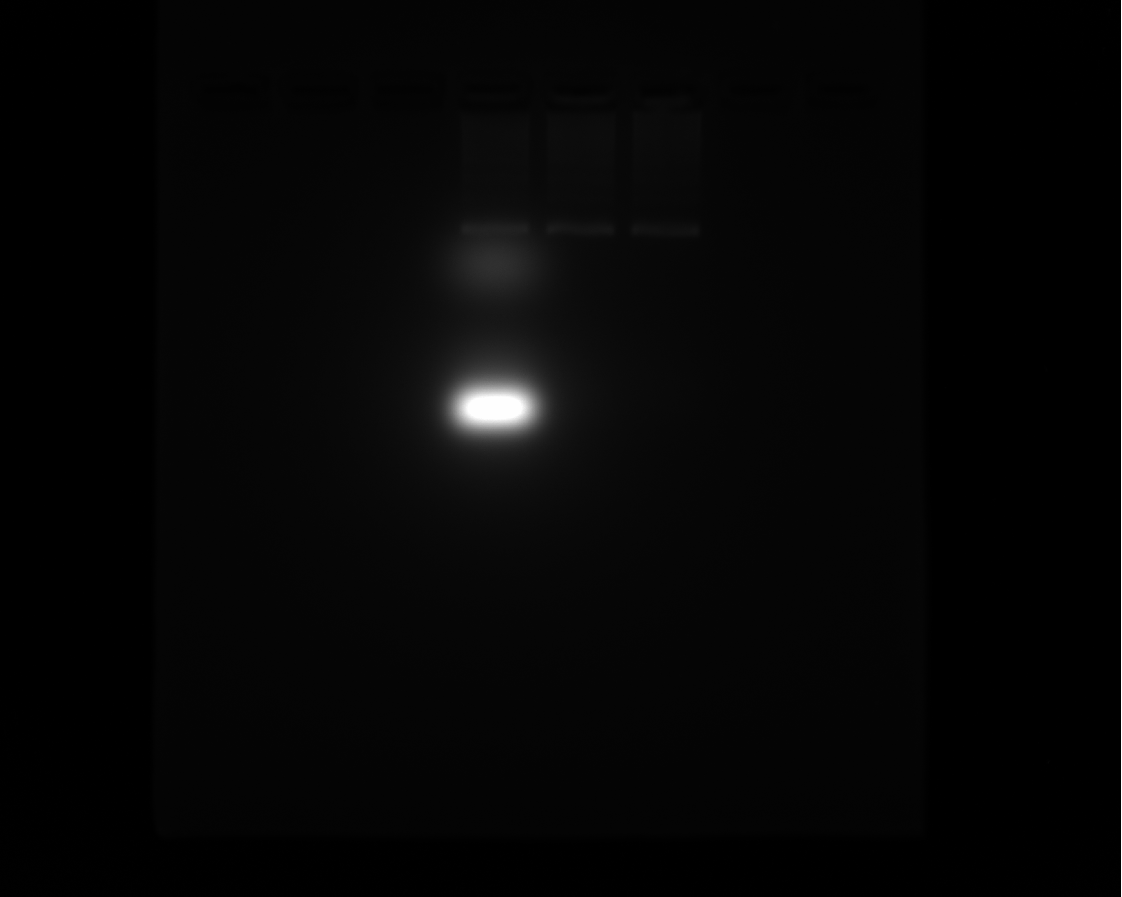

Supplement: NR-016-D4NR01995D-s003 [file NR-016-D4NR01995D-s003.zip › unedited original gel images/SI4b Atto488 left.tif]

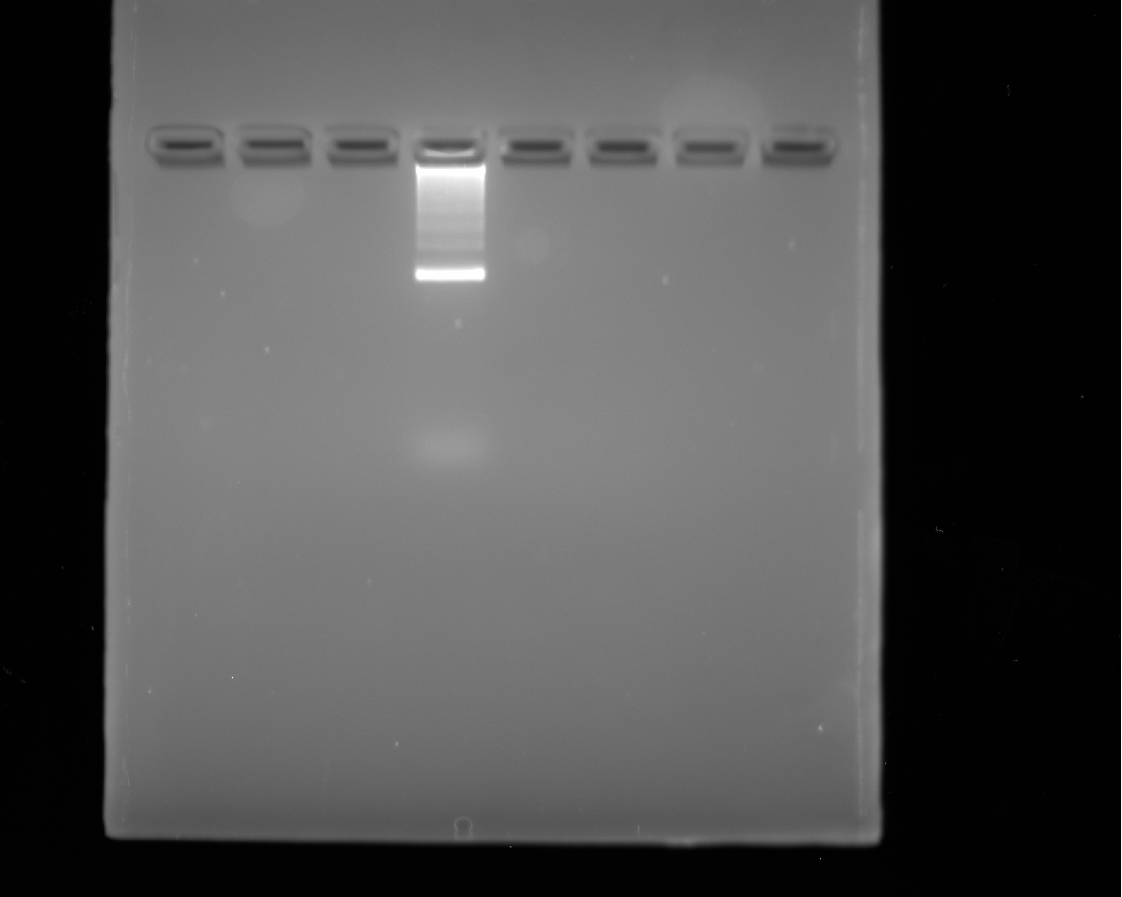

Supplement: NR-016-D4NR01995D-s003 [file NR-016-D4NR01995D-s003.zip › unedited original gel images/SI4b Atto488 right.tif]

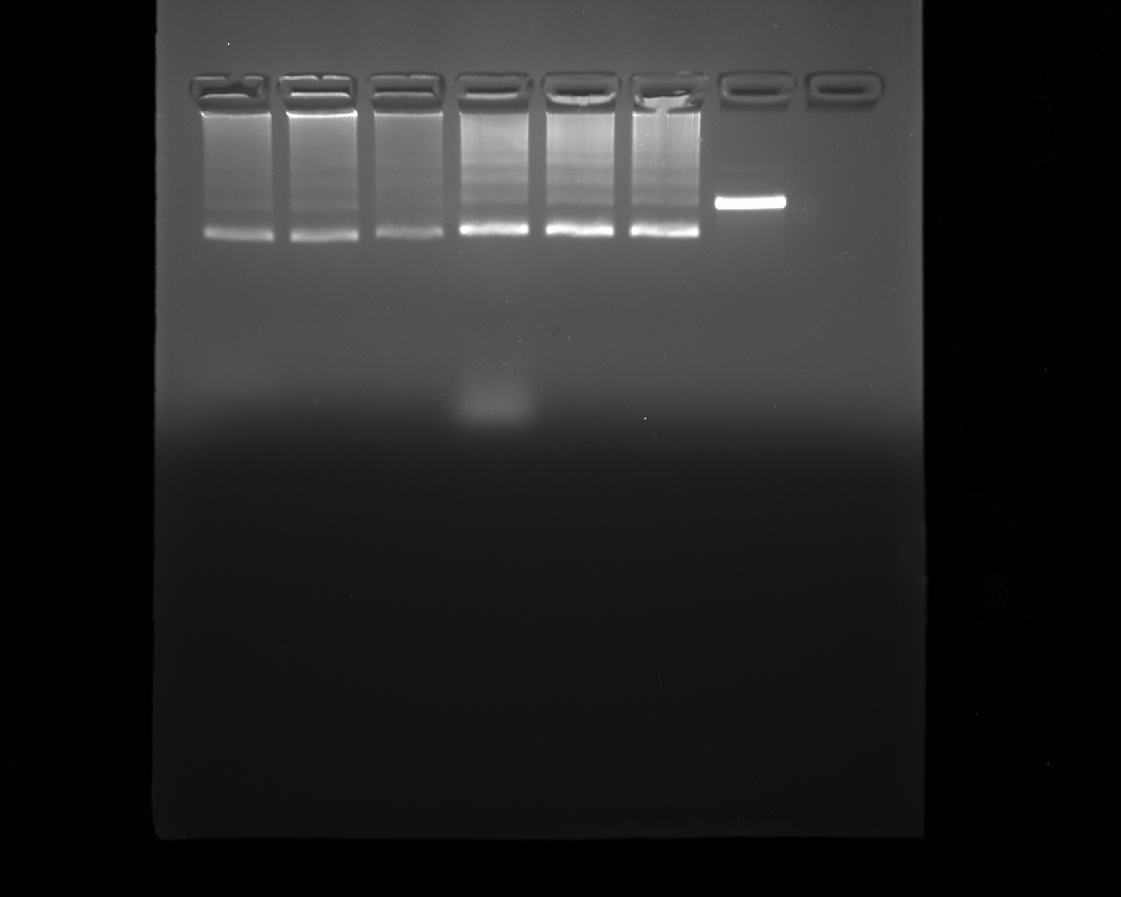

Supplement: NR-016-D4NR01995D-s003 [file NR-016-D4NR01995D-s003.zip › unedited original gel images/SI4b EtBr left.tif]

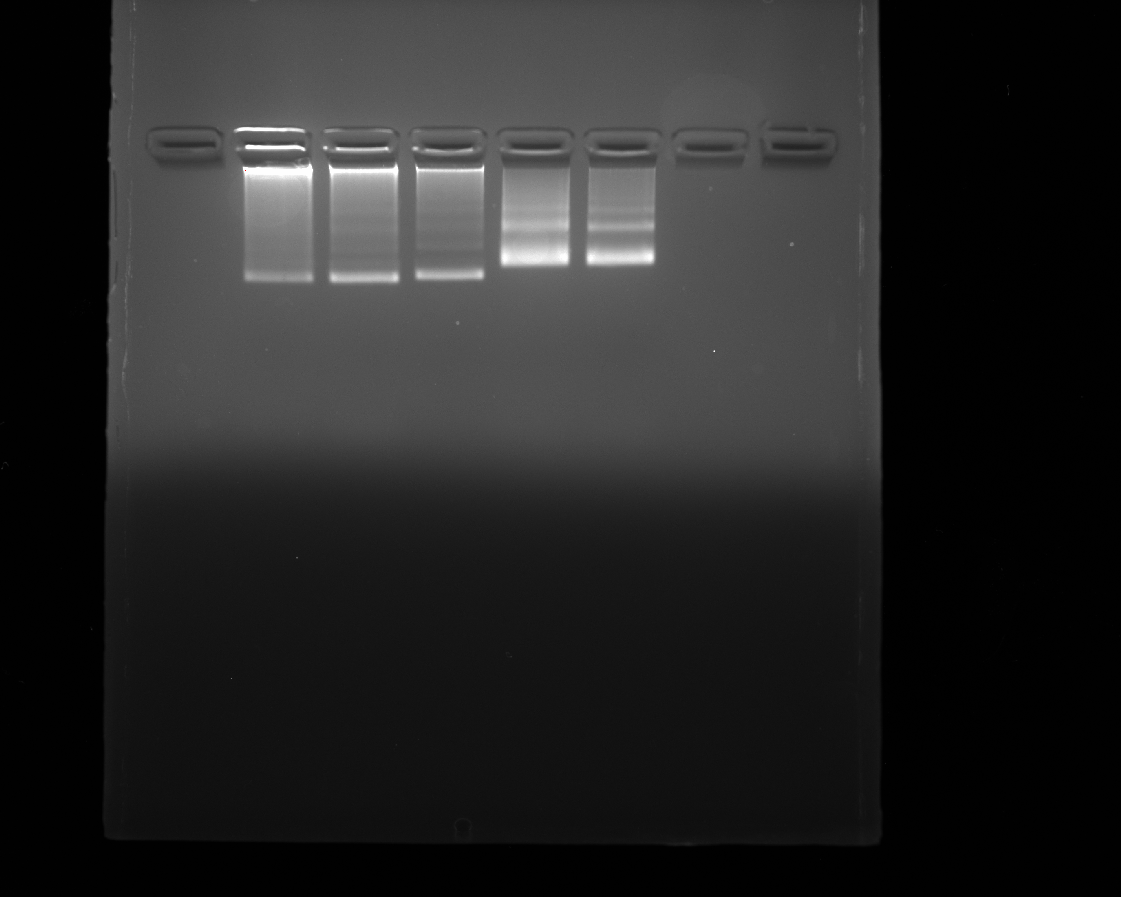

Supplement: NR-016-D4NR01995D-s003 [file NR-016-D4NR01995D-s003.zip › unedited original gel images/SI4b EtBr right.tif]

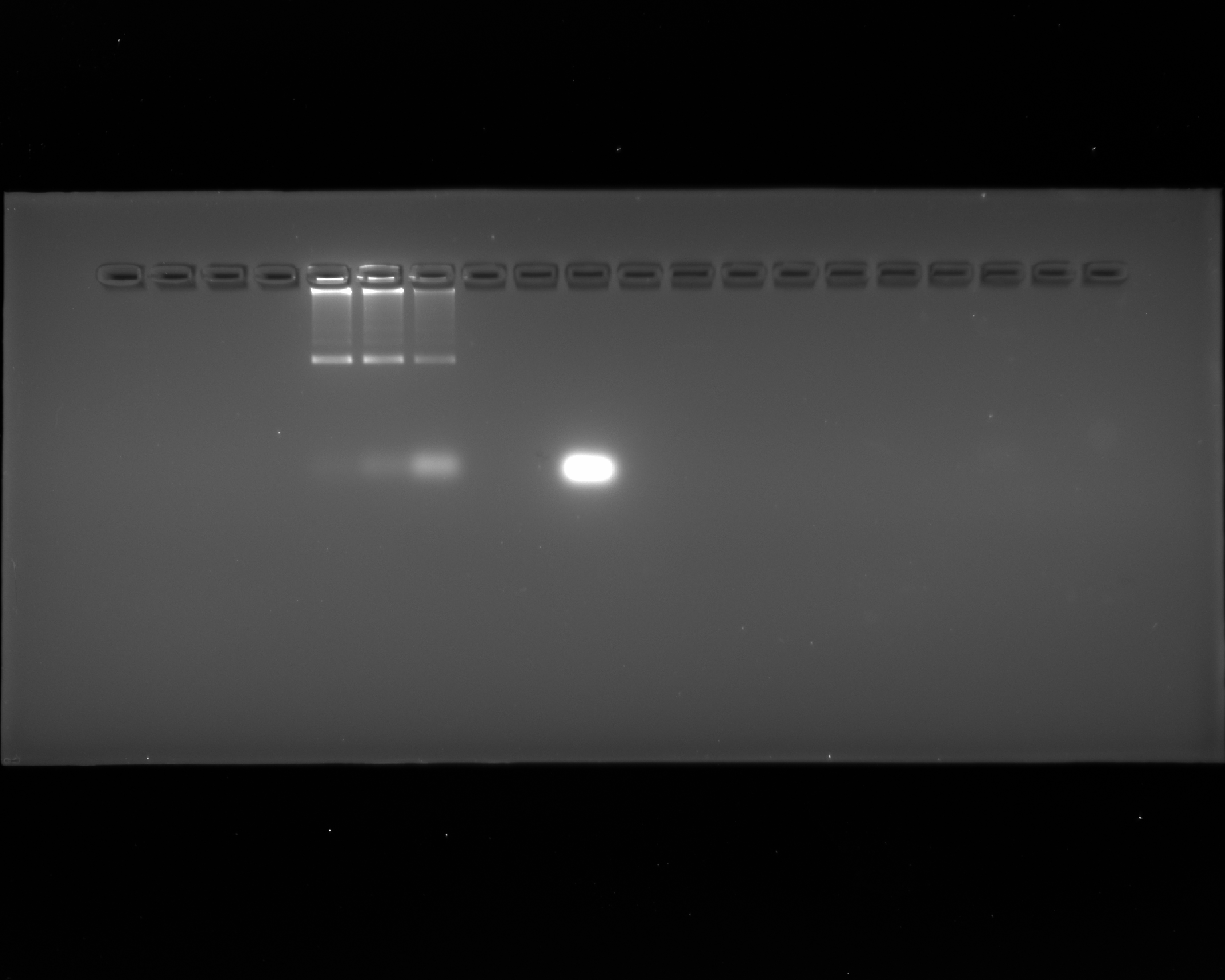

Supplement: NR-016-D4NR01995D-s003 [file NR-016-D4NR01995D-s003.zip › unedited original gel images/SI4c Atto488.tif]

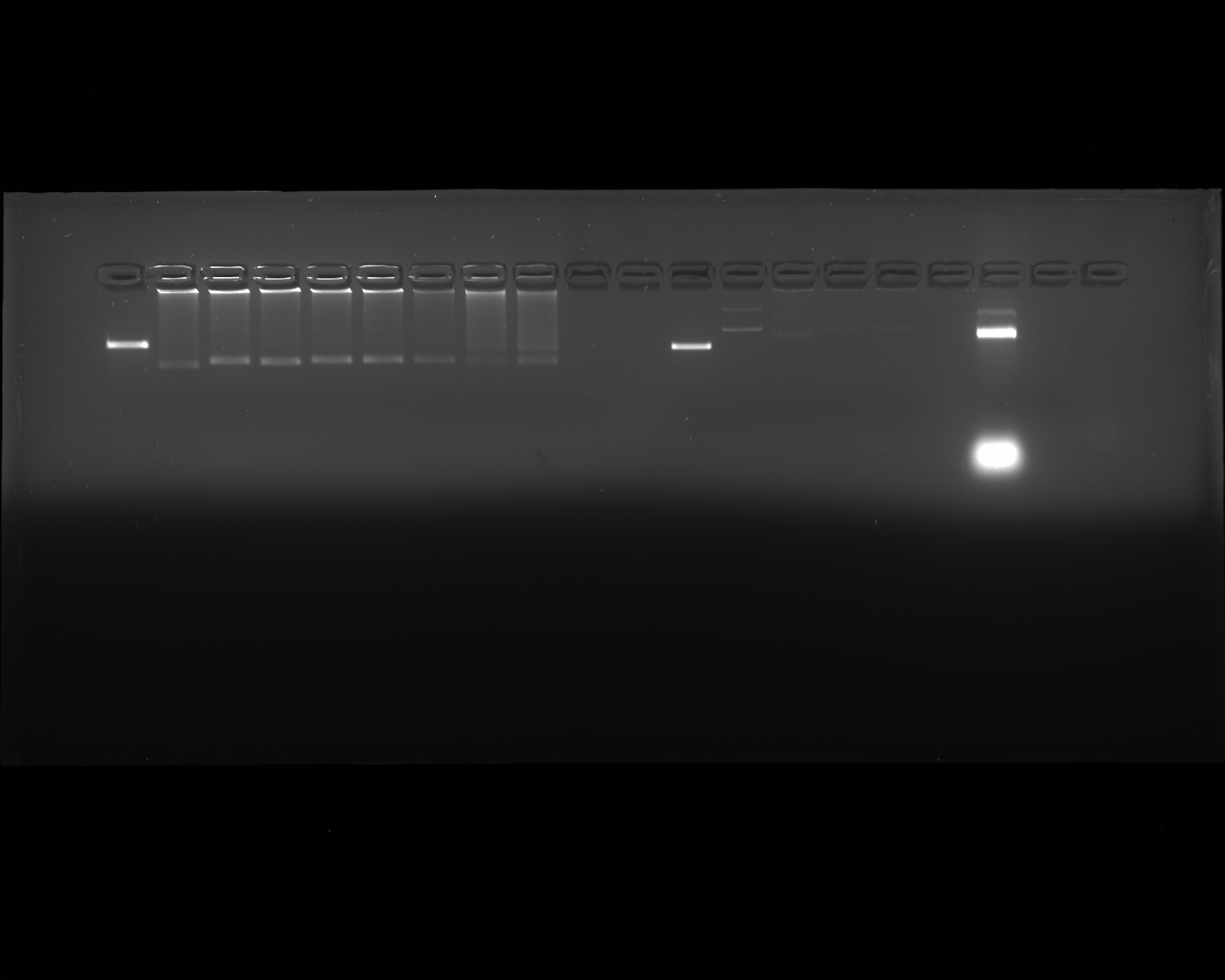

Supplement: NR-016-D4NR01995D-s003 [file NR-016-D4NR01995D-s003.zip › unedited original gel images/SI4c EtBr.tif]

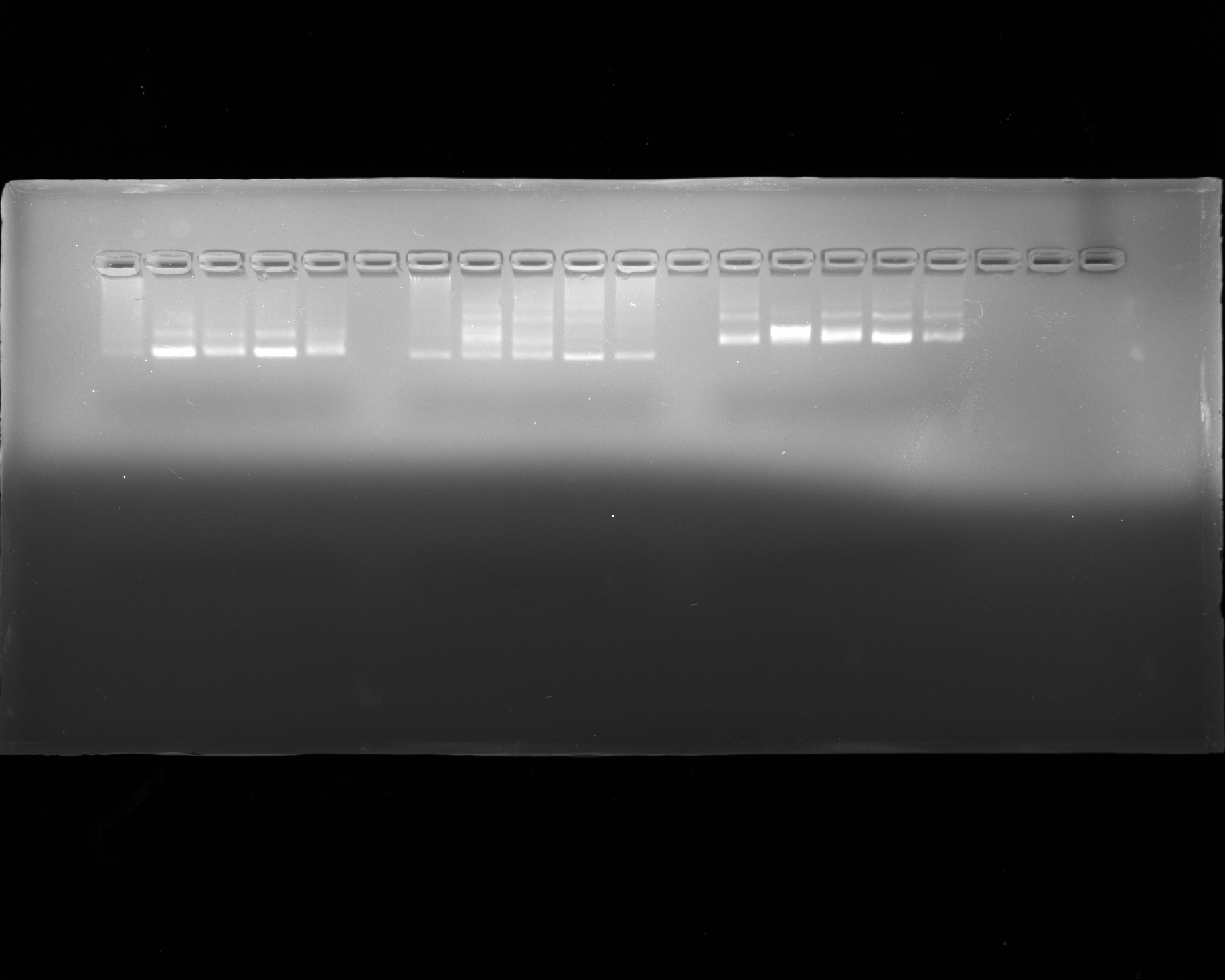

Supplement: NR-016-D4NR01995D-s003 [file NR-016-D4NR01995D-s003.zip › unedited original gel images/SI9 3 weeks 24HB Plate.tif]

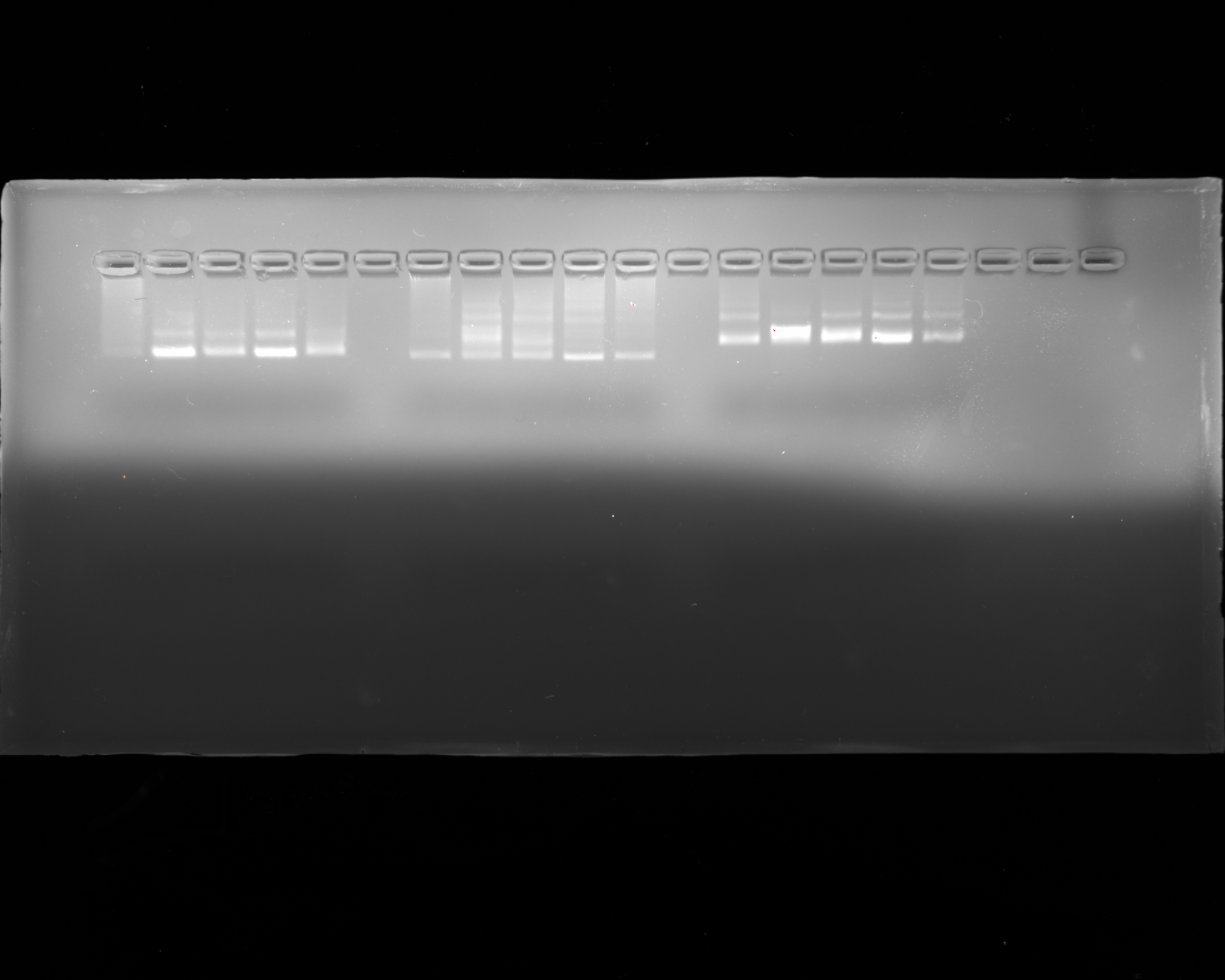

Supplement: NR-016-D4NR01995D-s003 [file NR-016-D4NR01995D-s003.zip › unedited original gel images/SI9 3 weeks 60 HB.tif]

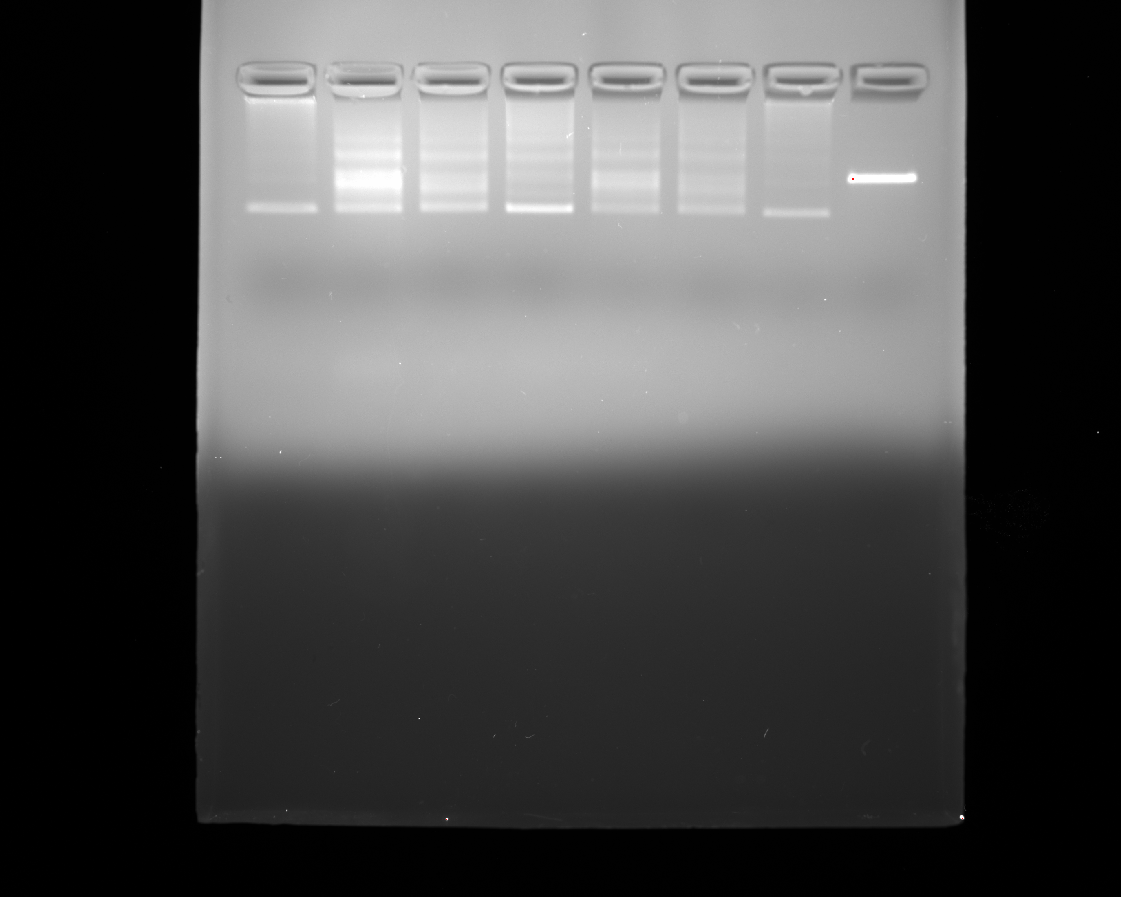

Supplement: NR-016-D4NR01995D-s003 [file NR-016-D4NR01995D-s003.zip › unedited original gel images/SI9 7 weeks 24HB.tif]

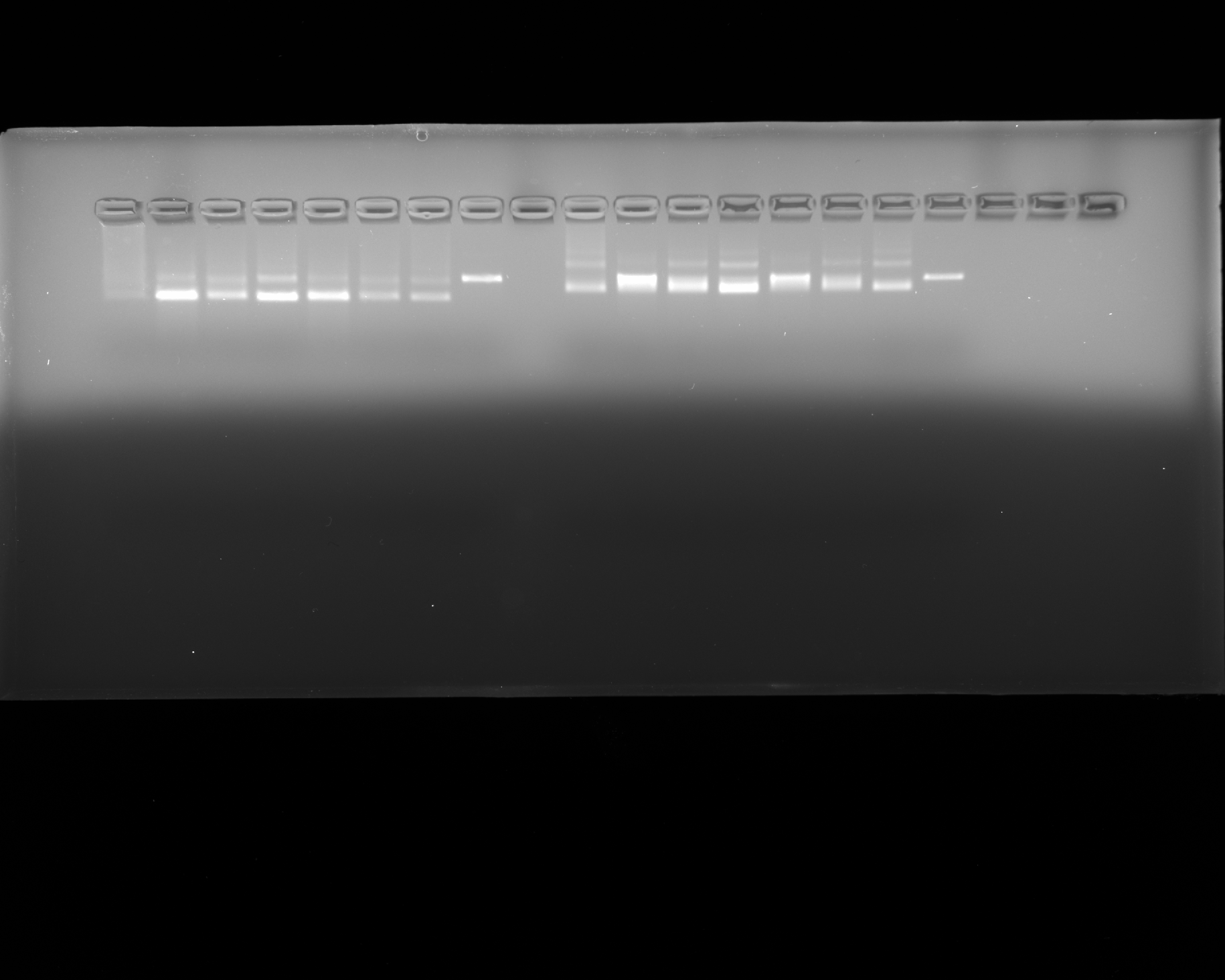

Supplement: NR-016-D4NR01995D-s003 [file NR-016-D4NR01995D-s003.zip › unedited original gel images/SI9 7 weeks 60HB Plate.tif]
